# A *Toxoplasma gondii* Oxopurine Transporter Binds Nucleobases and Nucleosides Using Different Binding Modes

**DOI:** 10.3390/ijms23020710

**Published:** 2022-01-10

**Authors:** Gustavo D. Campagnaro, Hamza A. A. Elati, Sofia Balaska, Maria Esther Martin Abril, Manal J. Natto, Fabian Hulpia, Kelly Lee, Lilach Sheiner, Serge Van Calenbergh, Harry P. de Koning

**Affiliations:** 1 Institute of Infection, Immunity and Inflammation, College of Medical, Veterinary and Life Sciences, University of Glasgow, Glasgow G12 8TA, UK; campagnarogd@gmail.com (G.D.C.); 2218613e@student.gla.ac.uk (H.A.A.E.); sofia_balaska@imbb.forth.gr (S.B.); estermartin95@gmail.com (M.E.M.A.); Manal.Natto@glasgow.ac.uk (M.J.N.); Kelly.lee14@outlook.com (K.L.); lilach.sheiner@glasgow.ac.uk (L.S.); 2 Laboratory for Medicinal Chemistry, Campus Heymans, Ghent University, Ottergemsesteenweg 460, B-9000 Gent, Belgium; fhulpia@its.jnj.com (F.H.); serge.vancalenbergh@ugent.be (S.V.C.); 3 Wellcome Centre for Integrative Parasitology, College of Medical, Veterinary and Life Sciences, University of Glasgow, Glasgow G12 8TA, UK

**Keywords:** *Toxoplasma gondii*, purine transporter, Tg244440, nucleobase transporter, apicomplexan, substrate binding

## Abstract

*Toxoplasma gondii* is unable to synthesize purines de novo, instead salvages them from its environment, inside the host cell, for which they need high affinity carriers. Here, we report the expression of a *T. gondii* Equilibrative Nucleoside Transporter, Tg244440, in a *Trypanosoma brucei* strain from which nucleobase transporters have been deleted. Tg244440 transported hypoxanthine and guanine with similar affinity (*K*_m_ ~1 µM), while inosine and guanosine displayed *K*_i_ values of 4.05 and 3.30 µM, respectively. Low affinity was observed for adenosine, adenine, and pyrimidines, classifying Tg244440 as a high affinity oxopurine transporter. Purine analogues were used to probe the substrate-transporter binding interactions, culminating in quantitative models showing different binding modes for oxopurine bases, oxopurine nucleosides, and adenosine. Hypoxanthine and guanine interacted through protonated N1 and N9, and through unprotonated N3 and N7 of the purine ring, whereas inosine and guanosine mostly employed the ribose hydroxy groups for binding, in addition to N1H of the nucleobase. Conversely, the ribose moiety of adenosine barely made any contribution to binding. *Tg244440* is the first gene identified to encode a high affinity oxopurine transporter in *T. gondii* and, to the best of our knowledge, the first purine transporter to employ different binding modes for nucleosides and nucleobases.

## 1. Introduction

*Toxoplasma gondii* is probably one of the most successful parasites in the world and it is thought to be potentially infective for all warm-blooded land and sea animals. The infection occurs predominantly by ingestion of: meat containing tissue cysts harboring bradyzoites; milk containing tachyzoites; or water or sward contaminated with sporulated oocysts. After ingestion, the parasites penetrate the intestinal tissue, differentiate into tachyzoites and multiply intracellularly by endodyogeny before disseminating to other tissues [1,2]. Infection by *T. gondii* can be lethal for some species of marsupials and sea mammals [3], but in most hosts it is effectively controlled by the immune response and the presence of the parasite is restrained to latent cysts formed in muscle and nervous tissues [1,2,4,5].

Toxoplasmosis highly impacts livestock production by causing abortions in sheep, goats, and pigs, as well as causing diarrhea and anorexia in pigs, which causes the loss of several millions of dollars every year [6]. On top of that, livestock infections very much contribute to human infections as the consumption of meat containing *T. gondii* tissue cysts likely represents the most important source for horizontal transmission to human subjects [2].

Around one-third of the human population is infected by the parasite, but most people are asymptomatic or present mild flu-like symptoms [1]. The complications associated with toxoplasmosis arise predominantly from congenital transmission, especially when first infection with *Toxoplasma* occurs within the first trimester of pregnancy, or after immunosuppression of infected subjects. Vertical transmission during pregnancy often causes miscarriages, death in the uterus, or congenital birth defects such as encephalitis, hydrocephalus, and retinochoroiditis, among others [1,7]. In the case of newly immunocompromised individuals, the decrease in immunocompetence allows the persistent bradyzoites to burst the cyst wall, transform into tachyzoites and once more disseminate around the body, causing encephalitis or retinochoroiditis [1,4]. In fact, toxoplasmosis is the most common infection of the central nervous system in HIV-positive patients [4].

Thus, the current approach to contain toxoplasmosis remains based on chemotherapy, which targets the parasite’s synthesis of folate. Such chemotherapy combines inhibitors of dihydrofolate reductase and dihydrofolate synthase, most notably pyrimethamine (PYR) and sulfadiazine (SULF), respectively, while supplementing with folinic acid to mitigate the toxic effects of PYR [8,9]. The drawbacks with such regimens are toxicity, poor tolerance by immunocompromised patients, prolonged treatment courses, and inefficacy against the latent stage of the parasite [8,9]. Drug resistance can be achieved in vitro and has been reported in clinical isolates [10]. All this highlights the need for the development of new and more effective chemotherapeutical agents with different mechanisms of action against *T. gondii* or for better treatment schemes using currently approved drugs [9,10,11].

The fact that all protozoan parasites studied to date are incapable of producing their own purines, and thus rely on salvage from the host, reinforces the idea of using purine analogues as chemotherapeutical agents against protozoal infections [12,13]. In apicomplexan parasites, the search for purine analogues with chemotherapeutical potential has mainly focused on permeants of the Equilibrative Nucleoside Transporter (ENT) 1 of *Plasmodium falciparum*, the most important (and essential) carrier for purine uptake in this parasite [12,14,15]. However, much less attention has been given to *T. gondii* ENTs, and only a few nucleoside analogues with low micromolar anti-*Toxoplasma* activity have been described to date, specifically 6-benzylthioinosine and its analogues [16,17,18,19,20,21]. In addition, there is one study on the parasitostatic effect of 6-thioxanthine [22] and adenine arabinoside (Ara-A), known to be moderately active against *T. gondii* [23]. Most promising, 2′,3′-dideoxyinosine, which is part of the highly active antiretroviral therapy (HAART) used for HIV-infected patients, has been shown to be active in vitro and in vivo against *T. gondii* [24,25,26].

The antiprotozoal activity of nucleoside analogues depends on their efficient internalization by the parasite and on their affinity for specific metabolic enzymes that activate the analogue or are inhibited by it. The initial studies on *T. gondii* purine transporters by Schwab et al. [27] revealed the existence of at least two transport mechanisms in this parasite, one of which was characterized as a low-affinity adenosine transporter (TgAT1; *K*_m_ 120 μM). The gene believed to encode for TgAT1 was cloned from an adenine arabinoside-resistant strain and expressed in *Xenopus laevis* oocytes, revealing it had similar affinities for inosine, formycin B and allopurinol riboside [28]. The sequence encoding TgAT1 is annotated as Tg244440 in ToxoDB but in this manuscript we will distinguish between the observed low affinity adenosine transport in *T. gondii* tachyzoites, TgAT1, and our result with the expressed sequence Tg244440, so as not to presume a priori that all phenotypic observations assigned to ‘TgAT1’ are necessarily linked to Tg244440. TgAT1 was also identified in isolated tachyzoites by De Koning et al. [29] who further described the presence of high-affinity adenosine (TgAT2; *K*_m_ 0.49 μM) and hypoxanthine (TgNBT1; *K*_m_ 0.91 μM) transporters. It is important that TgAT2 was shown to be a broad specificity nucleoside transporter that recognizes several nucleoside analogues, and is the only *T. gondii* purine transporter for which a substrate binding model has been reported, allowing the rational optimization of analogues with high transport efficiency on this carrier [29].

It is interesting to notice, thus, that although TgAT2 has higher affinity for adenosine than TgAT1 and seems to have broader substrate selectivity, resistance to Ara-A was acquired after loss of TgAT1 [28]. Therefore, we decided to investigate the mode of interaction of TgAT1/Tg244440 with its substrates. To do so, we used a gain-of-function approach utilizing *T. brucei* as the expression system, as previously done by us for ENT family transporters of *T. congolense*, *T. cruzi*, *Leishmania* spp. and *Trichomonas vaginalis* [30,31,32,33]. We found Tg244440 to have high affinity for oxopurine nucleobases and nucleosides and only low affinity for aminopurines and pyrimidines. The very high affinity for both oxopurine nucleosides and nucleobases is highly unusual and the binding modes were carefully investigated using a series of selected matched pair analogues. We conclude that the nucleosides and nucleobases bind in different orientations in the Tg244440 binding pocket.

## 2. Results

### 2.1. Equilibrative Nucleoside Transporters from Coccidian Parasites form a Separate Phylogenetic Cluster within the Family

**Figure 1:**
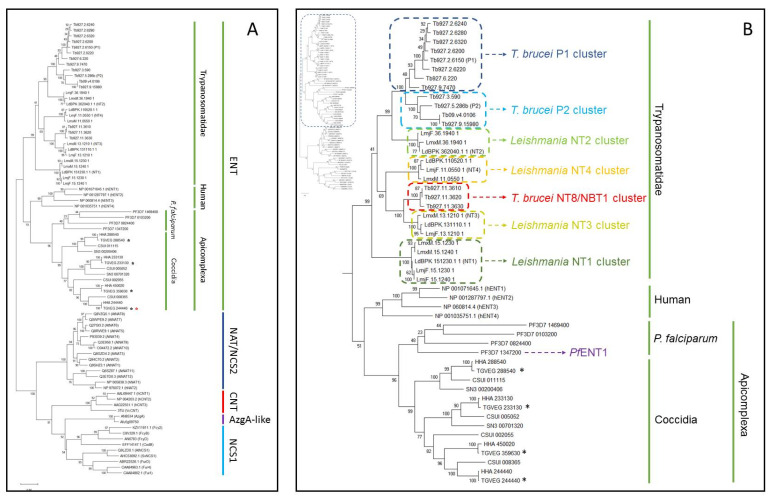
Phylogenetic tree of nucleoside and nucleobase transporters, constructed from a Clustal Omega amino acid multiple alignment by a Maximum Likelihood analysis in Mega-X (500 bootstraps). (**A**) Overview of division into different gene families. (**B**) Zoom-in on the ENT (SLC29) gene family, highlighting some well-characterised transporters. All sequences are listed in Supplemental Table S4. * highlights the position of the *T. gondii* ENT transporters.

We have previously shown that it was possible to predict the substrate selectivity of Trypanosoma cruzi ENT transporters by analysing their phylogenetic alignment with ENTs from other kinetoplastids [32]. Figure 1A shows a phylogenetic tree of nucleoside and nucleobase transporters from a number of different gene families. Transporters belonging to Nucleoside-Cation Symporter 1 (NCS1) and Nucleobase-Ascorbate Transporter (NAT, also known as NCS2), both belonging to the Amino acid-Polyamine-Organocation (APC) superfamily were positioned in a separate branch, but forming two distinct clusters. Members of the Concentrative Nucleoside Transporter (CNT) family formed a separate branch close to the NCS1 transporters as did AzgA-like transporters, although recent analyses have proposed AzgA-like transporters are associated with the NAT family [34,35]. It is probable that the higher number of ENT sequences used for the construction of the phylogenetic tree caused the positioning of AzgA-like and CNT transporters close to the NCS1 cluster.

The ENT sequences formed a consistent branch, with separations between the sequences from trypanosomatids (*T. brucei* and *Leishmania* spp.), humans and apicomplexans (Figure 1B). Among trypanosomatids, which are the best studied protozoans in terms of purine and pyrimidine transport, there was a clear separation between purine nucleoside (*T. brucei* P1 and P2 and *Leishmania* NT2 clusters) and nucleobase (*T. brucei* NT8 and *Leishmania* NT3 and NT4 clusters) transporters, with the purine/pyrimidine nucleoside *Leishmania* NT1 transporters forming an outside group within this branch (Figure 1B). This consistent grouping of transporter sequences with their known activities indicates a high level of predictability in the tree. The apicomplexan ENTs formed a separate group within the ENT family, with a separation between sequences from *P. falciparum* and coccidian apicomplexans (*T. gondii*, *Hammondia hammondi*, *Cystoisospora suis and Sarcocystis neurona*), indicating apparent gene proliferation from a common ancestral ENT gene after divergence of Plasmodium and coccidian species, but *T. gondii* sequences (Figure 1B; black stars) always clustered very closely to their homologues in *H. hammondi*, its closest relative [36]. Moreover, our phylogenetic analysis indicates that Tg244440 (and its homologue in *H. hammondi*) was the most distant of the ENT sequences found in coccidia (Figure 1A; red star), being at the edge of the ENT clade. We also observed an extraordinary conservation of the Tg244440 protein sequence among *T. gondii* strains: of the sequences retrieved from 15 strains, only one amino acid change (Leu → Phe in position 304) in Tg244440 was observed and only in strain MAS (Supplementary Figure S1). This high a level of conservation was not observed for any other *T. gondii* ENT transporter. Full-length Tg288540 (696 a.a.) was present in 14/15 of these strains, with the allele in strain TgRUB lacking the N-terminal 272 a.a.; Tg233230 (531 a.a.) was found in all 15 strains and displayed four amino acid changes; Tg359630 was found in only 11/15 strains available at ToxoDB, and displayed six amino acid changes within those sequences, while lacking the N-terminal 308 a.a. in strain RH (Supplementary File S1).

### 2.2. Tg244440 Is a High-Affinity Oxopurine Transporter

**Figure 2:**
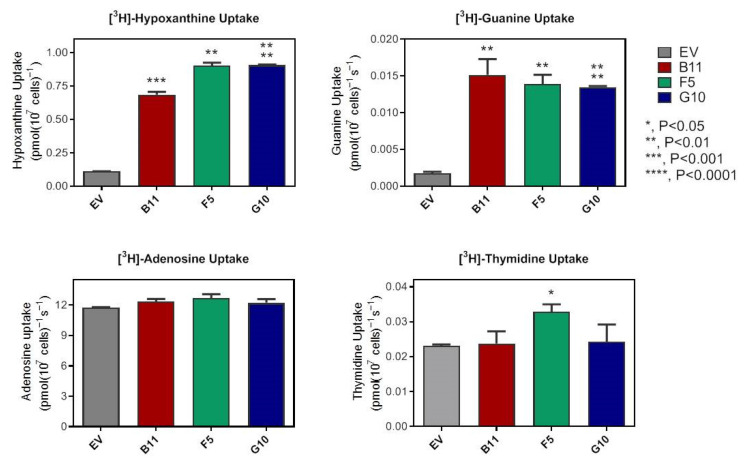
Uptake of radiolabelled nucleosides and nucleobases by in three clonal lines of *T. brucei* NB-KO procyclics, expressing Tg244440 in pHD1336. EV, empty vector control. Radiolabel concentration was 0.1 µM and uptake was measured over 60 s, in triplicate (average and SEM shown). Statistically significant differences between the control and the Tg244440-expressing cells were established using Student’s unpaired *t*-test. *, *p* < 0.05, **, *p* < 0.01, ***, *p* < 0.001.

To understand the role of Tg244440 in purine transport, we transfected this gene into procyclic forms of the *T. brucei* TbNB-KO strain that has a null background for the uptake of guanine (and a much-reduced background for hypoxanthine uptake), through the knockout of the locus containing the three nucleobase transporters Tb927.11.3610, Tb927.11.3620, Tb927.11.3630 [32,36]. *T. brucei* also have a near-null background for thymidine uptake at low concentrations [37,38]. Three clones transfected with Tg244440 (B11, F5 and G10) were randomly picked and used for a screen of substrate transport ability, using 0.1 μM of either [^3^H]-adenosine, [^3^H]-guanine, [^3^H]-hypoxanthine or [^3^H]-thymidine for 60 s, in comparison to TbNB-KO transfected with empty pHD1336 (EV) (Figure 2). All three TbNB-KO + Tg244440 clones showed highly significant increases in the levels of guanine and hypoxanthine uptake whereas uptake of thymidine was only slightly increased, and only in clone F5. No difference in the transport of adenosine was observed but TbNBT-KO procyclics retain the high-affinity adenosine/inosine/guanosine P1 transporters [39], which gives a high background in the assays using radiolabelled adenosine, as evidenced by the high uptake rate for adenosine compared to the other [^3^H]-permeants (Figure 2). Clone F5 was selected for all further experimentation.

**Figure 3:**
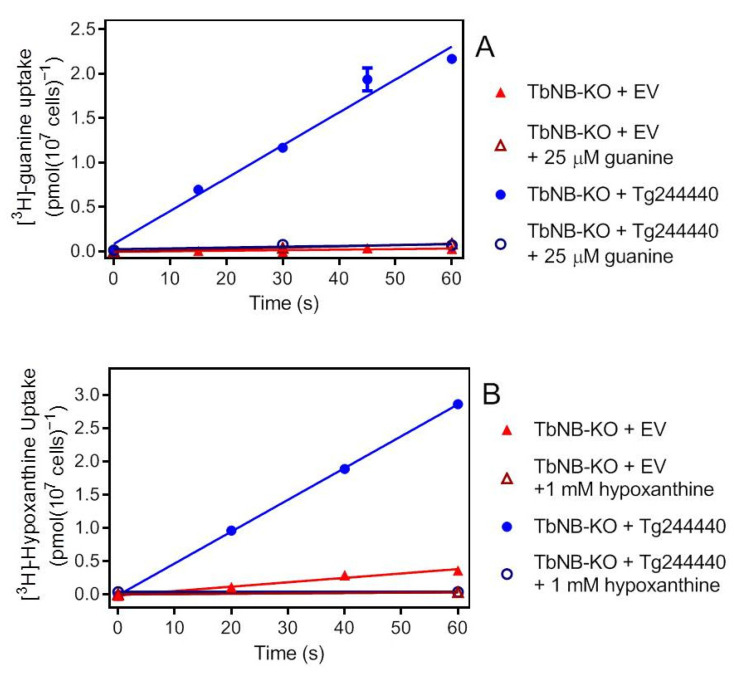
Uptake of oxopurine nucleobases by Tg244440 expressed in *T. brucei* NB-KO procyclics. (**A**) Uptake of 0.05 µM [*^3^*H]-guanine over 60 s. The rate of uptake in the cells expressing Tg244440 was 0.037 pmol(10^7^ cells)^−1^s^−1^ and 0.00059 in the EV control. Both rates were not significantly non-linear (*p* > 0.90, runs test); uptake in the control was not significantly non-zero (*p* = 0.11, F-test), whereas the rate in +Tg244440 cells was significantly non-zero (*p* = 0.0012), but not in the presence of 25 µM unlabelled guanine (*p* = 0.40). (**B**) Uptake of 0.10 µM [*^3^*H]-hypoxanthine over 60 s. Rate was 0.0066 and 0.048 pmol(10^7^ cells)^−1^s^−1^ for EV control and +Tg2444440 cells, respectively. Both lines were not significantly non-linear (*p* > 0.99) and significantly non-zero (*p* = 0.012 and *p* < 0.0001, respectively). Each symbol represents the average and SEM of triplicate determinations.

In order to determine the linear phase of substrate uptake we incubated TbNB-KO cells expressing Tg244440 or transfected with the empty vector pHD1336 with 50 nM of [^3^H]-guanine or 100 nM of [^3^H]-hypoxanthine for predetermined time intervals. The uptake of [^3^H]-guanine in the cells expressing Tg244440 was linear for 60 s (Table 1, Figure 3A). In the presence of 25 µM unlabelled guanine there was no significant uptake of the label. In the control cells without the Tg244440 transporter (empty vector, EV), the uptake over 60 s was also not significantly non-zero (Table 1).

Hypoxanthine uptake was very similar to that of guanine: linear over 60 s, saturable, and with a significantly non-zero rate (Table 1). The slope of the EV control was also non-zero, as previously reported [32] but 86.2% lower than the +Tg244440 clone F5 (*p* < 0.0001) (Figure 3B). We conclude that Tg244440 efficiently transports guanine and hypoxanthine but at best marginally transports thymidine, while no conclusion on adenosine uptake could be reached.

**Figure 4:**
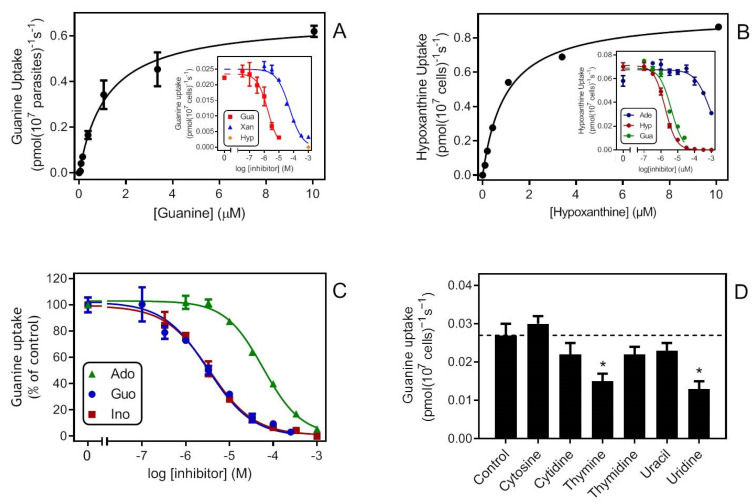
(**A**) Uptake of 0.05 µM [^3^H]-guanine over 30 s. Main graph: Michaelis-Menten saturation curve. Inset: shows the dose-dependent inhibition curves of the same experiment, for guanine (Gua), xanthine (Xan) and a single saturation point for hypoxanthine (Hyp). (**B**) Uptake of 0.10 µM [^3^H]-hypoxanthine over 30 s. Main graph: Michaelis-Menten saturation curve. Inset: dose-dependent inhibition curves of the same experiment (ade = adenine). (**C**) Inhibition of 0.05 µM [^3^H]-guanine over 30 s by unlabelled purine nucleosides adenosine (Ado), guanosine (Guo) and inosine (Ino). (**D**) Uptake of 0.05 µM [^3^H]-guanine over 30 s inhibited by pyrimidine nucleosides or nucleobases. Each bar is the average and SEM of 3–5 independent experiments, each performed in triplicate. Statistical difference with the control (empty vector pHD1336) was determined by Student’s unpaired *t*-test. *, *p* < 0.05.

We thus proceeded to determine the affinity of Tg244440 for hypoxanthine and guanine, and found it to have equally high affinity for both oxopurine nucleobases, with *K*_m_ values of 0.79 ± 0.06 μM and 1.26 ± 0.16 μM, respectively (n = 3, not significantly different, *p* = 0.099, unpaired *t*-test) (Figure 4A,B). Tg244440 showed a clear preference for oxopurine nucleobases, as it interacted better with xanthine (Figure 4A), with a *K*_i_ of 42.4 ± 4.2 μM (n = 3), than with adenine, which was poorly recognised by the transporter (Figure 4B), with a *K*_i_ of 425 ± 80 µM (n = 8) (Table 2).

Interestingly, purine nucleosides were also recognised by the transporter and showed the same trend observed for their respective nucleobases: guanosine and inosine were strongly preferred over adenosine. Indeed, as for hypoxanthine and guanine, the transporter displayed statistically identical *K*_i_ values for inosine and guanosine (4.05 ± 1.37 μM (n = 4) and 3.30 ± 0.3 μM (n = 7), respectively), only slightly higher than the *K*_m_ for their corresponding nucleobases, whereas the affinity for adenosine was about 20-fold lower (Figure 4C). In contrast, Tg244440 displayed 6-fold higher affinity for adenosine than for adenine (Table 2).

Pyrimidine nucleobases and nucleosides showed very little affinity for Tg244440 as none of them was able to completely block the transport of 0.1 μM [^3^H]-guanine at 1 mM and only uridine and thymine displayed any significant inhibition at this concentration (*p* < 0.05), 10,000-fold higher than that of the radiopermeant (Figure 4D). We thus conclude Tg244440 is a high-affinity oxopurine transporter, with particular affinity for guanine and hypoxanthine, that poorly recognises aminopurines and shows no affinity for pyrimidines at physiologically relevant levels.

Frames A–C: Single experiments in triplicate, representative of three or more identical experiments; for number of replicate determinations for each inhibitor (Table 2).

### 2.3. Tg244440 Binds Purine Nucleobases and Nucleosides in Different Manners

In order to better understand the affinity of Tg244440 for oxopurines over aminopurines, and its intriguing higher affinity for adenosine than for adenine, we sought to investigate which functional groups of the substrates are involved in the interactions with the transporter. To do so, several commercial and newly synthesised analogues of purine nucleobases and nucleosides, with modifications at different positions, were used in competition to 50 nM of [^3^H]-guanine to determine the Gibbs free energy (ΔG^0^) of each interaction between transporter and substrate, exactly as described for multiple other transporters [29,35,40,41,42,43]. All *K_i_* and ΔG^0^ values are listed in Table 2.

#### 2.3.1. Binding of Purine Nucleobases

Given the difference in the high affinity of Tg244440 for guanine and hypoxanthine as opposed to the low affinity for adenine, we first investigated the participation of the keto group on C6 and of the protonation state of N1. We found that the presence of the 6-keto group was not important per se given that 6-mercaptopurine and 6-thioguanine (Table 2), which form double bonds with C6 that keep N1 protonated, showed statistically identical *K_i_* values as hypoxanthine (*p* = 0.17) and guanine (*p* = 0.35), respectively, although the sulphur is unable to make a strong hydrogen bond. In contrast, 6-chloropurine, in which N1 is unprotonated, was a poor inhibitor of Tg244440 compared to hypoxanthine (*p* = 5.8 × 10^−7^) and displayed a large difference in Gibbs free energy (δ(ΔG^0^ = 9.6 kJ/mol)), consistent with the loss of an H-bond involving protonated N1. To verify this conclusion we determined the affinity of Tg244440 for 1-methylhypoxanthine and 1-deazahypoxanthine, and found a similar loss in Gibbs free energy (9 kJ/mol and 11 kJ/mol, respectively, relative to hypoxanthine, Table 2).

We previously observed good in vitro and in vivo antiprotozoal potential of purine nucleobase analogues carrying a toxophore on C2 [44,45], and we thus decided to investigate whether Tg244440 could tolerate different C2 modifications. The 2-amine did not interact positively or negatively with the transporter as the affinities for guanine and hypoxanthine were almost identical (*p* = 0.10), as were the *K_i_* values of adenine and 2,6-diaminopurine (*p* = 0.17). Xanthine, featuring a 2-keto group, did display a significantly lower *K_i_* value than hypoxanthine (*p* = 7.8 × 10^−6^, δ(ΔG^0^) = 7.3 kJ/mol) but this could be the result of the protonation of N3 in xanthine. Indeed, 3-deazahypoxanthine displayed a similar loss in binding energy (δ(ΔG^0^) = 8.1 kJ/mol), which confirms the positive contribution of N3 as an H-bond acceptor. Furthermore, the observation that 3-methylxanthine displayed somewhat higher affinity than xanthine (δ(ΔG^0^) = 2.7 kJ/mol; *p* = 0.006) was also consistent with unprotonated N3 being the form contributing to binding. Thus, we conclude that the presence of small substitutions on C2 does not affect the interaction with Tg244440 as long as N3 remains unprotonated.

N7 was also found to be involved in the binding of substrates to Tg244440 as 7-deazaguanine (*p* = 0.0028) and 7-deazahypoxanthine (*p* = 6.1 × 10^−5^) showed significantly lower affinity for the transporter (δ(ΔG^0^) = 9.1 and 12.3 kJ/mol, respectively). Moreover, a Nitrogen atom shift from position seven of hypoxanthine to position eight (allopurinol) caused a significant drop in affinity (*p* = 1.2 × 10^−6^), which resembles that of 7-deazaadenosine (*p* = 0.092), showing that a nitrogen at position eight is unable to make a similar H-bond as N7. Interestingly, the addition of a bromide on C7 of allopurinol (7-Br-allopurinol) recovered 2.5 kJ/mol in Gibbs free energy, similar to what has been observed for the *T. brucei* P1 transporter in which the addition of 7-bromo substantially improves the affinity for 3′-deoxytubercidin [46]. The addition of an extra nitrogen on position eight (8-azahypoxanthine) seems to be highly deleterious to binding by the transporter, as 1 mM of 8-azahypoxanthine failed to inhibit even 50% of the transport of 50 nM [^3^H]-guanine.

**Figure 5:**
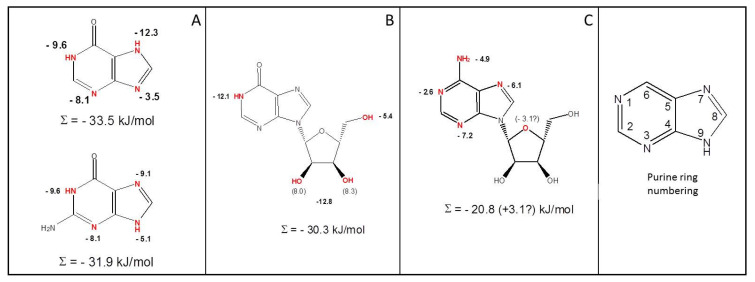
Substrate binding models for Tg244440. (**A**) Hypoxanthine (top) and guanine (bottom). (**B**) Inosine. (**C**) Adenosine. Inset: numbering of purine ring positions. Oval dashed lines represent proposed bonds, presumed to be hydrogen bonds; the numbers given with them are the estimated Gibbs free energy of interaction, in kJ/mol.

The 9-deaza analogues of guanine and hypoxanthine also displayed significantly lower affinity than their 9-aza counterparts (*p* = 0.012, 5.1 kJ/mol and *p* = 5.1 × 10^−5^, 3.5 kJ/mol, respectively). It should be noted that the protonation state of the purine ring nitrogens is part of tautomeric equilibria that are functions of pH and buffer composition, and may be somewhat different in guanine and hypoxanthine, and could contribute to the small differences in δ(ΔG^0^) in their 7-deaza and 9-deaza analogues. However, the sum of δ(ΔG^0^) for the 7-deaza and 9-deaza analogues was very similar for guanine and hypoxanthine at 14.2 and 15.8 kJ/mol, respectively. Figure 5A shows a model for the binding modes of hypoxanthine and guanine. Summation of the Gibbs free energy of the four proposed interactions yields totals of −33.5 and −31.9kJ/mol, respectively, very close to the ΔG^0^ values directly calculated from their respective *K_i_* and *K_m_* values obtained with [^3^H]-guanine and [^3^H]-hypoxanthine (Table 2).

#### 2.3.2. Binding of Purine Nucleosides

The Σ(δ(ΔG^0^)) values for hypoxanthine and guanine are also close to the values of the corresponding nucleosides inosine and guanosine, in which, however, N9 would be unable to make any contribution through hydrogen bonds. In *T. brucei*, the P2/TbAT1 aminopurine transporter binds adenosine only through the adenine moiety, tolerating rather than requiring the ribose half of the molecule [40,47]. For TbAT1, the ribose hydroxyl groups and ring oxygen do not make a positive contribution to nucleoside binding—indeed 2′-deoxy and 3′-deoxyadenosine display somewhat higher affinity than adenosine, virtually identical to adenine [40,48]. To test whether the nucleoside binding mode of Tg244440 is similar to that situation, with at most a minor contribution to binding from the ribose moiety, we systematically determined the affinity of matched pair deoxy-inosine and deoxy-guanosine analogues.

Interestingly, inosine analogues lacking 2′-OH, 3′-OH or 5′-OH showed significantly lower affinity for Tg244440, with losses of 8.3 kJ/mol (*p* = 0.0049), 8.0 kJ/mol (*p* = 3.0 × 10^−6^) and 5.4 kJ/mol in Gibbs free energy (*p* = 3.2 × 10^−4^), respectively, compared to inosine. Similar results were shown by 2′-Deoxyguanosine and 3′-deoxyguanosine with loss of 11 kJ/mol (*p* = 2.9 × 10^−12^) and 9.4 kJ/mol in Gibbs free energy (*p* = 1.9 × 10^−8^), respectively, relative to guanosine (Table 2). These results show that most likely all three hydroxy groups of oxopurine nucleosides interact with the transporter. The removal of both 2′-OH and 3′-OH (i.e., 2′,3′-dideoxyinosine, ddI) caused a loss of Gibbs free energy of 12.8 kJ/mol compared to inosine (*p* = 4.9 × 10^−5^), which is less than the sum of the energy lost in 2′-deoxyinosine and 3′-deoxyinosine (16.3 kJ/mol). The likely explanation for this is that both hydroxy groups interact with the same amino acid(s) in the binding pocket and the absence of either allows a higher energy interaction with the other. Figure 5B therefore shows a contribution of −12.8 kJ/mol for the 2′ and 3′-hydroxys to the binding of inosine, for which a total ΔG^0^ of −30.3 kJ/mol was determined (Table 2). The difference, 17.5 kJ/mol, is likely the result of one or more interactions with the hypoxanthine base group.

Given the low affinity of Tg244440 for adenosine, we first investigated the -sixth position. Substituting the keto group by a sulphur on C6, creating 6-thioinosine, had no significant effect on affinity (*p* = 0.18) but the Gibbs free energy of interaction for nebularine (9-(β-D-ribofuranosyl)purine) was much lower than for inosine (11.9 kJ/mol, *p* = 0.0014). The importance of N1(H) for binding of inosine was confirmed by the ΔG^0^ for 1-deazainosine, which was virtually identical to that of nebularine (18.7 versus 18.9 kJ/mol, *p* = 0.78). In contrast to the binding of the oxopurine nucleobases, position N7 is certainly not positively involved in the binding of inosine as the *K_i_* values for 7-deaza analogues of inosine (*p* = 0.29), of 2′-deoxyinosine (*p* = 0.019) and of 3′-deoxyinosine (*p* = 0.25) were not significantly different for Tg244440 than those of their matched pairs (Table 2). Moreover, 7-Cl and 7-Br analogues of 7-deazainosine still presented binding energies highly similar to inosine (*p* = 0.16 and 0.088, respectively). The further modification of *O*-methylation and *O*-ethylation of 7-Cl,7-deazainosine did, however, cause a significant reduction in binding energy, as this changed the protonation state of N1 (δ(ΔG^0^) = 5.7 and 9.2 kJ/mol, *p* = 1.4 × 10^−4^ and 2.0 × 10^−5^, respectively). However, the existence of an (energetically unfavourable) tautomeric state resulting in N1 being a hydrogen bond acceptor (as opposed to a hydrogen bond donor in inosine) in the *O*-alkylated analogues gives these ligands a higher binding energy than 1-deazainosine (δ(ΔG^0^) = 6.4 and 2.8 kJ/mol, *p* = 0.0018 and 0.0060, respectively).

These observations show that the base parts of oxopurine nucleosides interact with Tg244440 through only a single H-bond, to the protonated N1 (Figure 5B), giving a Σ(ΔG^0^) of −30.1 kJ/mol for inosine, very close to the experimental value of −30.8 kJ/mol. It is abundantly clear from the binding model presented in Figure 5 that the interaction of the oxopurine base with Tg244440 is very different for inosine and for hypoxanthine/guanine and that the oxopurine nucleosides are predominantly bound through interactions with the ribose hydroxy groups.

The aminopurines adenosine and adenine displayed only moderate to low affinity for Tg244440, with the *K_i_* for adenosine significantly lower than for adenine (*p* = 0.0083). Curiously, the removal of 2′-OH (*p* = 0.067), 3′-OH (*p* = 0.46) or 5′-OH (*p* = 0.024) of adenosine barely affected its binding to Tg244440 (Table 2), suggesting that they are not involved in the binding of this nucleoside to the transporter. Although it could alternatively be speculated that 2′- and 3′-deoxyadenosine bind to Tg244440 in slightly different orientations and compensate for the absence of each other, it remains unclear how the ribose moiety contributes to adenosine binding, perhaps through the ring oxygen, but clearly the adenosine binding mode is different from the binding mode of inosine.

Using a series of adenosine analogues, significantly lower affinities were found for nebularine (*p* = 0.7.4 × 10^−4^), 1-deazaadenosine (*p* = 2.1 × 10^−5^), 3-deazaadenosine (*p* = 0.0011) and 3′-deoxy,7-deazaadenosine (*p* = 0.4.6 × 10^−4^) (Table 2). Given that 3′-hydroxy appears not to be involved in the binding, the above leads to a model of adenosine binding, with Σ(δ(ΔG^0^)) = −20.8 kJ/mol for the base group and the balance to the observed ΔG^0^ (3 kJ/mol), presumably through the unknown interaction with the ribose moiety (Figure 5C). Interestingly, adenine arabinoside (Ara-A) displayed significantly lower Gibbs free energy of binding than adenosine by 3.75 kJ/mol (*p* = 1.9 × 10^−6^), raising the possibility that the stereochemical inversion of the 2′-OH interferes with a positive interaction with Tg244440. The binding mode of adenine, being of low affinity, was not investigated in detail.

## 3. Discussion

Every living cell requires purines and pyrimidines as building blocks for their nucleic acids, as energy storage and as intra- and extracellular signalling molecules. *T. gondii*, like other parasitic protozoa, is unable to synthesise its own purines and relies on the uptake of these nutrients from the host, a property that has been explored for the development of antiparasitic purine analogues [12,16,18,49]. The ability of *T. gondii* to incorporate exogenous purines, specifically adenosine, has been known for almost four decades [27,50], but the molecular mechanisms of its salvage process are poorly understood.

In its intracellular milieu, *T. gondii* will have to compete with the host’s enzymes for the available nutrients, and free nucleobases and unphosphorylated nucleosides are present at very low concentrations. Thus, intracellular parasites must have evolved nucleobase and nucleoside transporters with particularly high affinity for their substrates, as recently shown for Trypanosoma cruzi [32] and before for *Leishmania* spp. [42,51], and for the unrelated apicomplexan Plasmodium falciparum [14].

Only two high-affinity purine transporters, TgNBT1 and TgAT2, selective for hypoxanthine and adenosine/inosine, respectively, have been described for *T. gondii* tachyzoites [29]. In addition, a low-affinity adenosine/inosine transporter (TgAT1) with *K_m_* values > 100 μM for its substrates has been described by multiple researchers [27,28,29]. It seems unlikely, however, that TgAT1 would be effective for adenosine uptake, given that its *K_m_* for adenosine is higher than the *K_m_* values of human adenosine kinase (0.2–0.4 μM), human adenosine deaminase (49 μM) and the main adenosine transporter hENT1 (40–60 μM) [52,53,54,55].

Schwab et al. [27] had already reported that *T. gondii* tachyzoites are able to rapidly transport inosine and hypoxanthine, and they described an inosine transporter with an apparent *K_m_* of 81 μM; however, the authors also noticed that inosine can be internalised via two different mechanisms. One inosine transport activity was sensitive and one was insensitive to inhibition by adenosine; the latter could be inhibited by high concentrations of formycin B or hypoxanthine.

Chiang et al. [28] then reported that the genes encoding adenosine kinase and TgAT1 (whose cloned sequence is the gene annotated as Tg244440 in ToxoDB.org) are essential for susceptibility to Ara-A, and that TgAT1 is the sole adenosine transporter of *T. gondii* tachyzoites. Upon expression of Tg244440 in Xenopus laevis oocytes, the authors determined an adenosine *K*_m_ of 114 ± 37 µM, using 10 µM [^3^H]-adenosine. This report has always been puzzling to us, as *T. gondii* tachyzoites would be expected not to rely on a very low affinity transporter for their purine salvage. Moreover, although the affinity of TgAT1 for Ara-A had never been measured directly, it is unlikely that the transporter would have higher affinity for this adenosine analogue than for adenosine itself, and therefore the *K*_m_ of TgAT1 for Ara-A would have been (at least) around the hundreds of micromolar range, which is confirmed here. Thus, it would be expected that TgAT2, which has much higher adenosine transport capacity than TgAT1 and interacts with Ara-A with a *K*_i_ of 2.9 μM [29], was even more important in the internalisation of this nucleoside analogue.

The use of high concentrations of radiolabel used in some of the previous studies [27,28] allows only for the detection and characterisation of low affinity transporters, as it saturates the higher affinity carriers. This is particularly a limitation of the 1995 study [27], which used 1 µM of [^3^H]-adenosine, a concentration too high to reliably detect TgAT2 with its reported *K*_m_ of 0.49 µM [29], leaving only the low affinity flux. Moreover, the concentration of 20 μM of inosine that was used to determine the *K_m_* of the inosine transporter(s) expressed by tachyzoites would have completely saturated a high-affinity transporter like TgAT2 (*K*_m_ of 0.77 µM [29]) and make its identification impossible. Here, we start to address the confusing state of the (all too sparse) literature on this subject, with a thorough characterisation of the Tg244440 gene, this time expressed in a protozoan cell type with a null background for guanine uptake.

Using radiolabelled substrate concentrations of 50 and 100 nM for guanine and hypoxanthine, respectively, we determined that the transporter encoded by Tg244440 has *K*_m_ values of approximately 1 μM for these purine nucleobases. Similarly, this transporter was inhibited, with almost as high affinity, by the oxopurine nucleosides inosine and guanosine but only by much higher concentrations of the aminopurines, adenosine and adenine. We therefore conclude that Tg244440 is an oxopurine-specific transporter, although the adenosine *K*_m_ reported by Chiang et al. [28] in X. laevis oocytes is consistent with the value we report here.

The slightly lower affinity for the oxopurine nucleosides over their corresponding bases leads naturally to a hypothesis that Tg244440 is a nucleobase transporter that largely tolerates the N(9)-β-D-ribofuranose. However, the higher affinity of adenosine over adenine does not fit this hypothesis and competition experiments with 2′-, 3′- and 5′-deoxyinosine and/or -guanosine showed that the oxopurine nucleosides derive most of their binding energy from interactions through these hydroxy groups. Equally, systematic investigations of the purine ring interactions found that the oxopurine nucleobases interacted with Tg244440 through all four nitrogen positions (N1(H); N3; N7 and N9(H)), presumably with hydrogen bonds, while in the corresponding nucleosides probably only N1(H) contributed positively to binding. Furthermore, the binding mode for adenosine was different again, with little apparent involvement of the ribose hydroxy groups but binding contributions from N1, N3, N7 and 6-NH2. The 4.5 kJ/mol higher binding energy for adenosine over adenine might be attributable to an interaction with the ribose group but we were unable to ascertain the nature of that interaction. The transporter had at best very low affinity for pyrimidine nucleobases and nucleosides.

It is clear that Tg244440 can bind different classes of purine bases and nucleosides in different ways and is the first protozoan transporter documented to do so. Logically, the transporter either has multiple binding sites or allows the different substrates to orient differently in a single binding pocket. Of these possibilities the former seems unlikely as all the inhibition data were consistent with competitive inhibition and consistently displayed Hill slopes very close to −1. Moreover, current structural models of ENT family transporters [56] do not allow for multiple nucleoside and/or nucleobase binding sites but do show a rather large substrate-binding cavity deep inside the 11-TMD structure. Interestingly, this allows different binding orientations for the classical inhibitors S-(4-nitrobenzyl)-6-thioinosine (NBMPR) and dilazep, using different but overlapping binding sites including the nucleoside-binding pocket [56]. In an example from the Nucleobase Ascorbate Transporter (NAT) family, different binding orientations for xanthine and allopurinol were demonstrated for the UapA transporter of *Aspergillus nidulans* [57]. Mutations in the binding pocket of this transporter were shown to alter both substrate specificity and substrate orientation [58]. Similarly, a recent study showed that a series of mutations applied to the *Escherichia coli* XanQ transporter in order to mimic the ancestral XanQ (AncXanQ) altered it from a xanthine-specific carrier to a xanthine/guanine transporter [59]. Moreover, molecular docking simulations predicted two energetically favorable AncXanQ-guanine binding modes, both of which differed from the mode of interaction between AncXanQ and xanthine [59].

We conclude that Tg244440 is a broad-specificity, high affinity transporter for oxopurine bases and nucleosides and that this is likely enabled by a versatile architecture of its central binding pocket. As such, the transporter may serve as a conduit for therapeutic oxopurine analogues tailored to its unique selectivity profile and the binding models here presented. However, 2′,3′-dideoxyinosine, which has been shown to have in vitro and in vivo activity against *T. gondii* [24,25,26], displayed a *K*_i_ of almost 700 μM for Tg244440 and is therefore unlikely to be an efficient substrate of this carrier. Equally, the adenosine analogue Ara-A which is known to have moderate activity against *T. gondii* [23], also displayed low affinity for Tg244440. Nevertheless, much progress has recently been made in the identification of nucleoside analogues with highly promising antiprotozoal activities [12], including 7-deaza inosines [60], 3’-fluorinated 7-deazapurine nucleosides [61], 7-phenyl tubercidins [62] and pyrazolo[3,4-d]pyrimidine nucleosides [63]. Additionally, it is worth noting that 7-chloro-7-deazainosine and 7-bromo-7-deazainosine displayed high affinity for Tg244440. Our current activities include the characterisation of the other three *T. gondii* ENT transporters and the identification of purine nucleoside antimetabolites against toxoplasmosis.

## 4. Materials and Methods

### 4.1. In Vitro Culture of T. gondii

*T. gondii* tachyzoites of the RH strain were grown in Human Foreskin Fibroblasts (HFF) and maintained in Dulbecco’s Modified Eagle’s (DMEM; Life Technologies, Paisley, Renfrewshire, UK) supplemented with 10% fetal bovine serum and a cocktail of 1% (*v*/*v*) penicillin-streptomycin and L-glutamine (all from Life Sciences), at 37 °C under 5% CO_2_.

### 4.2. mRNA Isolation and cDNA Synthesis

Parasites released from infected cells were pelleted by centrifugation and the RNA was extracted using the RNeasy Mini kit (Qiagen, Manchester, UK) following the manufacturer’s recommendations. Any potential contaminant DNA was eliminated by using the TURBO DNA-free Kit (Thermo Fisher, Loughborough, UK). The RNA was precipitated overnight at −80 °C using 0.3 M ammonium acetate, washed with 70% ethanol, resuspended in nuclease-free water and used for cDNA synthesis utilizing the Invitrogen RETROscript Reverse Transcription Kit (Thermo Fisher), following the manufacturer’s instructions, and a provided set of random primers.

Tg244440 was amplified from *T. gondii* cDNA with primers HDK1328 (forward; TTCATCAAGCTTATGAGTACAATCGAAGAGAGAGCC; *Hin*dIII) and HDK1329 (reverse; ATGATAGGATCCCTAGTAAGCGAGAGCGAGGTAC; *Bam*HI) using the high-fidelity Phusion DNA polymerase (New England Biolabs) and cloned into pHD1336 [64] after overnight digestion with *Hin*dIII and *Bam*HI. The final plasmid was then sequenced and used for transfection into *T. brucei*.

### 4.3. T. brucei Axenic Culture and Genetic Modification

*T. brucei* procyclic form (PCF) cells of TbNBT-KO, a clonal line derived from Lister strain 427, from which a locus of three nucleobase transporter in tandem repeat was deleted [32], were cultivated in SDM-79 medium (Life Technologies), supplemented with 7.5 μg/mL of hemin and 10% of Fetal Bovine Serum (FBS, Biosera, Kansas City, MO, USA) in non-vented plastic bottles at 27 °C. Cells in logarithmic growth were harvested by centrifugation, resuspended in Cytomix buffer and transfected with *Not*I-linearized plasmid pHD1336 containing the amplified Tg244440 gene from *T. gondii*. Transfectant cells were selected exactly as reported previously [32].

### 4.4. Uptake Assays

The transport of purines was measured using tritiated guanine (20 Ci/mmol; American Radiolabeled Chemicals) and hypoxanthine (12.8 Ci/mmol; Perkin-Elmer) diluted in assay buffer (AB; 33 mM HEPES, 98 mM NaCl, 4.6 mM KCl, 0.5 mM CaCl_2_, 0.07 mM MgSO_4_, 5.8 mM NaH_2_PO_4_, 0.03 mM MgCl_2_, 23 mM NaHCO_3_, 14 mM D-glucose, pH 7.3), in the presence or absence of potential transporter inhibitors at a predetermined concentration, as described previously for *Trypanosoma brucei* and *Leishmania* [65,66].

Briefly, PCF cells of *T. brucei* in logarithmic growth were harvested by centrifugation at 1000× *g* for 10 min, washed twice in AB and then resuspended in fresh buffer at a final density of 10^8^ cells/mL. Cell suspension in the amount of 100 μL was added to an equal volume of radiolabel solution for a predetermined time at room temperature, after which the reaction was stopped by the addition of 1 mL of a saturating concentration of ice-cold unlabeled substrate solution in AB (usually 1 mM hypoxanthine); this was followed by immediate centrifugation through an oil layer (7:1 (*v*/*v*) mix of di-n-butylphthalate and mineral oil (Sigma)). Immediately after finishing the assay, all tubes were flash-frozen in liquid nitrogen and the cell pellets were cut into scintillation vials and lysed with a solution of 2% SDS for one hour under agitation. Then, 3 mL of scintillation fluid (Scintilogic U, Lablogic) was added to each vial and left agitating overnight on a rocking platform. The next morning, the vials were vigorously shaken to ensure optimal mixing and the scintillation was measured in a 300SL (Hidex) scintillation counter. All assays were carried out in triplicate. All experimental data such as *K*_m_, *K*_i_ and *V*_max_ values are the average and SEM of at least three separate experiments, each in triplicate, unless otherwise indicated.

Background scintillation was measured in vials containing no cells or radiolabeled substrate, and counts associated with extracellular radiolabel were calculated from samples where cells were briefly incubated with the same concentration of radiolabel, but in the presence of saturating concentrations of non-radiolabeled substrate before centrifugation through oil. The average of the three control replicates was subtracted from each data point.

### 4.5. Calculation of Transport Kinetic Parameters

To determine the *K*_m_, cells were incubated with a low, non-saturating concentration of radiolabeled substrate and increasing concentrations of unlabeled substrate for 30 s, a time that falls very much within the linear phase of transport (see the Results section) during which the observed rate reflects the true initial rate of transport rather than intracellular accumulation of substrate, depletion of substrate from the assay buffer or transporter turnover. The resulting transport data was plotted to the Michaelis-Menten equation (*V*_0_ = *V*_max_ × [S]/(*K*_m_ + [S]), with [S] being the substrate concentration, using GraphPad Prism 8.0. The inhibition constants (*K*_i_) were calculated using the Cheng-Prusoff equation: *K*_i_ = IC_50_/(1 + ([S]/*K*_m_)) [67]. IC_50_ values were obtained from sigmoid dose-response plots with variable slope, using a constant permeant concentration several-fold below the *K*_m_ and a variable inhibitor concentration. The Gibbs free energy (ΔG^0^) of the inhibitor-transporter interaction was calculated using the equation ΔG^0^ = −RTln(*K*_i_), in which R is the gas constant and T is the absolute temperature of the reaction. It should be noted that these equations apply to competitive inhibitors, which is likely to be the case given (1) all inhibition curves presented Hill slopes consistently close to -1 and, most importantly, (2) no background for [^3^H]-guanine uptake (the radiolabeled probe used for transport inhibition assays) was seen in TbNBT-KO cells, evidencing that the heterologous expression of Tg244440 created a single guanine transport mechanism in these cells.

### 4.6. Synthesis of Nucleoside and Nucleobase Analogues

Nucleosides, nucleobases and nucleoside analogues were obtained from commercial sources where available, itemized in Table S1 of the Supplemental Information. A number of nucleoside analogues were synthesized as described previously (Table S2 of the Supplemental Information) or synthesized through new routes described in Supplemental File S2.

### 4.7. Phylogenetic Analysis

Amino acid sequences of transporters belonging to several purine and pyrimidine transporter families [12] were obtained from genomic data bases, aligned with Clustal Omega [68] and then subjected to a Maximum Likelihood analysis in Mega-X [69] using the Jones-Taylor-Thornton substitution model with 500 bootstraps. All sequences used for the phylogenetic tree, as well as the transporter families they belong to and the organisms they come from, are listed in Supplemental Information Table S4.

## Figures and Tables

**Table 1:** Increase in the rate of [^3^H]-guanine and [^3^H]-hypoxanthine uptake in TbNBT-KO cells transfected with the empty vector (EV) or Tg244440 (clone F5).

Substrate	[Permeant] (μM)	Rate (pmol(10^7^ Cells)^−1^s^−1^ ± SD)					
		EV	*P*_1_ (Non-Zero)	*P*_2_ (Non-Linear)	+Tg244440	*P*_1_ (Non-Zero)	*P*_2_ (Non-Linear)
Guanine	0.05	0.0.00059 ± 0.900026	0.11	0.90	0.037 ± 0.003	0.0012	>0.99
	25 *	0.0015 ± 0.0002	0.05	>0.99	0.00092 ± 0.00067	0.40	>0.99
Hypoxanthine	0.1	0.0066 ± 0.0007	0.012	>0.99	0.048 ± 0.0004	<0.0001	>0.99
	1000 *	0.00055	N/A	N/A	−0.01 ± 0.01	N/A	N/A

*P*_1_: significantly non-zero slope? F-test (Prism 9.2)*. P*_2_: significantly non-linear? Runs test (Prism 9.2). * High concentrations of unlabelled guanine or hypoxanthine used to saturate the studied transporter. N/A, not applicable.

**Table 2:** Tg244440 Michaelis-Menten constant (*K*_m_, guanine), inhibition constants (*K*_i_) and Gibbs Free Energy of interaction (ΔG^0^) for various purine analogues. The structure of each purine and purine analogue is shown in Supplemental Table S3.

	*K*_m_ or *K*_i_ (µM)	n	ΔG^0^ (kJ/mol)	δ(ΔG^0^) (kJ/mol)	Relative to
*Purine nucleobases*					
Guanine	1.25 ± 0.16	3	−33.7		
Hypoxanthine	2.26 ± 0.36	4	−32.2	1.5	Guanine
Xanthine	42.4 ± 4.2	3	−24.9	8.7	Guanine
Adenine	425 ± 80	8	−19.2	13.0	Hypoxanthine
6-Chloropurine	109 ± 8	4	−22.6	9.6	Hypoxanthine
6-Mercaptopurine	3.60 ± 0.80	3	−31.1	1.2	Hypoxanthine
6-Thioguanine	1.64 ± 0.25	3	−33.0	0.7	Guanine
1-deazahypoxanthine	194 ± 43	5	−21.2	11.0	Hypoxanthine
1-methylhypoxanthine	86.2 ± 7.8	4	−23.2	9.0	Hypoxanthine
2,6-diaminopurine	211 ± 3.5	3	−21.0	−1.7	Adenine
3-deazahypoxanthine	59.5 ± 7.2	4	−24.1	8.1	Hypoxanthine
3-methylxanthine	14.6 ± 3.1	4	−27.6	−2.7	Xanthine
7-deazahypoxanthine	318 ± 30	4	−20.0	12.3	Hypoxanthine
7-deazaguanine	49.2 ± 5.8	4	−24.6	9.1	Guanine
Allopurinol	185 ± 15	4	−21.3	10.9	Hypoxanthine
7-Br-allopurinol	65.7 ± 3.7	3	−23.9	−2.5	Allopurinol
Aminopurinol	223 ± 27	3	−20.8	−1.6	Adenine
8-azahypoxanthine	>1000	2	<−17.1	>15.1	Hypoxanthine
9-deazahypoxanthine	9.12 ± 0.66	3	−28.8	3.5	Hypoxanthine
9-deazaguanine	9.64 ± 1.59	4	−28.6	5.1	Guanine
9-deazaxanthine	48.2 ± 5.4	3	−24.6	0.3	Xanthine
9-Me,1-deazahypoxanthine	>1000	2	<−17.1	>15.1	Hypoxanthine
*Purine nucleosides*					
Guanosine	3.30 ± 0.30	7	−31.3	2.4	Guanine
Inosine	4.05 ± 1.37	4	−30.8	1.4	Hypoxanthine
Adenosine	68.18 ± 2.86	5	−23.8	−4.5	Adenine
2′-deoxyinosine	117 ± 12	4	−22.4	8.3	Inosine
3′-deoxyinosine	102 ± 15	3	−22.8	8.0	Inosine
2′,3′-dideoxyinosine	698 ± 52	3	−18.0	12.8	Inosine
5′-deoxyinosine	35.3 ± 3.0	3	−25.4	5.4	Inosine
2′-deoxyguanosine	281 ± 6	3	−20.3	11.0	Guanosine
3′-deoxyguanosine	196 ± 12	3	−21.2	10.1	Guanosine
2′-deoxyadenosine	83.9 ± 6.2	4	−23.3	0.5	Adenosine
3′-deoxyadenosine	71.9 ± 2.3	3	−23.6	0.1	Adenosine
5′-deoxyadenosine	85.2 ± 4.2	3	−23.2	0.6	Adenosine
3′-deoxy,7-deazaadenosine	797 ± 116	3	−17.7	6.1	Adenosine
Adenine arabinoside (Ara-A)	310 ± 14.0	3	−20.0	−3.8	Adenosine
1-deazainosine	524 ± 53	3	−18.7	12.1	Inosine
1-deazaadenosine	191 ± 10	3	−21.2	2.6	Adenosine
3-deazaadenosine	1220 ± 140	3	−16.6	7.2	Adenosine
Nebularine	491 ± 75	3	−18.9	4.9	Adenosine
6-thioinosine	7.3 ± 1.2	4	−29.3	1.4	Inosine
6-*O*-methyl,7-deaza,7-Cl-inosine	39.6 ± 2.8	3	−25.1	5.7	Inosine
6-*O*-ethyl,7-deaza,7-Cl-inosine	169 ± 10	3	−21.5	9.2	Inosine
7-deazainosine	6.32 ± 0.32	3	−29.7	1.1	Inosine
7-deaza-7-Chloroinosine	8.9 ± 1.3	3	−28.8	1.9	Inosine
7-deaza-7-Bromoinosine	7.8 ± 1.2	3	−29.2	1.6	Inosine
7-deaza-2′-deoxyinosine	52.3 ± 8.4	3	−24.4	−2.0	2′-Deoxyinosine
7-deaza-3′-deoxyinosine	70.8 ± 11.2	3	−23.7	−0.9	3′-Deoxyinosine
S-(4-nitrobenzyl)-6-thioinosine (NBMPR)	78 ± 6	3	−23.4	0.35	Adenosine

## Supplementary Materials

The following supporting information can be downloaded at: https://www.mdpi.com/article/10.3390/ijms23020710/s1.
