# Supplementary material for: A Toxoplasma gondii Oxopurine Transporter Binds Nucleobases and Nucleosides Using Different Binding Modes
Source: Int J Mol Sci. 2022 Jan 10;23(2):710. doi: 10.3390/ijms23020710 (PMC8776092; doi:10.3390/ijms23020710)
Supplement: Supplementary file 1 [file ijms-23-00710-s001.zip › Supplemental Figure S1.pdf]

Supplemental Figure S1. Multiple alignment of the Tg244440 amino acid sequence from 15 different strains. The single difference, at position 304 of strain MAS, is indicated with a red star. Source: ToxoDB.

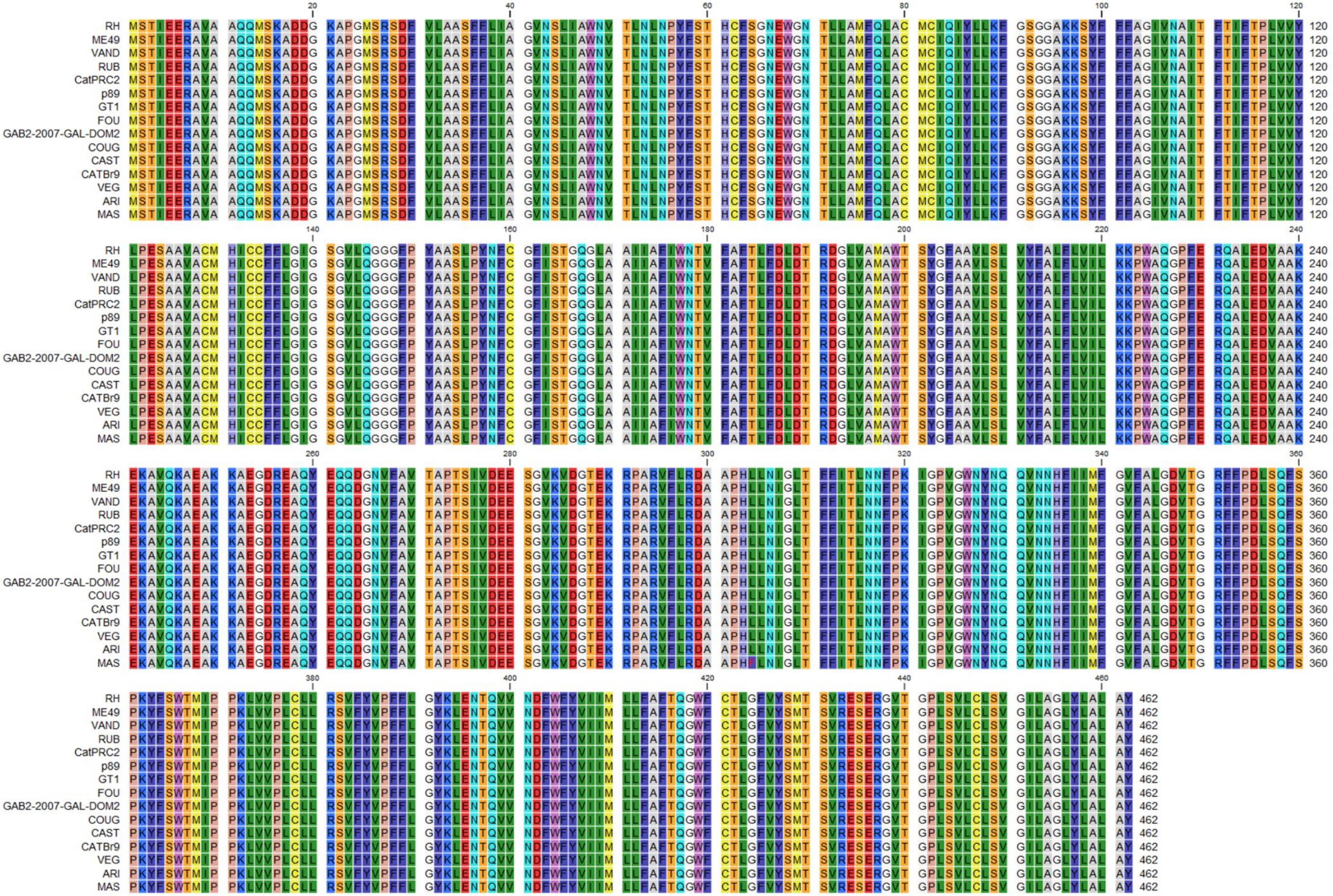
RH
ME49
VAND
RUB
CatPRC2
p89
GT1
FOU
GAB2-2007-GAL-DOM2
COUG
CAST
CATBr9
VEG
ARI
MAS
