# Supplementary material for: A Toxoplasma gondii Oxopurine Transporter Binds Nucleobases and Nucleosides Using Different Binding Modes
Source: Int J Mol Sci. 2022 Jan 10;23(2):710. doi: 10.3390/ijms23020710 (PMC8776092; doi:10.3390/ijms23020710)
Supplement: Supplementary file 1 [file ijms-23-00710-s001.zip › Supplemental File S1.pdf]

Supplemental File S1. Multiple alignments of T. gondii ENT transporters.

TGVEG_233230

```
CLUSTAL O(1.2.4) multiple sequence alignment


VEG                     MSGSKSGPAGLPVVAVKHPGVLSVANLDLSTPESVSRSVSPDEVVTMDSVAIDDASKAPG      60
ARI                     MSGSKSGPAGLPVVAVKHPGVLSVANLDLSTPESVSRSVSPDEVVTMDSVAIDDASKAPG      60
COUG                    MSGSKSGPAGLPVVAVKHPGVLSVANLDLSTPESVSRSVSPDEVVTMDSVAIDDASKAPG      60
ME49                    MSGSKSGPAGLPVVAVKHPGVLSVANLDLSTPESVSRSVSPDEVVTMDSVAIDDASKAPG      60
CatPRC2                 MSGSKSGPAGLPVVAVKHPGVLSVANLDLSTPESVSRSVSPDEVVTMDSVAIDDASKAPG      60
FOU                     MSGSKSGPAGLPVVAVKHPGVLSVANLDLSTPESVSRSVSPDEVVTMDSVAIDDASKAPG      60
GAB2-2007-GAL-DOM2      MSGSKSGPAGLPVVAVKHPGVLSVANLDLSTPESVSRSVSPDEVVTMDSVAIDDASKAPG      60
GT1                     MSGSKSGPAGLPVVAVKHPGVLSVANLDLSTPESVSRSVSPDEVVTMDSVAIDDASKAPG      60
RH                      MSGSKSGPAGLPVVAVKHPGVLSVANLDLSTPESVSRSVSPDEVVTMDSVAIDDASKAPG      60
CATBr9                  MSGSKSGPAGLPVVAVKHPGVLSVANLDLSTPESVSRSVSPDEVVTMDSVAIDDASKAPG      60
CAST                    MSGSKSGPAGLPVVAVKHPGVLSVANLDLSTPESVSRSVSPDEVVTMDSVAIDDASKAPG      60
p89                     MSGSKSGPAGLPVVAVKHPGVLSVANLDLSTPESVSRSVSPDEVVTMDSVAIDDASKAPG      60
MAS                     MSGSKSGPAGLPVVAVKHPGVLSVANLDLSTPESVSRSVSPDEVVTMDSVAIDDASKAPG      60
RUB                     MSGSKSGPAGLPVVAVKHPGVLSVANLDLSTPESVSRSVSPDEVVTMDSVAIDDASKAPG      60
VAND                    MSGSKSGPAGLPVVAVKHPGVLSVANLDLSTPESVSRSVSPDEVVTMDSVAIDDASKAPG      60
                        ************************************************************

VEG                     NYPEMSVPGSAGSAKTKFNSTLAYLTFLFVGANSLVNWAFVMQIIPFIAHSFLDNQDWNN      120
ARI                     NYPEMSVPGSAGSAKTKFNSTLAYLTFLFVGANSLVNWAFVMQIIPFIAHSFLDNQDWNN      120
COUG                    NYPEMSVPGSAGSAKTKFNSTLAYLTFLFVGANSLVNWAFVMQIIPFIAHSFLDNQDWNN      120
ME49                    NYPEMSVPGSAGSAKTKFNSTLAYLTFLFVGANSLVNWAFVMQIIPFIAHSFLDNQDWNN      120
CatPRC2                 NYPEMSVPGSAGSAKTKFNSTLAYLTFLFVGANSLVNWAFVMQIIPFIAHSFLDNQDWNN      120
FOU                     NYPEMSVPGSAGSAKTKFNSTLAYLTFLFVGANSLVNWAFVMQIIPFIAHSFLDNQDWNN      120
GAB2-2007-GAL-DOM2      NYPEMSVPGSAGSAKTKFNSTLAYLTFLFVGANSLVNWAFVMQIIPFIAHSFLDNQDWNN      120
GT1                     NYPEMSVPGSAGSAKTKFNSTLAYLTFLFVGANSLVNWAFVMQIIPFIAHSFLDNQDWNN      120
RH                      NYPEMSVPGSAGSAKTKFNSTLAYLTFLFVGANSLVNWAFVMQIIPFIAHSFLDNQDWNN      120
CATBr9                  NYPEMSVPGSAGSAKTKFNSTLAYLTFLFVGANSLVNWAFVMQIIPFIAHSFLDNQDWNN      120
CAST                    NYPEMSVPGSAGSAKTKFNSTLAYLTFLFVGANSLVNWAFVMQIIPFIAHSFLDNQDWNN      120
p89                     NYPEMSVPGSAGSAKTKFNSTLAYLTFLFVGANSLVNWAFVMQIIPFIAHSFLDNQDWNN      120
MAS                     NYPEMSVPGSAGSAKTKFNSTLAYLTFLFVGANSLVNWAFVMQIIPFIAHSFLDNQDWNN      120
RUB                     NYPEMSVPGSAGSAKTKFNSTLAYLTFLFVGANSLVNWAFVMQIIPFIAHSFLDNQDWNN      120
VAND                    NYPEMSVPGSAGSAKTKFNSTLAYLTFLFVGANSLVNWAFVMQIIPFIAHSFLDNQDWNN      120
                        ************************************************************

VEG                     TLLGSFQAIEVVVQLAMLELGSTHVNAVCLAGVINAIAGLLIAPLTLYTNETVSVWMLHL      180
ARI                     TLLGSFQAIEVVVQLAMLELGSTHVNAVCLAGVINAIAGLLIAPLTLYTNETVSVWMLHL      180
COUG                    TLLGSFQAIEVVVQLAMLELGSTHVNAVCLAGVINAIAGLLIAPLTLYTNETVSVWMLHL      180
ME49                    TLLGSFQAIEVVVQLAMLELGSTHVNAVCLAGVINAIAGLLIAPLTLYTNETVSVWMLHL      180
CatPRC2                 TLLGSFQAIEVVVQLAMLELGSTHVNAVCLAGVINAIAGLLIAPLTLYTNETVSVWMLHL      180
FOU                     TLLGSFQAIEVVVQLAMLELGSTHVNAVCLAGVINAIAGLLIAPLTLYTSETVSVWMLHL      180
GAB2-2007-GAL-DOM2      TLLGSFQAIEVVVQLAMLELGSTHVNAVCLAGVINAIAGLLIAPLTLYTSETVSVWMLHL      180
GT1                     TLLGSFQAIEVVVQLAMLELGSTHVNAVCLAGVINAIAGLLIAPLTLYTSETVSVWMLHL      180
RH                      TLLGSFQAIEVVVQLAMLELGSTHVNAVCLAGVINAIAGLLIAPLTLYTSETVSVWMLHL      180
CATBr9                  TLLGSFQAIEVVVQLAMLELGSTHVNAVCLAGVINAIAGLLIAPLTLYTSETVSVWMLHL      180
CAST                    TLLGSFQAIEVVVQLAMLELGSTHVNAVCLAGVINAIAGLLIAPLTLYTSETVSVWMLHL      180
p89                     TLLGSFQAIEVVVQLAMLELGSTHVNAVCLAGVINAIAGLLIAPLTLYTSETVSVWMLHL      180
MAS                     TLLGSFQAIEVVVQLAMLELGSTHVNAVCLAGVINAIAGLLIAPLTLYTSETVSVWMLHL      180
RUB                     TLLGSFQAIEVVVQLAMLELGSTHVNAVCLAGVINAIAGLLIAPLTLYTSETVSVWMLHL      180
VAND                    TLLGSFQAIEVVVQLAMLELGSTHVNAVCLAGVINAIAGLLIAPLTLYTSETVSVWMLHL      180
                        *************************************************.**********

VEG                     ICLVLGACSGIYQGSGYAIASMMPRNFVSAVSTGQGLAGLFVFAVVVGVSFAVFDVDTPA      240
ARI                     ICLVLGACSGIYQGSGYAIASMMPRNFVSAVSTGQGLAGLFVFAVVVGVSFAVFDVDTPA      240
COUG                    ICLVLGACSGIYQGSGYAIASMMPRNFVSAVSTGQGLAGLFVFAVVVGVSFAVFDVDTPA      240
ME49                    ICLVLGACSGIYQGSGYAIASMMPRNFVSAVSTGQGLAGLFVFAVVVGVSFAVFDVDTPA      240
CatPRC2                 ICLVLGACSGIYQGSGYAIASMMPRNFVSAVSTGQGLAGLFVFAVVVGVSFAVFDVDTPA      240
FOU                     ICLVLGACSGIYQGSGYAIASMMPRNFVSAVSTGQGLAGLFVFAVVVGVSFAVFDVDTPA      240
GAB2-2007-GAL-DOM2      ICLVLGACSGIYQGSGYAIASMMPRNFVSAVSTGQGLAGLFVFAVVVGVSFAVFDVDTPA      240
GT1                     ICLVLGACSGIYQGSGYAIASMMPRNFVSAVSTGQGLAGLFVFAVVVGVSFAVFDVDTPA      240
RH                      ICLVLGACSGIYQGSGYAIASMMPRNFVSAVSTGQGLAGLFVFAVVVGVSFAVFDVDTPA      240
CATBr9                  ICLVLGACSGIYQGSGYAIASMMPRNFVSAVSTGQGLAGLFVFAVVVGVSFAVFDVDTPA      240
CAST                    ICLVLGACSGIYQGSGYAIASMMPRNFVSAVSTGQGLAGLFVFAVVVGVSFAVFDVDTPA      240
p89                     ICLVLGACSGIYQGSGYAIASMMPRNFVSAVSTGQGLAGLFVFAVVVGVSFAVFDVDTPA      240
MAS                     ICLVLGACSGIYQGSGYAIASMMPRNFVSAVSTGQGLAGLFVFAVVVGVSFAVFDVDTPA      240
RUB                     ICLVLGACSGIYQGSGYAIASMMPRNFVSAVSTGQGLAGLFVFAVVVGVSFAVFDVDTPA      240
VAND                    ICLVLGACSGIYQGSGYAIASMMPRNFVSAVSTGQGLAGLFVFAVVVGVSFAVFDVDTPA      240
                        ************************************************************
```

```
VEG                  GTEGMVWTGFSISAVLSVVCAVVFFLVMRQSWAVACLTRVREERALKRAAAGVAKTKKEE    300
ARI                  GTEGMVWTGFSISAVLSVVCAVVFFLVMRQSWAVACLTRVREERALKRAAAGVAKTKKEE    300
COUG                 GTEGMVWTGFSISAVLSVVCAVVFFLVMRQSWAVACLTRVREERALKRAAAGVAKTKKEE    300
ME49                 GTEGMVWTGFSISAVLSVVCAVVFFLVMRQSWAVACLTRVREERALKRAAAGVAKTKKEE    300
CatPRC2              GTEGMVWTGFSISAVLSVVCAVVFFLVMRQSWAVACLTRVREERALKRAAAGVAKTKKEE    300
FOU                  GTEGMVWTGFSISAVLSVVCAVVFFLVMRQSWAVACLTRVREERALKRAAARVAKTKKEE    300
GAB2-2007-GAL-DOM2   GTEGMVWTGFSISAVLSVVCAVVFFLVMRQSWAVACLTRVREERALKRAAARVAKTKKEE    300
GT1                  GTEGMVWTGFSISAVLSVVCAVVFFLVMRQSWAVACLTRVREERALKRAAARVAKTKKEE    300
RH                   GTEGMVWTGFSISAVLSVVCAVVFFLVMRQSWAVACLTRVREERALKRAAARVAKTKKEE    300
CATBr9               GTEGMVWTGFSISAVLSVVCAVVFFLVMRQSWAVACLTRVREERALKRAAARVAKTKKEE    300
CAST                 GTEGMVWTGFSISAVLSVVCAVVFFLVMRQSWAVACLTRVREERALKRAAAGVAKTKKEE    300
p89                  GTEGMVWTGFSISAVLSVVCAVVFFLVMRQSWAVACLTRVREERALKRAAAGVAKTKKEE    300
MAS                  GTEGMVWTGFSISAVLSVVCAVVFFLVMRQSWAVACLTRVREERALKRAAAGVAKTKKEE    300
RUB                  GTEGMVWTGFSISAVLSVVCAVVFFLVMRQSWAVACLTRVREERALKRAAAGVAKTKKEE    300
VAND                 GTEGMVWTGFSISAVLSVVCAVVFFLVMRQSWAVACLTRVREERALKRAAAGVAKTKKEE    300
                     *************************************************** ********

VEG                  PRKDFADGRSVGNEEPFRGPSSQMTVVTMLTPEELSKEVEIDDGRRAMTQNETQKSQVGV    360
ARI                  PRKDFADGRSVGNEEPFRGPSSQMTVVTMLTPEELSKEVEIDDGRRAMTQNETQKSQVGV    360
COUG                 PRKDFADGRSVGNEEPFRGPSSQMTVVTMLTPEELSKEVEIDDGRRAMTQNETQKSQVGV    360
ME49                 PRKDFADGRSVGNEEPFRGPSSQMTVVTMLTPEELSKEVEIDDGRRAMTQNETQKSQVGV    360
CatPRC2              PRKDFADGRSVGNEEPFRGPSSQMTVVTMLTPEELSKEVEIDDGRRAMTQNETQKSQVGV    360
FOU                  PRKDFADGRSVGNEEPFRGPSSQMTVVTMLTPEELSKEVEIDNGRRAMTQNETQKSQVGV    360
GAB2-2007-GAL-DOM2   PRKDFADGRSVGNEEPFRGPSSQMTVVTMLTPEELSKEVEIDNGRRAMTQNETQKSQVGV    360
GT1                  PRKDFADGRSVGNEEPFRGPSSQMTVVTMLTPEELSKEVEIDNGRRAMTQNETQKSQVGV    360
RH                   PRKDFADGRSVGNEEPFRGPSSQMTVVTMLTPEELSKEVEIDNGRRAMTQNETQKSQVGV    360
CATBr9               PRKDFADGRSVGNEEPFRGPSSQMTVVTMLTPEELSKEVEIDNGRRAMTQNETQKSQVGV    360
CAST                 PRKDFADGRSVGNEELFRGPSSQMTVVTMLTPEELSKEVEIDNGRRAMTQNETQKSQVGV    360
p89                  PRKDFADGRSVGNEELFRGPSSQMTVVTMLTPEELSKEVEIDNGRRAMTQNETQKSQVGV    360
MAS                  PRKDFADGRSVGNEEPFRGPSSQMTVVTMLTPEELSKEVEIDNGRRAMTQNETQKSQVGV    360
RUB                  PRKDFADGRSVGNEEPFRGPSSQMTVVTMLTPEELSKEVEIDNGRRAMTQNETQKSQVGV    360
VAND                 PRKDFADGRSVGNEEPFRGPSSQMTVVTMLTPEELSKEVEIDNGRRAMTQNETQKSQVGV    360
                     *************** **************************:*****************

VEG                  RPWPAVLKDASPWLFMLIFHMFTSFHLFPKVGPLSWNYVDPPKNHLVILFGIFYVTEFVG    420
ARI                  RPWPAVLKDASPWLFMLIFHMFTSFHLFPKVGPLSWNYVDPPKNHLVILFGIFYVTEFVG    420
COUG                 RPWPAVLKDASPWLFMLIFHMFTSFHLFPKVGPLSWNYVDPPKNHLVILFGIFYVTEFVG    420
ME49                 RPWPAVLKDASPWLFMLIFHMFTSFHLFPKVGPLSWNYVDPPKNHLVILFGIFYVTEFVG    420
CatPRC2              RPWPAVLKDASPWLFMLIFHMFTSFHLFPKVGPLSWNYVDPPKNHLVILFGIFYVTEFVG    420
FOU                  RPWPAVLKDASPWLFMLIFHMFTSFHLFPKVGPLSWNYVDPPKNHLVILFGIFYVTEFVG    420
GAB2-2007-GAL-DOM2   RPWPAVLKDASPWLFMLIFHMFTSFHLFPKVGPLSWNYVDPPKNHLVILFGIFYVTEFVG    420
GT1                  RPWPAVLKDASPWLFMLIFHMFTSFHLFPKVGPLSWNYVDPPKNHLVILFGIFYVTEFVG    420
RH                   RPWPAVLKDASPWLFMLIFHMFTSFHLFPKVGPLSWNYVDPPKNHLVILFGIFYVTEFVG    420
CATBr9               RPWPAVLKDASPWLFMLIFHMFTSFHLFPKVGPLSWNYVDPPKNHLVILFGIFYVTEFVG    420
CAST                 RPWPAVLKDASPWLFMLIFHMFTSFHLFPKVGPLSWNYVDPPKNHLVILFGIFYVTEFVG    420
p89                  RPWPAVLKDASPWLFMLIFHMFTSFHLFPKVGPLSWNYVDPPKNHLVILFGIFYVTEFVG    420
MAS                  RPWPAVLKDASPWLFMLIFHMFTSFHLFPKVGPLSWNYVDPPKNHLVILFGIFYVTEFVG    420
RUB                  RPWPAVLKDASPWLFMLIFHMFTSFHLFPKVGPLSWNYVDPPKNHLVILFGIFYVTEFVG    420
VAND                 RPWPAVLKDASPWLFMLIFHMFTSFHLFPKVGPLSWNYVDPPKNHLVILFGIFYVTEFVG    420
                     ************************************************************

VEG                  RSLPDLCTIRGLGFLHLSRRMFVVAEFARLLFFLPFVLGYAVSNTPFLNNFYWCILIAS    480
ARI                  RSLPDLCTIRGLGFLHLSRRMFVVAEFARLLFFLPFVLGYAVSNTPFLNNFYWCILIAS    480
COUG                 RSLPDLCTIRGLGFLHLSRRMFVVAEFARLLFFLPFVLGYAVSNTPFLNNFYWCILIAS    480
ME49                 RSLPDLCTIRGLGFLHLSRRMFVVAEFARLLFFLPFVLGYAVSNTPFLNNFYWCILIAS    480
CatPRC2              RSLPDLCTIRGLGFLHLSRRMFVVAEFARLLFFLPFVLGYAVSNTPFLNNFYWCILIAS    480
FOU                  RSLPDLCTIRGLGFLHLSRRMFVVAEFARLLFFLPFVLGYAVSNTPFLNNFYWCILIAS    480
GAB2-2007-GAL-DOM2   RSLPDLCTIRGLGFLHLSRRMFVVAEFARLLFFLPFVLGYAVSNTPFLNNFYWCILIAS    480
GT1                  RSLPDLCTIRGLGFLHLSRRMFVVAEFARLLFFLPFVLGYAVSNTPFLNNFYWCILIAS    480
RH                   RSLPDLCTIRGLGFLHLSRRMFVVAEFARLLFFLPFVLGYAVSNTPFLNNFYWCILIAS    480
CATBr9               RSLPDLCTIRGLGFLHLSRRMFVVAEFARLLFFLPFVLGYAVSNTPFLNNFYWCILIAS    480
CAST                 RSLPDLCTIRGLGFLHLSRRMFVVAEFARLLFFLPFVLGYAVSNTPFLNNFYWCILIAS    480
p89                  RSLPDLCTIRGLGFLHLSRRMFVVAEFARLLFFLPFVLGYAVSNTPFLNNFYWCILIAS    480
MAS                  RSLPDLCTIRGLGFLHLSRRMFVVAEFARLLFFLPFVLGYAVSNTPFLNNFYWCILIAS    480
RUB                  RSLPDLCTIRGLGFLHLSRRMFVVAEFARLLFFLPFVLGYAVSNTPFLNNFYWCILIAS    480
VAND                 RSLPDLCTIRGLGFLHLSRRMFVVAEFARLLFFLPFVLGYAVSNTPFLNNFYWCILIAS    480
                     ************************************************************

VEG                  LSLTQGWLGTLAFYYAVNSVESPAERELTGPMAAIASPFGCVIGLYTAAPY    531
ARI                  LSLTQGWLGTLAFYYAVNSVESPAERELTGPMAAIASPFGCVIGLYTAAPY    531
COUG                 LSLTQGWLGTLAFYYAVNSVESPAERELTGPMAAIASPFGCVIGLYTAAPY    531
ME49                 LSLTQGWLGTLAFYYAVNSVESPAERELTGPMAAIASPFGCVIGLYTAAPY    531
CatPRC2              LSLTQGWLGTLAFYYAVNSVESPAERELTGPMAAIASPFGCVIGLYTAAPY    531
FOU                  LSLTQGWLGTLAFYYAVNSVESPAERELTGPMAAIASPFGCVIGLYTAAPY    531
GAB2-2007-GAL-DOM2   LSLTQGWLGTLAFYYAVNSVESPAERELTGPMAAIASPFGCVIGLYTAAPY    531
GT1                  LSLTQGWLGTLAFYYAVNSVESPAERELTGPMAAIASPFGCVIGLYTAAPY    531
```

```
RH                        LSLTQGWLGTLAFYYAVNSVESPAERELTGPMAAIASPFGCVIGLYTAAPY        531
CATBr9                    LSLTQGWLGTLAFYYAVNSVESPAERELTGPMAAIASPFGCVIGLYTAAPY        531
CAST                      LSLTQGWLGTLAFYYAVNSVESPAERELTGPMAAIASPFGCVIGLYTAAPY        531
p89                       LSLTQGWLGTLAFYYAVNSVESPAERELTGPMAAIASPFGCVIGLYTAAPY        531
MAS                       LSLTQGWLGTLAFYYAVNSVESPAERELTGPMAAIASPFGCVIGLYTAAPY        531
RUB                       LSLTQGWLGTLAFYYAVNSVESPAERELTGPMAAIASPFGCVIGLYTAAPY        531
VAND                      LSLTQGWLGTLAFYYAVNSVESPAERELTGPMAAIASPFGCVIGLYTAAPY        531
                          ***************************************************
```

Sequences for alignment of TGVEG_233230

>VEG
MSGSKSGPAGLPVVAVKHPGVLSVANLDLSTPESVSRSVSPDEVVTMDSVAIDDASKAPG
NYPEMSVPGSAGSAKTKFNSTLAYLTFLFVGANSLVNWAFVMQIIPFIAHSFLDNQDWNN
TLLGSFQAIEVVVQLAMLELGSTHVNAVCLAGVINAIAGLLIAPLTLYTNETVSVWMLHL
ICLVLGACSGIYQGSGYAIASMMPRNFVSAVSTGQGLAGLFVFAVVVGVSFAVFDVDTPA
GTEGMVWTGFSISAVLSVVCAVVFFLVMRQSWAVACLTRVREERALKRAAAGVAKTKKEE
PRKDFADGRSVGNEEPFRGPSSQMTVVTMLTPEELSKEVEIDDGRRAMTQNETQKSQVGV
RPWPAVLKDASPWLFMLIFHMFTSFHLFPKVGPLSWNYVDPPKNHLVILFGIFYVTEFVG
RSLPDLCTIRGLGFLHLSRRMFVVAEFARLLFFLPFVLGYAVSNTPFLNNFYWYCILIAS
LSLTQGWLGTLAFYYAVNSVESPAERELTGPMAAIASPFGCVIGLYTAAPY

>ARI
MSGSKSGPAGLPVVAVKHPGVLSVANLDLSTPESVSRSVSPDEVVTMDSVAIDDASKAPG
NYPEMSVPGSAGSAKTKFNSTLAYLTFLFVGANSLVNWAFVMQIIPFIAHSFLDNQDWNN
TLLGSFQAIEVVVQLAMLELGSTHVNAVCLAGVINAIAGLLIAPLTLYTNETVSVWMLHL
ICLVLGACSGIYQGSGYAIASMMPRNFVSAVSTGQGLAGLFVFAVVVGVSFAVFDVDTPA
GTEGMVWTGFSISAVLSVVCAVVFFLVMRQSWAVACLTRVREERALKRAAAGVAKTKKEE
PRKDFADGRSVGNEEPFRGPSSQMTVVTMLTPEELSKEVEIDDGRRAMTQNETQKSQVGV
RPWPAVLKDASPWLFMLIFHMFTSFHLFPKVGPLSWNYVDPPKNHLVILFGIFYVTEFVG
RSLPDLCTIRGLGFLHLSRRMFVVAEFARLLFFLPFVLGYAVSNTPFLNNFYWYCILIAS
LSLTQGWLGTLAFYYAVNSVESPAERELTGPMAAIASPFGCVIGLYTAAPY

>CAST
MSGSKSGPAGLPVVAVKHPGVLSVANLDLSTPESVSRSVSPDEVVTMDSVAIDDASKAPG
NYPEMSVPGSAGSAKTKFNSTLAYLTFLFVGANSLVNWAFVMQIIPFIAHSFLDNQDWNN
TLLGSFQAIEVVVQLAMLELGSTHVNAVCLAGVINAIAGLLIAPLTLYTSETVSVWMLHL
ICLVLGACSGIYQGSGYAIASMMPRNFVSAVSTGQGLAGLFVFAVVVGVSFAVFDVDTPA
GTEGMVWTGFSISAVLSVVCAVVFFLVMRQSWAVACLTRVREERALKRAAAGVAKTKKEE
PRKDFADGRSVGNEELFRGPSSQMTVVTMLTPEELSKEVEIDNGRRAMTQNETQKSQVGV
RPWPAVLKDASPWLFMLIFHMFTSFHLFPKVGPLSWNYVDPPKNHLVILFGIFYVTEFVG
RSLPDLCTIRGLGFLHLSRRMFVVAEFARLLFFLPFVLGYAVSNTPFLNNFYWYCILIAS
LSLTQGWLGTLAFYYAVNSVESPAERELTGPMAAIASPFGCVIGLYTAAPY

>COUG
MSGSKSGPAGLPVVAVKHPGVLSVANLDLSTPESVSRSVSPDEVVTMDSVAIDDASKAPG
NYPEMSVPGSAGSAKTKFNSTLAYLTFLFVGANSLVNWAFVMQIIPFIAHSFLDNQDWNN
TLLGSFQAIEVVVQLAMLELGSTHVNAVCLAGVINAIAGLLIAPLTLYTNETVSVWMLHL
ICLVLGACSGIYQGSGYAIASMMPRNFVSAVSTGQGLAGLFVFAVVVGVSFAVFDVDTPA
GTEGMVWTGFSISAVLSVVCAVVFFLVMRQSWAVACLTRVREERALKRAAAGVAKTKKEE
PRKDFADGRSVGNEEPFRGPSSQMTVVTMLTPEELSKEVEIDDGRRAMTQNETQKSQVGV
RPWPAVLKDASPWLFMLIFHMFTSFHLFPKVGPLSWNYVDPPKNHLVILFGIFYVTEFVG
RSLPDLCTIRGLGFLHLSRRMFVVAEFARLLFFLPFVLGYAVSNTPFLNNFYWYCILIAS
LSLTQGWLGTLAFYYAVNSVESPAERELTGPMAAIASPFGCVIGLYTAAPY

>FOU
MSGSKSGPAGLPVVAVKHPGVLSVANLDLSTPESVSRSVSPDEVVTMDSVAIDDASKAPG
NYPEMSVPGSAGSAKTKFNSTLAYLTFLFVGANSLVNWAFVMQIIPFIAHSFLDNQDWNN
TLLGSFQAIEVVVQLAMLELGSTHVNAVCLAGVINAIAGLLIAPLTLYTSETVSVWMLHL
ICLVLGACSGIYQGSGYAIASMMPRNFVSAVSTGQGLAGLFVFAVVVGVSFAVFDVDTPA
GTEGMVWTGFSISAVLSVVCAVVFFLVMRQSWAVACLTRVREERALKRAAARVAKTKKEE
PRKDFADGRSVGNEEPFRGPSSQMTVVTMLTPEELSKEVEIDNGRRAMTQNETQKSQVGV
RPWPAVLKDASPWLFMLIFHMFTSFHLFPKVGPLSWNYVDPPKNHLVILFGIFYVTEFVG
RSLPDLCTIRGLGFLHLSRRMFVVAEFARLLFFLPFVLGYAVSNTPFLNNFYWYCILIAS
LSLTQGWLGTLAFYYAVNSVESPAERELTGPMAAIASPFGCVIGLYTAAPY

>GAB2-2007-GAL-DOM2
MSGSKSGPAGLPVVAVKHPGVLSVANLDLSTPESVSRSVSPDEVVTMDSVAIDDASKAPG
NYPEMSVPGSAGSAKTKFNSTLAYLTFLFVGANSLVNWAFVMQIIPFIAHSFLDNQDWNN
TLLGSFQAIEVVVQLAMLELGSTHVNAVCLAGVINAIAGLLIAPLTLYTSETVSVWMLHL
ICLVLGACSGIYQGSGYAIASMMPRNFVSAVSTGQGLAGLFVFAVVVGVSFAVFDVDTPA
GTEGMVWTGFSISAVLSVVCAVVFFLVMRQSWAVACLTRVREERALKRAAARVAKTKKEE
PRKDFADGRSVGNEEPFRGPSSQMTVVTMLTPEELSKEVEIDNGRRAMTQNETQKSQVGV
RPWPAVLKDASPWLFMLIFHMFTSFHLFPKVGPLSWNYVDPPKNHLVILFGIFYVTEFVG
RSLPDLCTIRGLGFLHLSRRMFVVAEFARLLFFLPFVLGYAVSNTPFLNNFYWYCILIAS
LSLTQGWLGTLAFYYAVNSVESPAERELTGPMAAIASPFGCVIGLYTAAPY

>GT1
MSGSKSGPAGLPVVAVKHPGVLSVANLDLSTPESVSRSVSPDEVVTMDSVAIDDASKAPG
NYPEMSVPGSAGSAKTKFNSTLAYLTFLFVGANSLVNWAFVMQIIPFIAHSFLDNQDWNN
TLLGSFQAIEVVVQLAMLELGSTHVNAVCLAGVINAIAGLLIAPLTLYTSETVSVWMLHL
ICLVLGACSGIYQGSGYAIASMMPRNFVSAVSTGQGLAGLFVFAVVVGVSFAVFDVDTPA
GTEGMVWTGFSISAVLSVVCAVVFFLVMRQSWAVACLTRVREERALKRAAARVAKTKKEE
PRKDFADGRSVGNEEPFRGPSSQMTVVTMLTPEELSKEVEIDNGRRAMTQNETQKSQVGV
RPWPAVLKDASPWLFMLIFHMFTSFHLFPKVGPLSWNYVDPPKNHLVILFGIFYVTEFVG
RSLPDLCTIRGLGFLHLSRRMFVVAEFARLLFFLPFVLGYAVSNTPFLNNFYWYCILIAS

LSLTQGWLGTLAFYYAVNSVESPAERELTGPMAAIASPFGCVIGLYTAAPY

>MAS
MSGSKSGPAGLPVVAVKHPGVLSVANLDLSTPESVSRSVSPDEVVTMDSVAIDDASKAPG
NYPEMSVPGSAGSAKTKFNSTLAYLTFLFVGANSLVNWAFVMQIIPFIAHSFLDNQDWNN
TLLGSFQAIEVVVQLAMLELGSTHVNAVCLAGVINAIAGLLIAPLTLYTSETVSVWMLHL
ICLVLGACSGIYQGSGYAIASMMPRNFVSAVSTGQGLAGLFVFAVVVGVSFAVFDVDTPA
GTEGMVWTGFSISAVLSVVCAVVFFLVMRQSWAVACLTRVREERALKRAAAGVAKTKKEE
PRKDFADGRSVGNEEPFRGPSSQMTVVTMLTPEELSKEVEIDNGRRAMTQNETQKSQVGV
RPWPAVLKDASPWLFMLIFHMFTSFHLFPKVGPLSWNYVDPPKNHLVILFGIFYVTEFVG
RSLPDLCTIRGLGFLHLSRRMFVVAEFARLLFFLPFVLGYAVSNTPFLNNFYWYCILIAS
LSLTQGWLGTLAFYYAVNSVESPAERELTGPMAAIASPFGCVIGLYTAAPY

>ME49
MSGSKSGPAGLPVVAVKHPGVLSVANLDLSTPESVSRSVSPDEVVTMDSVAIDDASKAPG
NYPEMSVPGSAGSAKTKFNSTLAYLTFLFVGANSLVNWAFVMQIIPFIAHSFLDNQDWNN
TLLGSFQAIEVVVQLAMLELGSTHVNAVCLAGVINAIAGLLIAPLTLYTNETVSVWMLHL
ICLVLGACSGIYQGSGYAIASMMPRNFVSAVSTGQGLAGLFVFAVVVGVSFAVFDVDTPA
GTEGMVWTGFSISAVLSVVCAVVFFLVMRQSWAVACLTRVREERALKRAAAGVAKTKKEE
PRKDFADGRSVGNEEPFRGPSSQMTVVTMLTPEELSKEVEIDDGRRAMTQNETQKSQVGV
RPWPAVLKDASPWLFMLIFHMFTSFHLFPKVGPLSWNYVDPPKNHLVILFGIFYVTEFVG
RSLPDLCTIRGLGFLHLSRRMFVVAEFARLLFFLPFVLGYAVSNTPFLNNFYWYCILIAS
LSLTQGWLGTLAFYYAVNSVESPAERELTGPMAAIASPFGCVIGLYTAAPY

>RH
MSGSKSGPAGLPVVAVKHPGVLSVANLDLSTPESVSRSVSPDEVVTMDSVAIDDASKAPG
NYPEMSVPGSAGSAKTKFNSTLAYLTFLFVGANSLVNWAFVMQIIPFIAHSFLDNQDWNN
TLLGSFQAIEVVVQLAMLELGSTHVNAVCLAGVINAIAGLLIAPLTLYTSETVSVWMLHL
ICLVLGACSGIYQGSGYAIASMMPRNFVSAVSTGQGLAGLFVFAVVVGVSFAVFDVDTPA
GTEGMVWTGFSISAVLSVVCAVVFFLVMRQSWAVACLTRVREERALKRAAARVAKTKKEE
PRKDFADGRSVGNEEPFRGPSSQMTVVTMLTPEELSKEVEIDNGRRAMTQNETQKSQVGV
RPWPAVLKDASPWLFMLIFHMFTSFHLFPKVGPLSWNYVDPPKNHLVILFGIFYVTEFVG
RSLPDLCTIRGLGFLHLSRRMFVVAEFARLLFFLPFVLGYAVSNTPFLNNFYWYCILIAS
LSLTQGWLGTLAFYYAVNSVESPAERELTGPMAAIASPFGCVIGLYTAAPY

>RUB
MSGSKSGPAGLPVVAVKHPGVLSVANLDLSTPESVSRSVSPDEVVTMDSVAIDDASKAPG
NYPEMSVPGSAGSAKTKFNSTLAYLTFLFVGANSLVNWAFVMQIIPFIAHSFLDNQDWNN
TLLGSFQAIEVVVQLAMLELGSTHVNAVCLAGVINAIAGLLIAPLTLYTSETVSVWMLHL
ICLVLGACSGIYQGSGYAIASMMPRNFVSAVSTGQGLAGLFVFAVVVGVSFAVFDVDTPA
GTEGMVWTGFSISAVLSVVCAVVFFLVMRQSWAVACLTRVREERALKRAAAGVAKTKKEE
PRKDFADGRSVGNEEPFRGPSSQMTVVTMLTPEELSKEVEIDNGRRAMTQNETQKSQVGV
RPWPAVLKDASPWLFMLIFHMFTSFHLFPKVGPLSWNYVDPPKNHLVILFGIFYVTEFVG
RSLPDLCTIRGLGFLHLSRRMFVVAEFARLLFFLPFVLGYAVSNTPFLNNFYWYCILIAS
LSLTQGWLGTLAFYYAVNSVESPAERELTGPMAAIASPFGCVIGLYTAAPY

>CATBr9
MSGSKSGPAGLPVVAVKHPGVLSVANLDLSTPESVSRSVSPDEVVTMDSVAIDDASKAPG
NYPEMSVPGSAGSAKTKFNSTLAYLTFLFVGANSLVNWAFVMQIIPFIAHSFLDNQDWNN
TLLGSFQAIEVVVQLAMLELGSTHVNAVCLAGVINAIAGLLIAPLTLYTSETVSVWMLHL
ICLVLGACSGIYQGSGYAIASMMPRNFVSAVSTGQGLAGLFVFAVVVGVSFAVFDVDTPA
GTEGMVWTGFSISAVLSVVCAVVFFLVMRQSWAVACLTRVREERALKRAAARVAKTKKEE
PRKDFADGRSVGNEEPFRGPSSQMTVVTMLTPEELSKEVEIDNGRRAMTQNETQKSQVGV
RPWPAVLKDASPWLFMLIFHMFTSFHLFPKVGPLSWNYVDPPKNHLVILFGIFYVTEFVG
RSLPDLCTIRGLGFLHLSRRMFVVAEFARLLFFLPFVLGYAVSNTPFLNNFYWYCILIAS
LSLTQGWLGTLAFYYAVNSVESPAERELTGPMAAIASPFGCVIGLYTAAPY

>CatPRC2
MSGSKSGPAGLPVVAVKHPGVLSVANLDLSTPESVSRSVSPDEVVTMDSVAIDDASKAPG
NYPEMSVPGSAGSAKTKFNSTLAYLTFLFVGANSLVNWAFVMQIIPFIAHSFLDNQDWNN
TLLGSFQAIEVVVQLAMLELGSTHVNAVCLAGVINAIAGLLIAPLTLYTNETVSVWMLHL
ICLVLGACSGIYQGSGYAIASMMPRNFVSAVSTGQGLAGLFVFAVVVGVSFAVFDVDTPA
GTEGMVWTGFSISAVLSVVCAVVFFLVMRQSWAVACLTRVREERALKRAAAGVAKTKKEE
PRKDFADGRSVGNEEPFRGPSSQMTVVTMLTPEELSKEVEIDDGRRAMTQNETQKSQVGV
RPWPAVLKDASPWLFMLIFHMFTSFHLFPKVGPLSWNYVDPPKNHLVILFGIFYVTEFVG
RSLPDLCTIRGLGFLHLSRRMFVVAEFARLLFFLPFVLGYAVSNTPFLNNFYWYCILIAS
LSLTQGWLGTLAFYYAVNSVESPAERELTGPMAAIASPFGCVIGLYTAAPY

>VAND
MSGSKSGPAGLPVVAVKHPGVLSVANLDLSTPESVSRSVSPDEVVTMDSVAIDDASKAPG
NYPEMSVPGSAGSAKTKFNSTLAYLTFLFVGANSLVNWAFVMQIIPFIAHSFLDNQDWNN
TLLGSFQAIEVVVQLAMLELGSTHVNAVCLAGVINAIAGLLIAPLTLYTSETVSVWMLHL
ICLVLGACSGIYQGSGYAIASMMPRNFVSAVSTGQGLAGLFVFAVVVGVSFAVFDVDTPA
GTEGMVWTGFSISAVLSVVCAVVFFLVMRQSWAVACLTRVREERALKRAAAGVAKTKKEE
PRKDFADGRSVGNEEPFRGPSSQMTVVTMLTPEELSKEVEIDNGRRAMTQNETQKSQVGV
RPWPAVLKDASPWLFMLIFHMFTSFHLFPKVGPLSWNYVDPPKNHLVILFGIFYVTEFVG
RSLPDLCTIRGLGFLHLSRRMFVVAEFARLLFFLPFVLGYAVSNTPFLNNFYWYCILIAS

```
LSLTQGWLGTLAFYYAVNSVESPAERELTGPMAAIASPFGCVIGLYTAAPY

>p89
MSGSKSGPAGLPVVAVKHPGVLSVANLDLSTPESVSRSVSPDEVVTMDSVAIDDASKAPG
NYPEMSVPGSAGSAKTKFNSTLAYLTFLFVGANSLVNWAFVMQIIPFIAHSFLDNQDWNN
TLLGSFQAIEVVVQLAMLELGSTHVNAVCLAGVINAIAGLLIAPLTLYTSETVSVWMLHL
ICLVLGACSGIYQGSGYAIASMMPRNFVSAVSTGQGLAGLFVFAVVVGVSFAVFDVDTPA
GTEGMVWTGFSISAVLSVVCAVVFFLVMRQSWAVACLTRVREERALKRAAAGVAKTKKEE
PRKDFADGRSVGNEELFRGPSSQMTVVTMLTPEELSKEVEIDNGRRAMTQNETQKSQVGV
RPWPAVLKDASPWLFMLIFHMFTSFHLFPKVGPLSWNYVDPPKNHLVILFGIFYVTEFVG
RSLPDLCTIRGLGFLHLSRRMFVVAEFARLLFFLPFVLGYAVSNTPFLNNFYWYCILIAS
LSLTQGWLGTLAFYYAVNSVESPAERELTGPMAAIASPFGCVIGLYTAAPY
```

TGVEG_359630

Tg359630 seems to be absent in ME49, COUG, CAST and MAS strains, and only 360 a.a. long in RH

CLUSTAL O(1.2.4) multiple sequence alignment

```
FOU                       MKSLPTLTSAAGYPCSACAEGIHTPSRPNGIHLVPLSLQRAESVVSPSQSRGGPRSGRRF    60
GAB2-2007-GAL-DOM2        MKSLPTLTSAAGYPCSACAEGIHTPSRPNGIHLVPLSLQRAESVVSPSQSRGGPRSGRRF    60
GT1                       MKSLPTLTSAAGYPCSACAEGIHTPSRPNGIHLVPLSLQRAESVVSPSQSRGGPRSGRRF    60
RH                        ------------------------------------------------------------    0
CATBr9                    MKSLPTLTSAAGYPCSACAEGIHTPSRPNGIHLVPLSLQRAESVVSPSQSRGGPRSGRRF    60
VEG                       MKSLPTLTSAAGYPCSACAEGIHTPSRPNGIHLVPLSLQRAESVVSPSQSRGGPRSGRRF    60
ARI                       MKSLPTLTSAAGYPCSACAEGIHTPSRPNGIHLVPLSLQRAESVVSPSQSRGGPRSGRRF    60
CatPRC2                   MKSLPTLTSAAGYPCSACAEGIHTPSRPNGIHLVPLSLQRAESVVSPSQSRGGPRSGRRF    60
RUB                       MKSLPTLTSAAGYPCSACAEGIHTPSRPNGIHLVPLSLQRAESVVSPSQSRGGPRSGRRF    60

FOU                       PSLHTRAISEPAEARPRPLSLRPTSQPSPPPKRQSVLFRTFNLLPWHRQRTLVLPPTEET    120
GAB2-2007-GAL-DOM2        PSLHTRAISEPAEARPRPLSLRPTSQPSPPPKRQSVLFRTFNLLPWHRQRTLVLPPTEET    120
GT1                       PSLHTRAISEPAEARPRPLSLRPTSQPSPPPKRQSVLFRTFNLLPWHRQRTLVLPPTEET    120
RH                        ------------------------------------------------------------    0
CATBr9                    PSLHTRAISEPAEARPRPLSLRPTSQPSPPPKRQSVLFRTFNLLPWHRQRTLVLPPTEET    120
VEG                       PSSHTRAISEPAEARPRPLSLRPTSQPSPPPKRQSVLFRTFNLLPWHRQRTLVLPPTEET    120
ARI                       PSSHTRAISEPAEARPRPLSLRPTSQPSPPPKRQSVLFRTFNLLPWHRQRTLVLPPTEET    120
CatPRC2                   PSSHTRAISEPAEARPRPLSLRPTSQPSPPPKRQSVLFRTFNLLPWHRQRTLVLPPTEET    120
RUB                       PSLHTRAISEPAEARPRPLSLRPTSQPSPPPKRQSVLFRTFNLLPWHRQRTLVLRPTEET    120

FOU                       VAQAAASSRPTFRYPDAATLTDYSLREGLPMSTAGESAAEDLGGASAVAGRSIFEGESYE    180
GAB2-2007-GAL-DOM2        VAQAAASSRPTFRYPDAATLTDYSLREGLPMSTAGESAAEDLGGASAVAGRSIFEGESYE    180
GT1                       VAQAAASSRPTFRYPDAATLTDYSLREGLPMSTAGESAAEDLGGASAVAGRSIFEGESYE    180
RH                        ------------------------------------------------------------    0
CATBr9                    VAQAAASSRPTFRYPDAATLTDYSLREGLPMSTAGESAAEDLGGASAVAGRSIFEGESYE    180
VEG                       VAQAAASSRPTFRYPDAATLTDYSLREGLPMSTAGESAAEDLGGASAVAGRSIFEGESYE    180
ARI                       VAQAAASSRPTFRYPDAATLTDYSLREGLPMSTAGESAAEDLGGASAVAGRSIFEGESYE    180
CatPRC2                   VAQAAASSRPTFRYPDAATLTDYSLREGLPMSTAGESAAEDLGGASAVAGRSIFEGESYE    180
RUB                       VAQAAASSRPTFRYPDAATLTDYSLREGLPMSTAGESAAEDLGGASAVAGRSIFEGESYE    180

FOU                       GPESIIDGIDDICGSHGAASFSPKAARIGNTVFGLLGFSSLVGWNFTINLAPYISAQLFP    240
GAB2-2007-GAL-DOM2        GPESIIDGIDDICGSHGAASFSPKAARIGNTVFGLLGFSSLVGWNFTINLAPYISAQLFP    240
GT1                       GPESIIDGIDDICGSHGAASFSPKAARIGNTVFGLLGFSSLVGWNFTINLAPYISAQLFP    240
RH                        ------------------------------------------------------------    0
CATBr9                    GPESIIDGIDDICGSHGAASFSPKAARIGNTVFGLLGFSSLVGWNFTINLAPYISAQLFP    240
VEG                       GPESIIDGIDDICGSHGAASFSPKAARIGNTVFGLLGFSSLVGWNFTINLAPYISAQLFP    240
ARI                       GPESIIDGIDDICGSHGAASFSPKAARIGNTVFGLLGFSSLVGWNFTINLAPYISAQLFP    240
CatPRC2                   GPESIIDGIDDICGSHGAASFSPKAARIGNTVFGLLGFSSLVGWNFTINLAPYISAQLFP    240
RUB                       GPESIIDGIDDICGSHGAASFSPKAARIGNTVFGLLGFSSLVGWNFTINLAPYISAQLFP    240

FOU                       DSATHWDNSFLAMFQIAILLVQACLLWIGALRKSLLFIGGCLSVVVFSILTPVLAYLPET    300
GAB2-2007-GAL-DOM2        DSATHWDNSFLAMFQIAILLVQACLLWIGALRKSLLFIGGCLSVVVFSILTPVLAYLPET    300
GT1                       DSATHWDNSFLAMFQIAILLVQACLLWIGALRKSLLFIGGCLSVVVFSILTPVLAYLPET    300
RH                        ------------------------------------------------------------    0
CATBr9                    DSATHWDNSFLAMFQIAILLVQACLLWIGALRKSLLFIGGCLSVVVFSILTPVLAYLPET    300
VEG                       DSATHWDNSFLAMFQIAILLVQACLLWIGALRKSLLFIGGCLSVVVFSILTPVLAYLPET    300
ARI                       DSATHWDNSFLAMFQIAILLVQACLLWIGALRKSLLFIGGCLSVVVFSILTPVLAYLPET    300
CatPRC2                   DSATHWDNSFLAMFQIAILLVQACLLWIGALRKSLLFIGGCLSVVVFSILTPVLAYLPET    300
RUB                       DSATHWDNSFLAMFQIAILLVQACLLWIGALRKSLLFIGGCLSVVVFSILTPVLAYLPET    300

FOU                       VAITGMHLMCFLTGLSSGLFQGAGFAIAGAMPKTCVGAVSVGQGLAGVGAFLLSTLLGFA    360
GAB2-2007-GAL-DOM2        VAITGMHLMCFLTGLSSGLFQGAGFAIAGAMPKTCVGAVSVGQGLAGVGAFLLSTLLGFA    360
GT1                       VAITGMHLMCFLTGLSSGLFQGAGFAIAGAMPKTCVGAVSVGQGLAGVGAFLLSTLLGFA    360
RH                        --------MCFLTGLSSGLFQGAGFAIAGAMPKTCVGAVSVGQGLAGVGAFLLSTLLGFA    52
CATBr9                    VAITGMHLMCFLTGLSSGLFQGAGFAIAGAMPKTCVGAVSVGQGLAGVGAFLLSTLLGFA    360
VEG                       VAITGMHLMCFLTGLSSGLFQGAGFAIAGAMPKTCVGAVSVGQGLAGVGAFLLSTLLGFA    360
ARI                       VAITGMHLMCFLTGLSSGLFQGAGFAIAGAMPKTCVGAVSVGQGLAGVGAFLLSTLLGFA    360
CatPRC2                   VAITGMHLMCFLTGLSSGLFQGAGFAIAGAMPKTCVGAVSVGQGLAGVGAFLLSTLLGFA    360
RUB                       VAITGMHLMCFLTGLSSGLFQGAGFAIAGAMPKTCVGAVSVGQGLAGVGAFLLSTLLGFA    360
                                  ****************************************************

FOU                       VFPLDTRVGVQTTAWTVYLFSALVALLSAVSVSYLFQQSWARRALKKASQQRTERMRLSV    420
GAB2-2007-GAL-DOM2        VFPLDTRVGVQTTAWTVYLFSALVALLSAVSVSYLFQQSWARRALKKASQQRTERMRLSV    420
GT1                       VFPLDTRVGVQTTAWTVYLFSALVALLSAVSVSYLFQQSWARRALKKASQQRTERMRLSV    420
RH                        VFPLDTRVGVQTTAWTVYLFSALVALLSAVSVSYLFQQSWARRALKKASQQRTERMRLSV    112
```

```
CATBr9                  VFPLDTRVGVQTTAWTVYLFSALVALLSAVSVSYLFQQSWARRALKKASQQRTERMRLSV     420
VEG                     VFPLDTRVGVQTTAWTVYLFSALVALLSAVSVSYLFQQPWARRALKKASQQRTERMRLSV     420
ARI                     VFPLDTRVGVQTTAWTVYLFSALVALLSAVSVSYLFQQPWARRALKKASQQRTERMRLSV     420
CatPRC2                 VFPLDTRVGVQTTAWTVYLFSALVALLSAVSVSYLFQQPWARRALKKASQQRTERMRLSV     420
RUB                     VFPLDTRVGVQTTAWTVYLFSALVALLSAVSVSYLFQQPWARRALKKASQQRTERMRLSV     420
                        ************************************** *********************

FOU                     RRQQEREIDEAAAEIEAVENSVTESDDVDLEAALDEKGMSTVVHGDVGAKPISPILVEAC     480
GAB2-2007-GAL-DOM2      RRQQEREIDEAAAEIEAVENSVTESDDVDLEAALDEKGMSTVVHGDVGAKPISPILVEAC     480
GT1                     RRQQEREIDEAAAEIEAVENSVTESDDVDLEAALDEKGMSTVVHGDVGAKPISPILVEAC     480
RH                      RRQQEREIDEAAAEIEAVENSVTESDDVDLEAALDEKGMSTVVHGDVGAKPISPILVEAC     172
CATBr9                  RRQQEREIDEAAAEIEAVENSVTESDDVDLEAALDEKGMSTVVHGDVGAKPISPILVEAC     480
VEG                     RRQQEREIDEAAAEIEAVENSVTESDDVDLEAASDEEGMSTVVHGDVGAKPISPILVEAC     480
ARI                     RRQQEREIDEAAAEIEAVENSVTESDDVDLEAASDEEGMSTVVHGDVGAKPISPILVEAC     480
CatPRC2                 RRQQEREIDEAAAEIEAVENSVTESDDVDLEAASDEEGMSTVVHGDVGAKPISPILVEAC     480
RUB                     RRQQEREIDEAAADIEAVENSVTESDDVDLEAASDEEGMSTVVHGDVGAKPISPILVEAC     480
                        *************:******************* **:***********************

FOU                     AEPSSRRIRHRAPELYSKWHVLQTVFPELLSIFLNFFITMNLFPRVGPVLWKYSQNISNH     540
GAB2-2007-GAL-DOM2      AEPSSRRIRHRAPELYSKWHVLQTVFPELLSIFLNFFITMNLFPRVGPVLWKYSQNISNH     540
GT1                     AEPSSRRIRHRAPELYSKWHVLQTVFPELLSIFLNFFITMNLFPRVGPVLWKYSQNISNH     540
RH                      AEPSSRRIRHRAPELYSKWHVLQTVFPELLSIFLNFFITMNLFPRVGPVLWKYSQNISNH     232
CATBr9                  AEPSSRRIRHRAPELYSKWHVLQTVFPELLSIFLNFFITMNLFPRVGPVLWKYSQNISNH     540
VEG                     AEPSSRRIRHRAPELYSKWHVLQTVFPELLSIFLNFFITMNLFPRVGPVLWKYSQNISNH     540
ARI                     AEPSSRRIRHRAPELYSKWHVLQTVFPELLSIFLNFFITMNLFPRVGPVLWKYSQNISNH     540
CatPRC2                 AEPSSRRIRHRAPELYSKWHVLQTVFPELLSIFLNFFITMNLFPRVGPVLWKYSQNISNH     540
RUB                     AEPSSRRIRHRAPELYSKWHVLQTVFPELLSIFLNFFITMNLFPRVGPVLWKYSQNISNH     540
                        ************************************************************

FOU                     FLILFGVFSVGDVAGRWLPDLSQATTRLQWLMLPRTWLLPAVLFRTIFYVPFCLGYKVEG     600
GAB2-2007-GAL-DOM2      FLILFGVFSVGDVAGRWLPDLSQATTRLQWLMLPRTWLLPAVLFRTIFYVPFCLGYKVEG     600
GT1                     FLILFGVFSVGDVAGRWLPDLSQATTRLQWLMLPRTWLLPAVLFRTIFYVPFCLGYKVEG     600
RH                      FLILFGVFSVGDVAGRWLPDLSQATTRLQWLMLPRTWLLPAVLFRTIFYVPFCLGYKVEG     292
CATBr9                  FLILFGVFSVGDVAGRWLPDLSQATTRLQWLMLPRTWLLPAVLFRTIFYVPFCLGYKVEG     600
VEG                     FLILFGVFSVGDVAGRWLPDLSQATTRLQWLMLPRTWLLPAVLFRTIFYVPFCLGYKVEG     600
ARI                     FLILFGVFSVGDVAGRWLPDLSQATTRLQWLMLPRTWLLPAVLFRTIFYVPFCLGYKVEG     600
CatPRC2                 FLILFGVFSVGDVAGRWLPDLSQATTRLQWLMLPRTWLLPAVLFRTIFYVPFCLGYKVEG     600
RUB                     FLILFGVFSVGDVAGRWLPDLSQATTRLQWLMLPRTWLLPAVLFRTIFYVPFCLGYKVEG     600
                        ************************************************************

FOU                     APVINDFWWYVIVMFLFAVTHGWTSTLGYIYCVSIPSRLDEKEIAGPLAVIALSLGLVTG     660
GAB2-2007-GAL-DOM2      APVINDFWWYVIVMFLFAVTHGWTSTLGYIYCVSIPSRLDEKEIAGPLAVIALSLGLVTG     660
GT1                     APVINDFWWYVIVMFLFAVTHGWTSTLGYIYCVSIPSRLDEKEIAGPLAVIALSLGLVTG     660
RH                      APVINDFWWYVIVMFLFAVTHGWTSTLGYIYCVSIPSRLDEKEIAGPLAVIALSLGLVTG     352
CATBr9                  APVINDFWWYVIVMFLFAVTHGWTSTLGYIYCVSIPSRLDEKEIAGPLAVIALSLGLVTG     660
VEG                     APVINDFWWYVIVMFLFAVTHGWTSTLGYIYCVSIPSRLDEKEIAGPLAVIALSLGLVTG     660
ARI                     APVINDFWWYVIVMFLFAVTHGWTSTLGYIYCVSIPSRLDEKEIAGPLAVIALSLGLVTG     660
CatPRC2                 APVINDFWWYVIVMFLFAVTHGWTSTLGYIYCVSIPSRLDEKEIAGPLAVIALSLGLVTG     660
RUB                     APVINDFWWYVIVMFLFAVTHGWTSTLGYIYCVSIPSRLDEKEIAGPLAVIALSLGLVTG     660
                        ************************************************************

FOU                     LYIAFLVY     668
GAB2-2007-GAL-DOM2      LYIAFLVY     668
GT1                     LYIAFLVY     668
RH                      LYIAFLVY     360
CATBr9                  LYIAFLVY     668
VEG                     LYIAFLVY     668
ARI                     LYIAFLVY     668
CatPRC2                 LYIAFLVY     668
RUB                     LYIAFLVY     668
                        ********
```

TGVEG_359630 without RH

CLUSTAL O(1.2.4) multiple sequence alignment

```
FOU                         MKSLPTLTSAAGYPCSACAEGIHTPSRPNGIHLVPLSLQRAESVVSPSQSRGGPRSGRRF     60
GAB2-2007-GAL-DOM2          MKSLPTLTSAAGYPCSACAEGIHTPSRPNGIHLVPLSLQRAESVVSPSQSRGGPRSGRRF     60
GT1                         MKSLPTLTSAAGYPCSACAEGIHTPSRPNGIHLVPLSLQRAESVVSPSQSRGGPRSGRRF     60
CATBr9                      MKSLPTLTSAAGYPCSACAEGIHTPSRPNGIHLVPLSLQRAESVVSPSQSRGGPRSGRRF     60
VEG                         MKSLPTLTSAAGYPCSACAEGIHTPSRPNGIHLVPLSLQRAESVVSPSQSRGGPRSGRRF     60
ARI                         MKSLPTLTSAAGYPCSACAEGIHTPSRPNGIHLVPLSLQRAESVVSPSQSRGGPRSGRRF     60
CatPRC2                     MKSLPTLTSAAGYPCSACAEGIHTPSRPNGIHLVPLSLQRAESVVSPSQSRGGPRSGRRF     60
RUB                         MKSLPTLTSAAGYPCSACAEGIHTPSRPNGIHLVPLSLQRAESVVSPSQSRGGPRSGRRF     60
                            ************************************************************

FOU                         PSLHTRAISEPAEARPRPLSLRPTSQPSPPPKRQSVLFRTFNLLPWHRQRTLVLPPTEET     120
GAB2-2007-GAL-DOM2          PSLHTRAISEPAEARPRPLSLRPTSQPSPPPKRQSVLFRTFNLLPWHRQRTLVLPPTEET     120
GT1                         PSLHTRAISEPAEARPRPLSLRPTSQPSPPPKRQSVLFRTFNLLPWHRQRTLVLPPTEET     120
CATBr9                      PSLHTRAISEPAEARPRPLSLRPTSQPSPPPKRQSVLFRTFNLLPWHRQRTLVLPPTEET     120
VEG                         PSSHTRAISEPAEARPRPLSLRPTSQPSPPPKRQSVLFRTFNLLPWHRQRTLVLPPTEET     120
ARI                         PSSHTRAISEPAEARPRPLSLRPTSQPSPPPKRQSVLFRTFNLLPWHRQRTLVLPPTEET     120
CatPRC2                     PSSHTRAISEPAEARPRPLSLRPTSQPSPPPKRQSVLFRTFNLLPWHRQRTLVLPPTEET     120
RUB                         PSLHTRAISEPAEARPRPLSLRPTSQPSPPPKRQSVLFRTFNLLPWHRQRTLVLRPTEET     120
                            ** *************************************************** *****

FOU                         VAQAAASSRPTFRYPDAATLTDYSLREGLPMSTAGESAAEDLGGASAVAGRSIFEGESYE     180
GAB2-2007-GAL-DOM2          VAQAAASSRPTFRYPDAATLTDYSLREGLPMSTAGESAAEDLGGASAVAGRSIFEGESYE     180
GT1                         VAQAAASSRPTFRYPDAATLTDYSLREGLPMSTAGESAAEDLGGASAVAGRSIFEGESYE     180
CATBr9                      VAQAAASSRPTFRYPDAATLTDYSLREGLPMSTAGESAAEDLGGASAVAGRSIFEGESYE     180
VEG                         VAQAAASSRPTFRYPDAATLTDYSLREGLPMSTAGESAAEDLGGASAVAGRSIFEGESYE     180
ARI                         VAQAAASSRPTFRYPDAATLTDYSLREGLPMSTAGESAAEDLGGASAVAGRSIFEGESYE     180
CatPRC2                     VAQAAASSRPTFRYPDAATLTDYSLREGLPMSTAGESAAEDLGGASAVAGRSIFEGESYE     180
RUB                         VAQAAASSRPTFRYPDAATLTDYSLREGLPMSTAGESAAEDLGGASAVAGRSIFEGESYE     180
                            ************************************************************

FOU                         GPESIIDGIDDICGSHGAASFSPKAARIGNTVFGLLGFSSLVGWNFTINLAPYISAQLFP     240
GAB2-2007-GAL-DOM2          GPESIIDGIDDICGSHGAASFSPKAARIGNTVFGLLGFSSLVGWNFTINLAPYISAQLFP     240
GT1                         GPESIIDGIDDICGSHGAASFSPKAARIGNTVFGLLGFSSLVGWNFTINLAPYISAQLFP     240
CATBr9                      GPESIIDGIDDICGSHGAASFSPKAARIGNTVFGLLGFSSLVGWNFTINLAPYISAQLFP     240
VEG                         GPESIIDGIDDICGSHGAASFSPKAARIGNTVFGLLGFSSLVGWNFTINLAPYISAQLFP     240
ARI                         GPESIIDGIDDICGSHGAASFSPKAARIGNTVFGLLGFSSLVGWNFTINLAPYISAQLFP     240
CatPRC2                     GPESIIDGIDDICGSHGAASFSPKAARIGNTVFGLLGFSSLVGWNFTINLAPYISAQLFP     240
RUB                         GPESIIDGIDDICGSHGAASFSPKAARIGNTVFGLLGFSSLVGWNFTINLAPYISAQLFP     240
                            ************************************************************

FOU                         DSATHWDNSFLAMFQIAILLVQACLLWIGALRKSLLFIGGCLSVVVFSILTPVLAYLPET     300
GAB2-2007-GAL-DOM2          DSATHWDNSFLAMFQIAILLVQACLLWIGALRKSLLFIGGCLSVVVFSILTPVLAYLPET     300
GT1                         DSATHWDNSFLAMFQIAILLVQACLLWIGALRKSLLFIGGCLSVVVFSILTPVLAYLPET     300
CATBr9                      DSATHWDNSFLAMFQIAILLVQACLLWIGALRKSLLFIGGCLSVVVFSILTPVLAYLPET     300
VEG                         DSATHWDNSFLAMFQIAILLVQACLLWIGALRKSLLFIGGCLSVVVFSILTPVLAYLPET     300
ARI                         DSATHWDNSFLAMFQIAILLVQACLLWIGALRKSLLFIGGCLSVVVFSILTPVLAYLPET     300
CatPRC2                     DSATHWDNSFLAMFQIAILLVQACLLWIGALRKSLLFIGGCLSVVVFSILTPVLAYLPET     300
RUB                         DSATHWDNSFLAMFQIAILLVQACLLWIGALRKSLLFIGGCLSVVVFSILTPVLAYLPET     300
                            ************************************************************

FOU                         VAITGMHLMCFLTGLSSGLFQGAGFAIAGAMPKTCVGAVSVGQGLAGVGAFLLSTLLGFA     360
GAB2-2007-GAL-DOM2          VAITGMHLMCFLTGLSSGLFQGAGFAIAGAMPKTCVGAVSVGQGLAGVGAFLLSTLLGFA     360
GT1                         VAITGMHLMCFLTGLSSGLFQGAGFAIAGAMPKTCVGAVSVGQGLAGVGAFLLSTLLGFA     360
CATBr9                      VAITGMHLMCFLTGLSSGLFQGAGFAIAGAMPKTCVGAVSVGQGLAGVGAFLLSTLLGFA     360
VEG                         VAITGMHLMCFLTGLSSGLFQGAGFAIAGAMPKTCVGAVSVGQGLAGVGAFLLSTLLGFA     360
ARI                         VAITGMHLMCFLTGLSSGLFQGAGFAIAGAMPKTCVGAVSVGQGLAGVGAFLLSTLLGFA     360
CatPRC2                     VAITGMHLMCFLTGLSSGLFQGAGFAIAGAMPKTCVGAVSVGQGLAGVGAFLLSTLLGFA     360
RUB                         VAITGMHLMCFLTGLSSGLFQGAGFAIAGAMPKTCVGAVSVGQGLAGVGAFLLSTLLGFA     360
                            ************************************************************

FOU                         VFPLDTRVGVQTTAWTVYLFSALVALLSAVSVSYLFQQSWARRALKKASQQRTERMRLSV     420
GAB2-2007-GAL-DOM2          VFPLDTRVGVQTTAWTVYLFSALVALLSAVSVSYLFQQSWARRALKKASQQRTERMRLSV     420
GT1                         VFPLDTRVGVQTTAWTVYLFSALVALLSAVSVSYLFQQSWARRALKKASQQRTERMRLSV     420
CATBr9                      VFPLDTRVGVQTTAWTVYLFSALVALLSAVSVSYLFQQSWARRALKKASQQRTERMRLSV     420
VEG                         VFPLDTRVGVQTTAWTVYLFSALVALLSAVSVSYLFQQPWARRALKKASQQRTERMRLSV     420
ARI                         VFPLDTRVGVQTTAWTVYLFSALVALLSAVSVSYLFQQPWARRALKKASQQRTERMRLSV     420
CatPRC2                     VFPLDTRVGVQTTAWTVYLFSALVALLSAVSVSYLFQQPWARRALKKASQQRTERMRLSV     420
RUB                         VFPLDTRVGVQTTAWTVYLFSALVALLSAVSVSYLFQQPWARRALKKASQQRTERMRLSV     420
                            ************************************** *********************

FOU                         RRQQEREIDEAAAEIEAVENSVTESDDVDLEAALDEKGMSTVVHGDVGAKPISPILVEAC     480
```

```
GAB2-2007-GAL-DOM2      RRQQEREIDEAAAEIEAVENSVTESDDVDLEAALDEKGMSTVVHGDVGAKPISPILVEAC      480
GT1                     RRQQEREIDEAAAEIEAVENSVTESDDVDLEAALDEKGMSTVVHGDVGAKPISPILVEAC      480
CATBr9                  RRQQEREIDEAAAEIEAVENSVTESDDVDLEAALDEKGMSTVVHGDVGAKPISPILVEAC      480
VEG                     RRQQEREIDEAAAEIEAVENSVTESDDVDLEAASDEEGMSTVVHGDVGAKPISPILVEAC      480
ARI                     RRQQEREIDEAAAEIEAVENSVTESDDVDLEAASDEEGMSTVVHGDVGAKPISPILVEAC      480
CatPRC2                 RRQQEREIDEAAAEIEAVENSVTESDDVDLEAASDEEGMSTVVHGDVGAKPISPILVEAC      480
RUB                     RRQQEREIDEAAADIEAVENSVTESDDVDLEAASDEEGMSTVVHGDVGAKPISPILVEAC      480
                        *************:******************* **:***********************

FOU                     AEPSSRRIRHRAPELYSKWHVLQTVFPELLSIFLNFFITMNLFPRVGPVLWKYSQNISNH      540
GAB2-2007-GAL-DOM2      AEPSSRRIRHRAPELYSKWHVLQTVFPELLSIFLNFFITMNLFPRVGPVLWKYSQNISNH      540
GT1                     AEPSSRRIRHRAPELYSKWHVLQTVFPELLSIFLNFFITMNLFPRVGPVLWKYSQNISNH      540
CATBr9                  AEPSSRRIRHRAPELYSKWHVLQTVFPELLSIFLNFFITMNLFPRVGPVLWKYSQNISNH      540
VEG                     AEPSSRRIRHRAPELYSKWHVLQTVFPELLSIFLNFFITMNLFPRVGPVLWKYSQNISNH      540
ARI                     AEPSSRRIRHRAPELYSKWHVLQTVFPELLSIFLNFFITMNLFPRVGPVLWKYSQNISNH      540
CatPRC2                 AEPSSRRIRHRAPELYSKWHVLQTVFPELLSIFLNFFITMNLFPRVGPVLWKYSQNISNH      540
RUB                     AEPSSRRIRHRAPELYSKWHVLQTVFPELLSIFLNFFITMNLFPRVGPVLWKYSQNISNH      540
                        ************************************************************

FOU                     FLILFGVFSVGDVAGRWLPDLSQATTRLQWLMLPRTWLLPAVLFRTIFYVPFCLGYKVEG      600
GAB2-2007-GAL-DOM2      FLILFGVFSVGDVAGRWLPDLSQATTRLQWLMLPRTWLLPAVLFRTIFYVPFCLGYKVEG      600
GT1                     FLILFGVFSVGDVAGRWLPDLSQATTRLQWLMLPRTWLLPAVLFRTIFYVPFCLGYKVEG      600
CATBr9                  FLILFGVFSVGDVAGRWLPDLSQATTRLQWLMLPRTWLLPAVLFRTIFYVPFCLGYKVEG      600
VEG                     FLILFGVFSVGDVAGRWLPDLSQATTRLQWLMLPRTWLLPAVLFRTIFYVPFCLGYKVEG      600
ARI                     FLILFGVFSVGDVAGRWLPDLSQATTRLQWLMLPRTWLLPAVLFRTIFYVPFCLGYKVEG      600
CatPRC2                 FLILFGVFSVGDVAGRWLPDLSQATTRLQWLMLPRTWLLPAVLFRTIFYVPFCLGYKVEG      600
RUB                     FLILFGVFSVGDVAGRWLPDLSQATTRLQWLMLPRTWLLPAVLFRTIFYVPFCLGYKVEG      600
                        ************************************************************

FOU                     APVINDFWWYVIVMFLFAVTHGWTSTLGYIYCVSIPSRLDEKEIAGPLAVIALSLGLVTG      660
GAB2-2007-GAL-DOM2      APVINDFWWYVIVMFLFAVTHGWTSTLGYIYCVSIPSRLDEKEIAGPLAVIALSLGLVTG      660
GT1                     APVINDFWWYVIVMFLFAVTHGWTSTLGYIYCVSIPSRLDEKEIAGPLAVIALSLGLVTG      660
CATBr9                  APVINDFWWYVIVMFLFAVTHGWTSTLGYIYCVSIPSRLDEKEIAGPLAVIALSLGLVTG      660
VEG                     APVINDFWWYVIVMFLFAVTHGWTSTLGYIYCVSIPSRLDEKEIAGPLAVIALSLGLVTG      660
ARI                     APVINDFWWYVIVMFLFAVTHGWTSTLGYIYCVSIPSRLDEKEIAGPLAVIALSLGLVTG      660
CatPRC2                 APVINDFWWYVIVMFLFAVTHGWTSTLGYIYCVSIPSRLDEKEIAGPLAVIALSLGLVTG      660
RUB                     APVINDFWWYVIVMFLFAVTHGWTSTLGYIYCVSIPSRLDEKEIAGPLAVIALSLGLVTG      660
                        ************************************************************

FOU                     LYIAFLVY      668
GAB2-2007-GAL-DOM2      LYIAFLVY      668
GT1                     LYIAFLVY      668
CATBr9                  LYIAFLVY      668
VEG                     LYIAFLVY      668
ARI                     LYIAFLVY      668
CatPRC2                 LYIAFLVY      668
RUB                     LYIAFLVY      668
                        ********
```

```
Sequences for alignment for TGVEG_359630

>VEG
MKSLPTLTSAAGYPCSACAEGIHTPSRPNGIHLVPLSLQRAESVVSPSQSRGGPRSGRRF
PSSHTRAISEPAEARPRPLSLRPTSQPSPPPKRQSVLFRTFNLLPWHRQRTLVLPPTEET
VAQAAASSRPTFRYPDAATLTDYSLREGLPMSTAGESAAEDLGGASAVAGRSIFEGESYE
GPESIIDGIDDICGSHGAASFSPKAARIGNTVFGLLGFSSLVGWNFTINLAPYISAQLFP
DSATHWDNSFLAMFQIAILLVQACLLWIGALRKSLLFIGGCLSVVVFSILTPVLAYLPET
VAITGMHLMCFLTGLSSGLFQGAGFAIAGAMPKTCVGAVSVGQGLAGVGAFLLSTLLGFA
VFPLDTRVGVQTTAWTVYLFSALVALLSAVSVSYLFQQPWARRALKKASQQRTERMRLSV
RRQQEREIDEAAAEIEAVENSVTESDDVDLEAASDEEGMSTVVHGDVGAKPISPILVEAC
AEPSSRRIRHRAPELYSKWHVLQTVFPELLSIFLNFFITMNLFPRVGPVLWKYSQNISNH
FLILFGVFSVGDVAGRWLPDLSQATTRLQWLMLPRTWLLPAVLFRTIFYVPFCLGYKVEG
APVINDFWWYVIVMFLFAVTHGWTSTLGYIYCVSIPSRLDEKEIAGPLAVIALSLGLVTG
LYIAFLVY

>ARI
MKSLPTLTSAAGYPCSACAEGIHTPSRPNGIHLVPLSLQRAESVVSPSQSRGGPRSGRRF
PSSHTRAISEPAEARPRPLSLRPTSQPSPPPKRQSVLFRTFNLLPWHRQRTLVLPPTEET
VAQAAASSRPTFRYPDAATLTDYSLREGLPMSTAGESAAEDLGGASAVAGRSIFEGESYE
GPESIIDGIDDICGSHGAASFSPKAARIGNTVFGLLGFSSLVGWNFTINLAPYISAQLFP
DSATHWDNSFLAMFQIAILLVQACLLWIGALRKSLLFIGGCLSVVVFSILTPVLAYLPET
VAITGMHLMCFLTGLSSGLFQGAGFAIAGAMPKTCVGAVSVGQGLAGVGAFLLSTLLGFA
VFPLDTRVGVQTTAWTVYLFSALVALLSAVSVSYLFQQPWARRALKKASQQRTERMRLSV
RRQQEREIDEAAAEIEAVENSVTESDDVDLEAASDEEGMSTVVHGDVGAKPISPILVEAC
AEPSSRRIRHRAPELYSKWHVLQTVFPELLSIFLNFFITMNLFPRVGPVLWKYSQNISNH
FLILFGVFSVGDVAGRWLPDLSQATTRLQWLMLPRTWLLPAVLFRTIFYVPFCLGYKVEG
APVINDFWWYVIVMFLFAVTHGWTSTLGYIYCVSIPSRLDEKEIAGPLAVIALSLGLVTG
LYIAFLVY

>FOU
MKSLPTLTSAAGYPCSACAEGIHTPSRPNGIHLVPLSLQRAESVVSPSQSRGGPRSGRRF
PSLHTRAISEPAEARPRPLSLRPTSQPSPPPKRQSVLFRTFNLLPWHRQRTLVLPPTEET
VAQAAASSRPTFRYPDAATLTDYSLREGLPMSTAGESAAEDLGGASAVAGRSIFEGESYE
GPESIIDGIDDICGSHGAASFSPKAARIGNTVFGLLGFSSLVGWNFTINLAPYISAQLFP
DSATHWDNSFLAMFQIAILLVQACLLWIGALRKSLLFIGGCLSVVVFSILTPVLAYLPET
VAITGMHLMCFLTGLSSGLFQGAGFAIAGAMPKTCVGAVSVGQGLAGVGAFLLSTLLGFA
VFPLDTRVGVQTTAWTVYLFSALVALLSAVSVSYLFQQSWARRALKKASQQRTERMRLSV
RRQQEREIDEAAAEIEAVENSVTESDDVDLEAALDEKGMSTVVHGDVGAKPISPILVEAC
AEPSSRRIRHRAPELYSKWHVLQTVFPELLSIFLNFFITMNLFPRVGPVLWKYSQNISNH
FLILFGVFSVGDVAGRWLPDLSQATTRLQWLMLPRTWLLPAVLFRTIFYVPFCLGYKVEG
APVINDFWWYVIVMFLFAVTHGWTSTLGYIYCVSIPSRLDEKEIAGPLAVIALSLGLVTG
LYIAFLVY

>GAB2-2007-GAL-DOM2
MKSLPTLTSAAGYPCSACAEGIHTPSRPNGIHLVPLSLQRAESVVSPSQSRGGPRSGRRF
PSLHTRAISEPAEARPRPLSLRPTSQPSPPPKRQSVLFRTFNLLPWHRQRTLVLPPTEET
VAQAAASSRPTFRYPDAATLTDYSLREGLPMSTAGESAAEDLGGASAVAGRSIFEGESYE
GPESIIDGIDDICGSHGAASFSPKAARIGNTVFGLLGFSSLVGWNFTINLAPYISAQLFP
DSATHWDNSFLAMFQIAILLVQACLLWIGALRKSLLFIGGCLSVVVFSILTPVLAYLPET
VAITGMHLMCFLTGLSSGLFQGAGFAIAGAMPKTCVGAVSVGQGLAGVGAFLLSTLLGFA
VFPLDTRVGVQTTAWTVYLFSALVALLSAVSVSYLFQQSWARRALKKASQQRTERMRLSV
RRQQEREIDEAAAEIEAVENSVTESDDVDLEAALDEKGMSTVVHGDVGAKPISPILVEAC
AEPSSRRIRHRAPELYSKWHVLQTVFPELLSIFLNFFITMNLFPRVGPVLWKYSQNISNH
FLILFGVFSVGDVAGRWLPDLSQATTRLQWLMLPRTWLLPAVLFRTIFYVPFCLGYKVEG
APVINDFWWYVIVMFLFAVTHGWTSTLGYIYCVSIPSRLDEKEIAGPLAVIALSLGLVTG
LYIAFLVY

>GT1
MKSLPTLTSAAGYPCSACAEGIHTPSRPNGIHLVPLSLQRAESVVSPSQSRGGPRSGRRF
PSLHTRAISEPAEARPRPLSLRPTSQPSPPPKRQSVLFRTFNLLPWHRQRTLVLPPTEET
VAQAAASSRPTFRYPDAATLTDYSLREGLPMSTAGESAAEDLGGASAVAGRSIFEGESYE
GPESIIDGIDDICGSHGAASFSPKAARIGNTVFGLLGFSSLVGWNFTINLAPYISAQLFP
DSATHWDNSFLAMFQIAILLVQACLLWIGALRKSLLFIGGCLSVVVFSILTPVLAYLPET
VAITGMHLMCFLTGLSSGLFQGAGFAIAGAMPKTCVGAVSVGQGLAGVGAFLLSTLLGFA
VFPLDTRVGVQTTAWTVYLFSALVALLSAVSVSYLFQQSWARRALKKASQQRTERMRLSV
RRQQEREIDEAAAEIEAVENSVTESDDVDLEAALDEKGMSTVVHGDVGAKPISPILVEAC
AEPSSRRIRHRAPELYSKWHVLQTVFPELLSIFLNFFITMNLFPRVGPVLWKYSQNISNH
FLILFGVFSVGDVAGRWLPDLSQATTRLQWLMLPRTWLLPAVLFRTIFYVPFCLGYKVEG
APVINDFWWYVIVMFLFAVTHGWTSTLGYIYCVSIPSRLDEKEIAGPLAVIALSLGLVTG
LYIAFLVY

>RH
MCFLTGLSSGLFQGAGFAIAGAMPKTCVGAVSVGQGLAGVGAFLLSTLLGFAVFPLDTRV
GVQTTAWTVYLFSALVALLSAVSVSYLFQQSWARRALKKASQQRTERMRLSVRRQQEREI
DEAAAEIEAVENSVTESDDVDLEAALDEKGMSTVVHGDVGAKPISPILVEACAEPSSRRI
RHRAPELYSKWHVLQTVFPELLSIFLNFFITMNLFPRVGPVLWKYSQNISNHFLILFGVF
```

SVGDVAGRWLPDLSQATTRLQWLMLPRTWLLPAVLFRTIFYVPFCLGYKVEGAPVINDFW
WYVIVMFLFAVTHGWTSTLGYIYCVSIPSRLDEKEIAGPLAVIALSLGLVTGLYIAFLVY

>RUB
MKSLPTLTSAAGYPCSACAEGIHTPSRPNGIHLVPLSLQRAESVVSPSQSRGGPRSGRRF
PSLHTRAISEPAEARPRPLSLRPTSQPSPPPKRQSVLFRTFNLLPWHRQRTLVLRPTEET
VAQAAASSRPTFRYPDAATLTDYSLREGLPMSTAGESAAEDLGGASAVAGRSIFEGESYE
GPESIIDGIDDICGSHGAASFSPKAARIGNTVFGLLGFSSLVGWNFTINLAPYISAQLFP
DSATHWDNSFLAMFQIAILLVQACLLWIGALRKSLLFIGGCLSVVVFSILTPVLAYLPET
VAITGMHLMCFLTGLSSGLFQGAGFAIAGAMPKTCVGAVSVGQGLAGVGAFLLSTLLGFA
VFPLDTRVGVQTTAWTVYLFSALVALLSAVSVSYLFQQPWARRALKKASQQRTERMRLSV
RRQQEREIDEAAADIEAVENSVTESDDVDLEAASDEEGMSTVVHGDVGAKPISPILVEAC
AEPSSRRIRHRAPELYSKWHVLQTVFPELLSIFLNFFITMNLFPRVGPVLWKYSQNISNH
FLILFGVFSVGDVAGRWLPDLSQATTRLQWLMLPRTWLLPAVLFRTIFYVPFCLGYKVEG
APVINDFWWYVIVMFLFAVTHGWTSTLGYIYCVSIPSRLDEKEIAGPLAVIALSLGLVTG
LYIAFLVY

>CATBr9
MKSLPTLTSAAGYPCSACAEGIHTPSRPNGIHLVPLSLQRAESVVSPSQSRGGPRSGRRF
PSLHTRAISEPAEARPRPLSLRPTSQPSPPPKRQSVLFRTFNLLPWHRQRTLVLPPTEET
VAQAAASSRPTFRYPDAATLTDYSLREGLPMSTAGESAAEDLGGASAVAGRSIFEGESYE
GPESIIDGIDDICGSHGAASFSPKAARIGNTVFGLLGFSSLVGWNFTINLAPYISAQLFP
DSATHWDNSFLAMFQIAILLVQACLLWIGALRKSLLFIGGCLSVVVFSILTPVLAYLPET
VAITGMHLMCFLTGLSSGLFQGAGFAIAGAMPKTCVGAVSVGQGLAGVGAFLLSTLLGFA
VFPLDTRVGVQTTAWTVYLFSALVALLSAVSVSYLFQQSWARRALKKASQQRTERMRLSV
RRQQEREIDEAAAEIEAVENSVTESDDVDLEAALDEKGMSTVVHGDVGAKPISPILVEAC
AEPSSRRIRHRAPELYSKWHVLQTVFPELLSIFLNFFITMNLFPRVGPVLWKYSQNISNH
FLILFGVFSVGDVAGRWLPDLSQATTRLQWLMLPRTWLLPAVLFRTIFYVPFCLGYKVEG
APVINDFWWYVIVMFLFAVTHGWTSTLGYIYCVSIPSRLDEKEIAGPLAVIALSLGLVTG
LYIAFLVY

>CatPRC2
MKSLPTLTSAAGYPCSACAEGIHTPSRPNGIHLVPLSLQRAESVVSPSQSRGGPRSGRRF
PSSHTRAISEPAEARPRPLSLRPTSQPSPPPKRQSVLFRTFNLLPWHRQRTLVLPPTEET
VAQAAASSRPTFRYPDAATLTDYSLREGLPMSTAGESAAEDLGGASAVAGRSIFEGESYE
GPESIIDGIDDICGSHGAASFSPKAARIGNTVFGLLGFSSLVGWNFTINLAPYISAQLFP
DSATHWDNSFLAMFQIAILLVQACLLWIGALRKSLLFIGGCLSVVVFSILTPVLAYLPET
VAITGMHLMCFLTGLSSGLFQGAGFAIAGAMPKTCVGAVSVGQGLAGVGAFLLSTLLGFA
VFPLDTRVGVQTTAWTVYLFSALVALLSAVSVSYLFQQPWARRALKKASQQRTERMRLSV
RRQQEREIDEAAAEIEAVENSVTESDDVDLEAASDEEGMSTVVHGDVGAKPISPILVEAC
AEPSSRRIRHRAPELYSKWHVLQTVFPELLSIFLNFFITMNLFPRVGPVLWKYSQNISNH
FLILFGVFSVGDVAGRWLPDLSQATTRLQWLMLPRTWLLPAVLFRTIFYVPFCLGYKVEG
APVINDFWWYVIVMFLFAVTHGWTSTLGYIYCVSIPSRLDEKEIAGPLAVIALSLGLVTG
LYIAFLVY

>VAND
MKSLPTLTSAAGYPCSACAEGIHTPSRPNGIHLVPLSLQRAESVVSPSQSRGGPRSGRRF
PSLHTRAISEPAEARPRPLSLRPTSQPSPPPKRQSVLFRTFNLLPWHRQRTLVLPPTEET
VAQAAASSRPTFRYPDAATLTDYSLREGLPMSTAGESAAEDLGGASAVAGRSIFEGESYE
GPESIIDGIDDICGSHGAASFSPKAARIGNTVFGLLGFSSLVGWNFTINLAPYISAQLFP
DSATHWDNSFLAMFQIAILLVQACLLWIGALRKSLLFIGGCLSVVVFSILTPVLAYLPET
VAITGMHLMCFLTGLSSGLFQGAGFAIAGAMPKTCVGAVSVGQGLAGVGAFLLSTLLGFA
VFPLDTRVGVQTTAWTVYLFSALVALLSAVSVSYLFQQPWARRALKKASQQRTERMRLSV
RRQQEREIDEAAAEIEAVENSVTESDDVDLEAASDEEGMSTVVHGDVGAKPISPILVEAC
AEPSSRRIRHRAPELYSKWHVLQTVFPELLSIFLNFFITMNLFPRVGPVLWKYSQNISNH
FLILFGVFSVGDVAGRWLPDLSQATTRLQWLMLPRTWLLPAVLFRTIFYVPFCLGYKVEG
APVINDFWWYVIVMFLFAVTHGWTSTLGYIYCVSVPSRLDEKEIAGPLAVIALSLGLVTG
LYIAFLVY

>p89
MPKTCVGAVSVGQGLAGVGAFLLSTLLGFAVFPLDTRVGVQTTAWTVYLFSALVALLSAV
SVSYLFQQPWARRALKKASQQRTERMRLSVRRQQEREIDEAAAEIEAVENSVTESDDVDL
EAASDEEGMSTVVHGDVGAKPISPILVEACAEPSSRRIRHRAPELYSKWHVLQTVFPELL
SIFLNFFITMNLFPRVGPVLWKYSQNISNHFLILFGVFSVGDVAGRWLPDLSQATTRLQW
LMLPRTWLLPAVLFRTIFYVPFCLGYKVEGAPVINDFWWYVIVMFLFAVTHGWTSTLGYI
YCVSIPSRLDEKEIAGPLAVIALSLGLVTGLYIAFLVY

TGP89_418410
nucleoside transporter
Toxoplasma gondii
p89
Non-Reference Strain
338 aa

MPKTCVGAVSVGQGLAGVGAFLLSTLLGFAVFPLDTRVGVQTTAWTVYLFSALVALLSAVSVSYLFQQPWARRALKKASQ
QRTERMRLSVRRQQEREIDEAAAEIEAVENSVTESDDVDLEAASDEEGMSTVVHGDVGAKPISPILVEACAEPSSRRIRH
RAPELYSKWHVLQTVFPELLSIFLNFFITMNLFPRVGPVLWKYSQNISNHFLILFGVFSVGDVAGRWLPDLSQATTRLQW
LMLPRTWLLPAVLFRTIFYVPFCLGYKVEGAPVINDFWWYVIVMFLFAVTHGWTSTLGYIYCVSIPSRLDEKEIAGPLAV
IALSLGLVTGLYIAFLVY

TGVAND_359630
nucleoside transporter
Toxoplasma gondii
VAND
Non-Reference Strain
668 aa

MKSLPTLTSAAGYPCSACAEGIHTPSRPNGIHLVPLSLQRAESVVSPSQSRGGPRSGRRFPSLHTRAISEPAEARPRPLS
LRPTSQPSPPPKRQSVLFRTFNLLPWHRQRTLVLPPTEETVAQAAASSRPTFRYPDAATLTDYSLREGLPMSTAGESAAE
DLGGASAVAGRSIFEGESYEGPESIIDGIDDICGSHGAASFSPKAARIGNTVFGLLGFSSLVGWNFTINLAPYISAQLFP
DSATHWDNSFLAMFQIAILLVQACLLWIGALRKSLLFIGGCLSVVVFSILTPVLAYLPETVAITGMHLMCFLTGLSSGLF
QGAGFAIAGAMPKTCVGAVSVGQGLAGVGAFLLSTLLGFAVFPLDTRVGVQTTAWTVYLFSALVALLSAVSVSYLFQQPW
ARRALKKASQQRTERMRLSVRRQQEREIDEAAAEIEAVENSVTESDDVDLEAASDEEGMSTVVHGDVGAKPISPILVEAC
AEPSSRRIRHRAPELYSKWHVLQTVFPELLSIFLNFFITMNLFPRVGPVLWKYSQNISNHFLILFGVFSVGDVAGRWLPD
LSQATTRLQWLMLPRTWLLPAVLFRTIFYVPFCLGYKVEGAPVINDFWWYVIVMFLFAVTHGWTSTLGYIYCVSVPSRLD
EKEIAGPLAVIALSLGLVTGLYIAFLVY

TGVEG_288540

```
CLUSTAL O(1.2.4) multiple sequence alignment


CatPRC2                 MAGLDTLALEDVDLDGPLSSSIGESCSPRSRGDSILASQQDAHPPVAYKAAFLHCLTPPS    60
ME49                    MAGLDTLALEDVDLDGPLSSSIGESCSPRSRGDSILASQQDAHPPVAYKAAFLHCLTPPS    60
VEG                     MAGLDTLALEDVDLDGPLSSSIGESCSPRSRGDSILASQQDAHPPVAYKAAFLHCLTPPS    60
ARI                     MAGLDTLALEDVDLDGPLSSSIGESCSPRSRGDSILASQQDAHPPVAYKAAFLHCLTPPS    60
CAST                    MAGLDTLALEDVDLDGPLSSSIGESCSPRSRGDSILASQQDAHPPVAYKAAFLHCLTPPS    60
RH                      MAGLDTLALEDVDLDGPLSSSIGESCSPRSRGDSILASQQDAHPPVAYKAAFLHCLTPPS    60
VAND                    MAGLDTLALEDVDLDGPLSSSIGESCSPRSRGDSILASQQDAHPPVAYKAAFLHCLTPPS    60
COUG                    MAGLDTLALEDVDLDGPLSSSIGESCSPRSRGDSILASQQDAHPPVAYKAAFLHCLTPPS    60
FOU                     MAGLDTLALEDVDLDGPLSSSIGESCSPRSRGDSILASQQDAHPPVAYKAAFLHCLTPPS    60
GAB2-2007-GAL-DOM2      MAGLDTLALEDVDLDGPLSSSIGESCSPRSRGDSILASQQDAHPPVAYKAAFLHCLTPPS    60
GT1                     MAGLDTLALEDVDLDGPLSSSIGESCSPRSRGDSILASQQDAHPPVAYKAAFLHCLTPPS    60
p89                     MAGLDTLALEDVDLDGPLSSSIGESCSPRSRGDSILASQQDAHPPVAYKAAFLHCLTPPS    60
CATBr9                  MAGLDTLALEDVDLDGPLSSSIGESCSPRSRGDSILASQQDAHPPVAYKAAFLHCLTPPS    60
MAS                     MAGLDTLALEDVDLDGPLSSSIGESCSPRSRGDSILASQQDAHPPVAYKAAFLHCLTPPS    60
                        ************************************************************

CatPRC2                 MRSNSWTECLEGQDDTSLCSNVLRKTSSNVSVCRNSNGKENAPGSNPCPIPRASVASDRE    120
ME49                    MRSNSWTECLEGQDDTSLCSNVLRKTSSNVSVCRNSNGKENAPGSNPCPIPRASVASDRE    120
VEG                     MRSNSWTECLEGQDDTSLCSNVLRKTSSNVSVCRNSNGKENAPGSNPCPIPRASVASDRE    120
ARI                     MRSNSWTECLEGQDDTSLCSNVLRKTSSNVSVCRNSNGKENAPGSNPCPIPRASVASDRE    120
CAST                    MRSNSWTECLEGQDDTSLCSNVLRKTSSNVSVCRNSNGKENAPGSNPCPIPRASVASDRE    120
RH                      MRSNSWTECLEGQDDTSLCSNVLRKTSSNVSVCRNSNGKENAPGSNPCPIPRASVASDRE    120
VAND                    MRSNSWTECLEGQDDTSLCSNVLRKTSSNVSVCRNSNGKENAPGSNPCPIPRASVASDRE    120
COUG                    MRSNSWTECLEGQDDTSLCSNVLRKTSSNVSVCRNSNGKENAPGSNPCPIPRASVASDRE    120
FOU                     MRSNSWTECLEGQDDTSLCSNVLRKTSSNVSVCRNSNGKENAPGSNPCPIPRASVASDRE    120
GAB2-2007-GAL-DOM2      MRSNSWTECLEGQDDTSLCSNVLRKTSSNVSVCRNSNGKENAPGSNPCPIPRASVASDRE    120
GT1                     MRSNSWTECLEGQDDTSLCSNVLRKTSSNVSVCRNSNGKENAPGSNPCPIPRASVASDRE    120
p89                     MRSNSWTECLEGQDDTSLCSNVLRKTSSNVSVCRNSNGKENAPGSNPCPIPRASVASDRE    120
CATBr9                  MRSNSWTECLEGQDDTSLCSNVLRKTSSNVSVCRNSNGKENAPGSNPCPIPRASVASDRE    120
MAS                     MRSNSWTECLEGQDDTSLCSNVLRKTSSNVSVCRNSNGKENAPGSNPCPIPRASVASDRE    120
                        ************************************************************

CatPRC2                 TPCHSEQKSCPFTERDRVAGFLTFLVCGVASLSCWQFLLSMTPYIEMTFFQSTPIGNSLL    180
ME49                    TPCHSEQKSCPFTERDRVAGFLTFLVCGVASLSCWQFLLSMTPYIEMTFFQSTPIGNSLL    180
VEG                     TPCHSEQKSCPFTERDRVAGFLTFLVCGVASLSCWQFLLSMTPYIEMTFFQSTPIGNSLL    180
ARI                     TPCHSEQKSCPFTERDRVAGFLTFLVCGVASLSCWQFLLSMTPYIEMTFFQSTPIGNSLL    180
CAST                    TPCHSEQKSCPFTERDRVAGFLTFLVCGVASLSCWQFLLSMTPYIEMTFFQSTPIGNSLL    180
RH                      TPCHSEQKSCPFTERDRVAGFLTFLVCGVASLSCWQFLLSMTPYIEMTFFQSTPIGNSLL    180
VAND                    TPCHSEQKSCPFTERDRVAGFLTFLVCGVASLSCWQFLLSMTPYIEMTFFQSTPIGNSLL    180
COUG                    TPCHSEQKSCPFTERDRVAGFLTFLVCGVASLSCWQFLLSMTPYIEMTFFQSTPIGNSLL    180
FOU                     TPCHSEQKSCPFTERDRVAGFLTFLVCGVASLSCWQFLLSMTPYIEMTFFQSTPIGNSLL    180
GAB2-2007-GAL-DOM2      TPCHSEQKSCPFTERDRVAGFLTFLVCGVASLSCWQFLLSMTPYIEMTFFQSTPIGNSLL    180
GT1                     TPCHSEQKSCPFTERDRVAGFLTFLVCGVASLSCWQFLLSMTPYIEMTFFQSTPIGNSLL    180
p89                     TPCHSEQKSCPFTERDRVAGFLTFLVCGVASLSCWQFLLSMTPYIEMTFFQSTPIGNSLL    180
CATBr9                  TPCHSEQKSCPFTERDRVAGFLTFLVCGVASLSCWQFLLSMTPYIEMTFFQSTPIGNSLL    180
MAS                     TPCHSEQKSCPFTERDRVAGFLTFLVCGVASLSCWQFLLSMTPYIEMTFFQSTPIGNSLL    180
                        ************************************************************

CatPRC2                 GVYQVGCICVQLVLMFLVDSMQPWIVILATLVDAGLAVTFPLVVTLVPDVAKAALMHIVS    240
ME49                    GVYQVGCICVQLVLMFLVDSMQPWIVILATLVDAGLAVTFPLVVTLVPDVAKAALMHIVS    240
VEG                     GVYQVGCICVQLVLMFLVDSMQPWIVILATLVDAGLAVTFPLVVTLVPDVAKAALMHIVS    240
ARI                     GVYQVGCICVQLVLMFLVDSMQPWIVILATLVDAGLAVTFPLVVTLVPDVAKAALMHIVS    240
CAST                    GVYQVGCICVQLVLMFLVDSMQPWIVILATLVDAGLAVTFPLVVTLVPDVAKAALMHIVS    240
RH                      GVYQVGCICVQLVLMFLVDSMQPWIVILATLVDAGLAVTFPLVVTLVPDVAKAALMHIVS    240
VAND                    GVYQVGCICVQLVLMFLVDSMQPWIVILATLVDAGLAVTFPLVVTLVPDVAKAALMHIVS    240
COUG                    GVYQVGCICVQLVLMFLVDSMQPWIVILATLVDAGLAVTFPLVVTLVPDVAKAALMHIVS    240
FOU                     GVYQVGCICVQLVLMFLVDSMQPWIVILATLVDAGLAVTFPLVVTLVPDVAKAALMHIVS    240
GAB2-2007-GAL-DOM2      GVYQVGCICVQLVLMFLVDSMQPWIVILATLVDAGLAVTFPLVVTLVPDVAKAALMHIVS    240
GT1                     GVYQVGCICVQLVLMFLVDSMQPWIVILATLVDAGLAVTFPLVVTLVPDVAKAALMHIVS    240
p89                     GVYQVGCICVQLVLMFLVDSMQPWIVILATLVDAGLAVTFPLVVTLVPDVAKAALMHIVS    240
CATBr9                  GVYQVGCICVQLVLMFLVDSMQPWIVILATLVDAGLAVTFPLVVTLVPDVAKAALMHIVS    240
MAS                     GVYQVGCICVQLVLMFLVDSMQPWIVILATLVDAGLAVTFPLVVTLVPDVAKAALMHIVS    240
                        ************************************************************

CatPRC2                 LLFGVSAGVICGGSIPVASAMPYNFIGSFSMGQGVAGILSFSVNLVFSFSFDLGSEEGVS    300
ME49                    LLFGVSAGVICGGSIPVASAMPYNFIGSFSMGQGVAGILSFSVNLVFSFSFDLGSEEGVS    300
VEG                     LLFGVSAGVICGGSIPVASAMPYNFIGSFSMGQGVAGILSFSVNLVFSFLFDLGSEEGVS    300
ARI                     LLFGVSAGVICGGSIPVASAMPYNFIGSFSMGQGVAGILSFSVNLVFSFLFDLGSEEGVS    300
CAST                    LLFGVSAGVICGGSIPVASAMPYNFIGSFSMGQGVAGILSFSVNLVFSFLFDLGSEEGVS    300
RH                      LLFGVSAGVICGGSIPVASAMPYNFIGSFSMGQGVAGILSFSVNLVFSFLFDLGSEEGVS    300
VAND                    LLFGVSAGVICGGSIPVASAMPYNFIGSFSMGQGVAGILSFSVNLVFSFLFDLGSEEGVS    300
```

```
COUG                  LLFGVSAGVICGGSIPVASAMPYNFIGSFSMGQGVAGILSFSVNLVFSFLFDLGSEEGVS   300
FOU                   LLFGVSAGVICGGSIPVASAMPYNFIGSFSMGQGVAGILSFSVNLVFSFLFDLGSEEGVS   300
GAB2-2007-GAL-DOM2    LLFGVSAGVICGGSIPVASAMPYNFIGSFSMGQGVAGILSFSVNLVFSFLFDLGSEEGVS   300
GT1                   LLFGVSAGVICGGSIPVASAMPYNFIGSFSMGQGVAGILSFSVNLVFSFLFDLGSEEGVS   300
p89                   LLFGVSAGVICGGSIPVASAMPYNFIGSFSMGQGVAGILSFSVNLVFSFLFDLGSEEGVS   300
CATBr9                LLFGVSAGVICGGSIPVASAMPYNFIGSFSMGQGVAGILSFSVNLVFSFLFDLGSEEGVS   300
MAS                   LLFGVSAGVICGGSIPVASAMPYNFIGSFSMGQGVAGILSFSVNLVFSFLFDLGSEEGVS   300
                      ************************************************* **********

CatPRC2               SMLWLVFGISSVVSIVSAGLLFFAVRQPWAARQLTRYWEAKRSRRQGSRWAEIKRRWRCD   360
ME49                  SMLWLVFGISSVVSIVSAGLLFFAVRQPWAARQLTRYWEAKRSRRQGSRWAEIKRRWRCD   360
VEG                   SMLWLVFGISSVVSIVSAGLLFFAVRQPWAARQLTRYWEAKRSRRQGSRWAEIKRRWRCD   360
ARI                   SMLWLVFGISSVVSIVSAGLLFFAVRQPWAARQLTRYWEAKRSRRQGSRWAEIKRRWRCD   360
CAST                  SMLWLVFGISSVVSIVSAGLLFFAVRQPWAARQLTRYWEAKRSRRQGSRWAEIKRRWRCD   360
RH                    SMLWLVFGISSVVSIVSAGLLFFAVRQPWAARQLTRYWEAKRSRRQGSRWAEIKRRWRCD   360
VAND                  SMLWLVFGISSVVSIVSAGLLFFAVRQPWAARQLTRYWEAKRSRRQGSRWAEIKRRWRCD   360
COUG                  SMLWLVFGISSVVSIVSAGLLFFAVRQPWAARQLTRYWEAKRSRRQGSRWAEIKRRWRCD   360
FOU                   SMLWLVFGISSVVSIVSAGLLFFAVRQPWAARQLTRYWEAKRSRRQGSRWAEIKRRWRCD   360
GAB2-2007-GAL-DOM2    SMLWLVFGISSVVSIVSAGLLFFAVRQPWAARQLTRYWEAKRSRRQGSRWAEIKRRWRCD   360
GT1                   SMLWLVFGISSVVSIVSAGLLFFAVRQPWAARQLTRYWEAKRSRRQGSRWAEIKRRWRCD   360
p89                   SMLWLVFGISSVVSIVSAGLLFFAVRQPWAARQLTRYWEAKRSRRQGSRWAEIKRRWRCD   360
CATBr9                SMLWLVFGISSVVSIVSAGLLFFAVRQPWAARQLTRYWEAKRSRRQGSRWAEIKRRWRCD   360
MAS                   SMLWLVFGISSVVSIVSAGLLFFAVRQPWAARQLTRYWEAKRSRRQGSRWAEIKRRWRCD   360
                      ************************************************************

CatPRC2               AAGRELPVIQNEVFVDESANRTAHAIELKKQNLQSSEKKSAVHYDVDAADQTARSRGNSS   420
ME49                  AAGRELPVIQNEVFVDESANRTAHAIELKKQNLQSSEKKSAVHYDVDAADQTARSRGNSS   420
VEG                   AAGRELPVIQNEVFVDESANRTAHAIELKKQNLQSSEKKSAVHYDVDAADQTARSRGNSS   420
ARI                   AAGRELPVIQNEVFVDESANRTAHAIELKKQNLQSSEKKSAVHYDVDAADQTARSRGNSS   420
CAST                  AAGRELPVIQNEVFVDESANRTAHAIELKKQNLQSSEKKSAVHYDVDAADQTARSRGNSS   420
RH                    AAGRELPVIQNEVFVDESANRTAHAIELKKQNLQSSEKKSAVHYDVDAADQTARSRGNSS   420
VAND                  AAGRELPVIQNEVFVDESANRTAHAIELKKQNLQSSEKKSAVHYDVDAADQTARSRGNSS   420
COUG                  AAGRELPVIQNEVFVDESANRTAHAIELKKQNLQSSEKKSAVHYDVDAADQTARSRGNSS   420
FOU                   AAGRELPVIQNEVFVDESANRTAHAIELKKQNLQSSEKKSAVHYDVDAADQTARSRGNSS   420
GAB2-2007-GAL-DOM2    AAGRELPVIQNEVFVDESANRTAHAIELKKQNLQSSEKKSAVHYDVDAADQTARSRGNSS   420
GT1                   AAGRELPVIQNEVFVDESANRTAHAIELKKQNLQSSEKKSAVHYDVDAADQTARSRGNSS   420
p89                   AAGRELPVIQNEVFVDESANRTAHAIELKKQNLQSSEKKSAVHYDVDAADQTARSRGNSS   420
CATBr9                AAGRELPVIQNEVFVDESANRTAHAIELKKQNLQSSEKKSAVHYDVDAADQTARSRGNSS   420
MAS                   AAGRELPVIQNEVFVDESANRTAHAIELKKQNLQSSEKKSAVHYDVDAADQTARSRGNSS   420
                      ************************************************************

CatPRC2               VAVSLPVSKVEFAASEEQKQQSSEVAEEKSHGISVFGKRKQGDAVGLRLSGKVQAEQSGR   480
ME49                  VAVSLPVSKVEFAASEEQKQQSSEVAEEKSHGISVFGKRKQGDAVGLRLSGKVQAEQSGR   480
VEG                   VAVSLPVSKVEFAASEEQKQQSSEVAEEKSHGISVFGKRKQGDAVGLRLSGKVQAEQSGR   480
ARI                   VAVSLPVSKVEFAASEEQKQQSSEVAEEKSHGISVFGKRKQGDAVGLRLSGKVQAEQSGR   480
CAST                  VAVSLPVSKVEFAASEEQKQQSSEVAEEKSHGISVFGKRKQGDAVGLRLSGKVQAEQSGR   480
RH                    VAVSLPVSKVEFAASEEQKQQSSEVAEEKSHGISVFGKRKQGDAVGLRLSGKVQAEQSGR   480
VAND                  VAVSLPVSKVEFAASEEQKQQSSEVAEEKSHGISVFGKRKQGDAVGLRLSGKVQAEQSGR   480
COUG                  VAVSLPVSKVEFAASEEQKQQSSEVAEEKSHGISVFGKRKQGDAVGLRLSGKVQAEQSGR   480
FOU                   VAVSLPVSKVEFAASEEQKQQSSEVAEEKSHGISVFGKRKQGDAVGLRLSGKVQAEQSGR   480
GAB2-2007-GAL-DOM2    VAVSLPVSKVEFAASEEQKQQSSEVAEEKSHGISVFGKRKQGDAVGLRLSGKVQAEQSGR   480
GT1                   VAVSLPVSKVEFAASEEQKQQSSEVAEEKSHGISVFGKRKQGDAVGLRLSGKVQAEQSGR   480
p89                   VAVSLPVSKVEFAASEEQKQQSSEVAEEKSHGISVFGKRKQGDAVGLRLSGKVQAEQSGR   480
CATBr9                VAVSLPVSKVEFAASEEQKQQSSEVAEEKSHGISVFGKRKQGDAVGLRLSGKVQAEQSER   480
MAS                   VAVSLPVSKVEFAASEEQKQQSSEVAEEKSHGISVFGKRKQGDAVGLRLSGKVQAEQSER   480
                      ********************************************************** *

CatPRC2               GAAAGQTFSSRPAGSELCVGSREGTECELNLDMVNDECHWPSRGWRYFLRDSGIFLFCVF   540
ME49                  GAAAGQTFSSRPAGSELCVGSREGTECELNLDMVNDECHWPSRGWRYFLRDSGIFLFCVF   540
VEG                   GAAAGQTFSSRPAGSELCVGSREGTECELNLDMVNDECHWPSRGWRYFLRDSGIFLFCVF   540
ARI                   GAAAGQTFSSRPAGSELCVGSREGTECELNLDMVNDECHWPSRGWRYFLRDSGIFLFCVF   540
CAST                  GAAAGQTFSSRPAGSELCVGSREGTECELNLDMVNDECHWPSRGWRYFLRDSGIFLFCVF   540
RH                    GAAAGQTFSSRPAGSELCVGSREGTECELNLDMVNDECHWPSRGWRYFLRDSGIFLFCVF   540
VAND                  GAAAGQTFSSRPAGSELCVGSREGTECELNLDMVNDECHWPSRGWRYFLRDSGIFLFCVF   540
COUG                  GAAAGQTFSSRPAGSELCVGSREGTECELNLDMVNDECHWPSRGWRYFLRDSGIFLFCVF   540
FOU                   GAAAGQTFSSRPAGSELCVGSREGTECELNLDMVNDECHWPSRGWRYFLRDSGIFLFCVF   540
GAB2-2007-GAL-DOM2    GAAAGQTFSSRPAGSELCVGSREGTECELNLDMVNDECHWPSRGWRYFLRDSGIFLFCVF   540
GT1                   GAAAGQTFSSRPAGSELCVGSREGTECELNLDMVNDECHWPSRGWRYFLRDSGIFLFCVF   540
p89                   GAAAGQTFSSRPAGSELCVGSREGTECELNLDMVNDECHWPSRGWRYFLRDSGIFLFCVF   540
CATBr9                GAAAGQTFSSRPAGSELCVGSREGTECELNLDMVNDECHWPSRGWRYFLRDSGIFLFCVF   540
MAS                   GAAAGQTFSSRPAGSELCVGSREGTECELNLDMVNDECHWPSRGWRYFLRDSGIFLFCVF   540
                      ************************************************************

CatPRC2               FNFFVTLNLFPRVGPIMWHYPGFAKNGPQYIILFGLFSIGDLCGKSLPDLATLSPRVGRW   600
ME49                  FNFFVTLNLFPRVGPIMWHYPGFAKNGPQYIILFGLFSIGDLCGKSLPDLATLSPRVGRW   600
VEG                   FNFFVTLNLFPRVGPIMWHYPGFAKNGPQYIILFGLFSIGDLCGKSLPDLATLSPRVGRW   600
ARI                   FNFFVTLNLFPRVGPIMWHYPGFAKNGPQYIILFGLFSIGDLCGKSLPDLATLSPRVGRW   600
```

```
CAST                      FNFFVTLNLFPRVGPIMWHYPGFAKNGPQYIILFGLFSIGDLCGKSLPDLATLSPRVGRW      600
RH                        FNFFVTLNLFPRVGPIMWHYPGFAKNGPQYIILFGLFSIGDLCGKSLPDLATLSPRVGRW      600
VAND                      FNFFVTLNLFPRVGPIMWHYPGFAKNGPQYIILFGLFSIGDLCGKSLPDLATLSPRVGRW      600
COUG                      FNFFVTLNLFPRVGPIMWHYPGFAKNGPQYIILFGLFSIGDLCGKSLPDLATLSPRVGRW      600
FOU                       FNFFVTLNLFPRVGPIMWHYPGFAKNGPQYIILFGLFSIGDLCGKSLPDLATLSPRVGRW      600
GAB2-2007-GAL-DOM2        FNFFVTLNLFPRVGPIMWHYPGFAKNGPQYIILFGLFSIGDLCGKSLPDLATLSPRVGRW      600
GT1                       FNFFVTLNLFPRVGPIMWHYPGFAKNGPQYIILFGLFSIGDLCGKSLPDLATLSPRVGRW      600
p89                       FNFFVTLNLFPRVGPIMWHYPGFAKNGPQYIILFGLFSIGDLCGKSLPDLATLSPRVGRW      600
CATBr9                    FNFFVTLNLFPRVGPIMWHYPGFAKNGPQYIILFGLFSIGDLCGKSLPDLATLSPRVGRW      600
MAS                       FNFFVTLNLFPRVGPIMWHYPGFAKNGPQYIILFGLFSIGDLCGKSLPDLATLSPRVGRW      600
                          ************************************************************

CatPRC2                   LTIPEKLLLPIVLSRVVLAVFFLGAYLVNAFFNSFALYVILILLLSVTSGWCATASMVY      660
ME49                      LTIPEKLLLPIVLSRVVLAVFFLGAYLVNAFFNSFALYVILILLLSVTSGWCATASMVY      660
VEG                       LTIPEKLLLPIVLSRVVLAVFFLGAYLVNAFFNSFALYVILILLLSVTSGWCATASMVY      660
ARI                       LTIPEKLLLPIVLSRVVLAVFFLGAYLVNAFFNSFALYVILILLLSVTSGWCATASMVY      660
CAST                      LTIPEKLLLPIVLSRVVLAVFFLGAYLVNAFFNSFALYVILILLLSVTSGWCATASMVY      660
RH                        LTIPEKLLLPIVLSRVVLAVFFLGAYLVNAFFNSFALYVILILLLSVTSGWCATASMVY      660
VAND                      LTIPEKLLLPIVLSRVVLAVFFLGAYLVNAFFNSFALYVILILLLSVTSGWCATASMVY      660
COUG                      LTIPEKLLLPIVLSRVVLAVFFLGAYLVNAFFNSFALYVILILLLSVTSGWCATASMVY      660
FOU                       LTIPEKLLLPIVLSRVVLAVFFLGAYLVNAFFNSFALYVILILLLSVTSGWCATASMVY      660
GAB2-2007-GAL-DOM2        LTIPEKLLLPIVLSRVVLAVFFLGAYLVNAFFNSFALYVILILLLSVTSGWCATASMVY      660
GT1                       LTIPEKLLLPIVLSRVVLAVFFLGAYLVNAFFNSFALYVILILLLSVTSGWCATASMVY      660
p89                       LTIPEKLLLPIVLSRVVLAVFFLGAYLVNAFFNSFALYVILILLLSVTSGWCATASMVY      660
CATBr9                    LTIPEKLLLPIVLSRVVLAVFFLGAYLVNAFFNSFALYVILILLLSVTSGWCATASMVY      660
MAS                       LTIPEKLLLPIVLSRVVLAVFFLGAYLVNAFFNSFALYVILILLLSVTSGWCATASMVY      660
                          ************************************************************

CatPRC2                   ACSSVKRFEEKEIVGPMSVLMLLVGIMAGVYSALAY        696
ME49                      ACSSVKRFEEKEIVGPMSVLMLLVGIMAGVYSALAY        696
VEG                       ACSSVKRFEEKEIVGPMSVLMLLVGIMAGVYSALAY        696
ARI                       ACSSVKRFEEKEIVGPMSVLMLLVGIMAGVYSALAY        696
CAST                      ACSSVKRFEEKEIVGPMSVLMLLVGIMAGVYSALAY        696
RH                        ACSSVKRFEEKEIVGPMSVLMLLVGIMAGVYSALAY        696
VAND                      ACSSVKRFEEKEIVGPMSVLMLLVGIMAGVYSALAY        696
COUG                      ACSSVKRFEEKEIVGPMSVLMLLVGIMAGVYSALAY        696
FOU                       ACSSVKRFEEKEIVGPMSVLMLLVGIMAGVYSALAY        696
GAB2-2007-GAL-DOM2        ACSSVKRFEEKEIVGPMSVLMLLVGIMAGVYSALAY        696
GT1                       ACSSVKRFEEKEIVGPMSVLMLLVGIMAGVYSALAY        696
p89                       ACSSVKRFEEKEIVGPMSVLMLLVGIMAGVYSALAY        696
CATBr9                    ACSSVKRFEEKEIVGPMSVLMLLVGIMAGVYSALAY        696
MAS                       ACSSVKRFEEKEIVGPMSVLMLLVGIMAGVYSALAY        696
                          ************************************
```

Sequences for alignment for TGVEG_288540:

>VEG
MAGLDTLALEDVDLDGPLSSSIGESCSPRSRGDSILASQQDAHPPVAYKAAFLHCLTPPS
MRSNSWTECLEGQDDTSLCSNVLRKTSSNVSVCRNSNGKENAPGSNPCPIPRASVASDRE
TPCHSEQKSCPFTERDRVAGFLTFLVCGVASLSCWQFLLSMTPYIEMTFFQSTPIGNSLL
GVYQVGCICVQLVLMFLVDSMQPWIVILATLVDAGLAVTFPLVVTLVPDVAKAALMHIVS
LLFGVSAGVICGGSIPVASAMPYNFIGSFSMGQGVAGILSFSVNLVFSFLFDLGSEEGVS
SMLWLVFGISSVVSIVSAGLLFFAVRQPWAARQLTRYWEAKRSRRQGSRWAEIKRRWRCD
AAGRELPVIQNEVFVDESANRTAHAIELKKQNLQSSEKKSAVHYDVDAADQTARSRGNSS
VAVSLPVSKVEFAASEEQKQQSSEVAEEKSHGISVFGKRKQGDAVGLRLSGKVQAEQSGR
GAAAGQTFSSRPAGSELCVGSREGTECELNLDMVNDECHWPSRGWRYFLRDSGIFLFCVF
FNFFVTLNLFPRVGPIMWHYPGFAKNGPQYIILFGLFSIGDLCGKSLPDLATLSPRVGRW
LTIPEKLLLPIVLSRVVLAVFFLLGAYLVNAFFNSFALYVILILLLSVTSGWCATASMVY
ACSSVKRFEEKEIVGPMSVLMLLVGIMAGVYSALAY

>ARI
MAGLDTLALEDVDLDGPLSSSIGESCSPRSRGDSILASQQDAHPPVAYKAAFLHCLTPPS
MRSNSWTECLEGQDDTSLCSNVLRKTSSNVSVCRNSNGKENAPGSNPCPIPRASVASDRE
TPCHSEQKSCPFTERDRVAGFLTFLVCGVASLSCWQFLLSMTPYIEMTFFQSTPIGNSLL
GVYQVGCICVQLVLMFLVDSMQPWIVILATLVDAGLAVTFPLVVTLVPDVAKAALMHIVS
LLFGVSAGVICGGSIPVASAMPYNFIGSFSMGQGVAGILSFSVNLVFSFLFDLGSEEGVS
SMLWLVFGISSVVSIVSAGLLFFAVRQPWAARQLTRYWEAKRSRRQGSRWAEIKRRWRCD
AAGRELPVIQNEVFVDESANRTAHAIELKKQNLQSSEKKSAVHYDVDAADQTARSRGNSS
VAVSLPVSKVEFAASEEQKQQSSEVAEEKSHGISVFGKRKQGDAVGLRLSGKVQAEQSGR
GAAAGQTFSSRPAGSELCVGSREGTECELNLDMVNDECHWPSRGWRYFLRDSGIFLFCVF
FNFFVTLNLFPRVGPIMWHYPGFAKNGPQYIILFGLFSIGDLCGKSLPDLATLSPRVGRW
LTIPEKLLLPIVLSRVVLAVFFLLGAYLVNAFFNSFALYVILILLLSVTSGWCATASMVY
ACSSVKRFEEKEIVGPMSVLMLLVGIMAGVYSALAY

>CAST
MAGLDTLALEDVDLDGPLSSSIGESCSPRSRGDSILASQQDAHPPVAYKAAFLHCLTPPS
MRSNSWTECLEGQDDTSLCSNVLRKTSSNVSVCRNSNGKENAPGSNPCPIPRASVASDRE
TPCHSEQKSCPFTERDRVAGFLTFLVCGVASLSCWQFLLSMTPYIEMTFFQSTPIGNSLL
GVYQVGCICVQLVLMFLVDSMQPWIVILATLVDAGLAVTFPLVVTLVPDVAKAALMHIVS
LLFGVSAGVICGGSIPVASAMPYNFIGSFSMGQGVAGILSFSVNLVFSFLFDLGSEEGVS
SMLWLVFGISSVVSIVSAGLLFFAVRQPWAARQLTRYWEAKRSRRQGSRWAEIKRRWRCD
AAGRELPVIQNEVFVDESANRTAHAIELKKQNLQSSEKKSAVHYDVDAADQTARSRGNSS
VAVSLPVSKVEFAASEEQKQQSSEVAEEKSHGISVFGKRKQGDAVGLRLSGKVQAEQSGR
GAAAGQTFSSRPAGSELCVGSREGTECELNLDMVNDECHWPSRGWRYFLRDSGIFLFCVF
FNFFVTLNLFPRVGPIMWHYPGFAKNGPQYIILFGLFSIGDLCGKSLPDLATLSPRVGRW
LTIPEKLLLPIVLSRVVLAVFFLLGAYLVNAFFNSFALYVILILLLSVTSGWCATASMVY
ACSSVKRFEEKEIVGPMSVLMLLVGIMAGVYSALAY

>RH
MAGLDTLALEDVDLDGPLSSSIGESCSPRSRGDSILASQQDAHPPVAYKAAFLHCLTPPS
MRSNSWTECLEGQDDTSLCSNVLRKTSSNVSVCRNSNGKENAPGSNPCPIPRASVASDRE
TPCHSEQKSCPFTERDRVAGFLTFLVCGVASLSCWQFLLSMTPYIEMTFFQSTPIGNSLL
GVYQVGCICVQLVLMFLVDSMQPWIVILATLVDAGLAVTFPLVVTLVPDVAKAALMHIVS
LLFGVSAGVICGGSIPVASAMPYNFIGSFSMGQGVAGILSFSVNLVFSFLFDLGSEEGVS
SMLWLVFGISSVVSIVSAGLLFFAVRQPWAARQLTRYWEAKRSRRQGSRWAEIKRRWRCD
AAGRELPVIQNEVFVDESANRTAHAIELKKQNLQSSEKKSAVHYDVDAADQTARSRGNSS
VAVSLPVSKVEFAASEEQKQQSSEVAEEKSHGISVFGKRKQGDAVGLRLSGKVQAEQSGR
GAAAGQTFSSRPAGSELCVGSREGTECELNLDMVNDECHWPSRGWRYFLRDSGIFLFCVF
FNFFVTLNLFPRVGPIMWHYPGFAKNGPQYIILFGLFSIGDLCGKSLPDLATLSPRVGRW
LTIPEKLLLPIVLSRVVLAVFFLLGAYLVNAFFNSFALYVILILLLSVTSGWCATASMVY
ACSSVKRFEEKEIVGPMSVLMLLVGIMAGVYSALAY

>CATBr9
MAGLDTLALEDVDLDGPLSSSIGESCSPRSRGDSILASQQDAHPPVAYKAAFLHCLTPPS
MRSNSWTECLEGQDDTSLCSNVLRKTSSNVSVCRNSNGKENAPGSNPCPIPRASVASDRE
TPCHSEQKSCPFTERDRVAGFLTFLVCGVASLSCWQFLLSMTPYIEMTFFQSTPIGNSLL
GVYQVGCICVQLVLMFLVDSMQPWIVILATLVDAGLAVTFPLVVTLVPDVAKAALMHIVS
LLFGVSAGVICGGSIPVASAMPYNFIGSFSMGQGVAGILSFSVNLVFSFLFDLGSEEGVS
SMLWLVFGISSVVSIVSAGLLFFAVRQPWAARQLTRYWEAKRSRRQGSRWAEIKRRWRCD
AAGRELPVIQNEVFVDESANRTAHAIELKKQNLQSSEKKSAVHYDVDAADQTARSRGNSS
VAVSLPVSKVEFAASEEQKQQSSEVAEEKSHGISVFGKRKQGDAVGLRLSGKVQAEQSER
GAAAGQTFSSRPAGSELCVGSREGTECELNLDMVNDECHWPSRGWRYFLRDSGIFLFCVF
FNFFVTLNLFPRVGPIMWHYPGFAKNGPQYIILFGLFSIGDLCGKSLPDLATLSPRVGRW
LTIPEKLLLPIVLSRVVLAVFFLLGAYLVNAFFNSFALYVILILLLSVTSGWCATASMVY
ACSSVKRFEEKEIVGPMSVLMLLVGIMAGVYSALAY

>CatPRC2
MAGLDTLALEDVDLDGPLSSSIGESCSPRSRGDSILASQQDAHPPVAYKAAFLHCLTPPS
MRSNSWTECLEGQDDTSLCSNVLRKTSSNVSVCRNSNGKENAPGSNPCPIPRASVASDRE
TPCHSEQKSCPFTERDRVAGFLTFLVCGVASLSCWQFLLSMTPYIEMTFFQSTPIGNSLL
GVYQVGCICVQLVLMFLVDSMQPWIVILATLVDAGLAVTFPLVVTLVPDVAKAALMHIVS

LLFGVSAGVICGGSIPVASAMPYNFIGSFSMGQGVAGILSFSVNLVFSFSFDLGSEEGVS
SMLWLVFGISSVVSIVSAGLLFFAVRQPWAARQLTRYWEAKRSRRQGSRWAEIKRRWRCD
AAGRELPVIQNEVFVDESANRTAHAIELKKQNLQSSEKKSAVHYDVDAADQTARSRGNSS
VAVSLPVSKVEFAASEEQKQQSSEVAEEKSHGISVFGKRKQGDAVGLRLSGKVQAEQSGR
GAAAGQTFSSRPAGSELCVGSREGTECELNLDMVNDECHWPSRGWRYFLRDSGIFLFCVF
FNFFVTLNLFPRVGPIMWHYPGFAKNGPQYIILFGLFSIGDLCGKSLPDLATLSPRVGRW
LTIPEKLLLPIVLSRVVLAVFFLLGAYLVNAFFNSFALYVILILLLSVTSGWCATASMVY
ACSSVKRFEEKEIVGPMSVLMLLVGIMAGVYSALAY

>VAND
MAGLDTLALEDVDLDGPLSSSIGESCSPRSRGDSILASQQDAHPPVAYKAAFLHCLTPPS
MRSNSWTECLEGQDDTSLCSNVLRKTSSNVSVCRNSNGKENAPGSNPCPIPRASVASDRE
TPCHSEQKSCPFTERDRVAGFLTFLVCGVASLSCWQFLLSMTPYIEMTFFQSTPIGNSLL
GVYQVGCICVQLVLMFLVDSMQPWIVILATLVDAGLAVTFPLVVTLVPDVAKAALMHIVS
LLFGVSAGVICGGSIPVASAMPYNFIGSFSMGQGVAGILSFSVNLVFSFLFDLGSEEGVS
SMLWLVFGISSVVSIVSAGLLFFAVRQPWAARQLTRYWEAKRSRRQGSRWAEIKRRWRCD
AAGRELPVIQNEVFVDESANRTAHAIELKKQNLQSSEKKSAVHYDVDAADQTARSRGNSS
VAVSLPVSKVEFAASEEQKQQSSEVAEEKSHGISVFGKRKQGDAVGLRLSGKVQAEQSGR
GAAAGQTFSSRPAGSELCVGSREGTECELNLDMVNDECHWPSRGWRYFLRDSGIFLFCVF
FNFFVTLNLFPRVGPIMWHYPGFAKNGPQYIILFGLFSIGDLCGKSLPDLATLSPRVGRW
LTIPEKLLLPIVLSRVVLAVFFLLGAYLVNAFFNSFALYVILILLLSVTSGWCATASMVY
ACSSVKRFEEKEIVGPMSVLMLLVGIMAGVYSALAY

>COUG
MAGLDTLALEDVDLDGPLSSSIGESCSPRSRGDSILASQQDAHPPVAYKAAFLHCLTPPS
MRSNSWTECLEGQDDTSLCSNVLRKTSSNVSVCRNSNGKENAPGSNPCPIPRASVASDRE
TPCHSEQKSCPFTERDRVAGFLTFLVCGVASLSCWQFLLSMTPYIEMTFFQSTPIGNSLL
GVYQVGCICVQLVLMFLVDSMQPWIVILATLVDAGLAVTFPLVVTLVPDVAKAALMHIVS
LLFGVSAGVICGGSIPVASAMPYNFIGSFSMGQGVAGILSFSVNLVFSFLFDLGSEEGVS
SMLWLVFGISSVVSIVSAGLLFFAVRQPWAARQLTRYWEAKRSRRQGSRWAEIKRRWRCD
AAGRELPVIQNEVFVDESANRTAHAIELKKQNLQSSEKKSAVHYDVDAADQTARSRGNSS
VAVSLPVSKVEFAASEEQKQQSSEVAEEKSHGISVFGKRKQGDAVGLRLSGKVQAEQSGR
GAAAGQTFSSRPAGSELCVGSREGTECELNLDMVNDECHWPSRGWRYFLRDSGIFLFCVF
FNFFVTLNLFPRVGPIMWHYPGFAKNGPQYIILFGLFSIGDLCGKSLPDLATLSPRVGRW
LTIPEKLLLPIVLSRVVLAVFFLLGAYLVNAFFNSFALYVILILLLSVTSGWCATASMVY
ACSSVKRFEEKEIVGPMSVLMLLVGIMAGVYSALAY

>FOU
MAGLDTLALEDVDLDGPLSSSIGESCSPRSRGDSILASQQDAHPPVAYKAAFLHCLTPPS
MRSNSWTECLEGQDDTSLCSNVLRKTSSNVSVCRNSNGKENAPGSNPCPIPRASVASDRE
TPCHSEQKSCPFTERDRVAGFLTFLVCGVASLSCWQFLLSMTPYIEMTFFQSTPIGNSLL
GVYQVGCICVQLVLMFLVDSMQPWIVILATLVDAGLAVTFPLVVTLVPDVAKAALMHIVS
LLFGVSAGVICGGSIPVASAMPYNFIGSFSMGQGVAGILSFSVNLVFSFLFDLGSEEGVS
SMLWLVFGISSVVSIVSAGLLFFAVRQPWAARQLTRYWEAKRSRRQGSRWAEIKRRWRCD
AAGRELPVIQNEVFVDESANRTAHAIELKKQNLQSSEKKSAVHYDVDAADQTARSRGNSS
VAVSLPVSKVEFAASEEQKQQSSEVAEEKSHGISVFGKRKQGDAVGLRLSGKVQAEQSGR
GAAAGQTFSSRPAGSELCVGSREGTECELNLDMVNDECHWPSRGWRYFLRDSGIFLFCVF
FNFFVTLNLFPRVGPIMWHYPGFAKNGPQYIILFGLFSIGDLCGKSLPDLATLSPRVGRW
LTIPEKLLLPIVLSRVVLAVFFLLGAYLVNAFFNSFALYVILILLLSVTSGWCATASMVY
ACSSVKRFEEKEIVGPMSVLMLLVGIMAGVYSALAY

>GAB2-2007-GAL-DOM2
MAGLDTLALEDVDLDGPLSSSIGESCSPRSRGDSILASQQDAHPPVAYKAAFLHCLTPPS
MRSNSWTECLEGQDDTSLCSNVLRKTSSNVSVCRNSNGKENAPGSNPCPIPRASVASDRE
TPCHSEQKSCPFTERDRVAGFLTFLVCGVASLSCWQFLLSMTPYIEMTFFQSTPIGNSLL
GVYQVGCICVQLVLMFLVDSMQPWIVILATLVDAGLAVTFPLVVTLVPDVAKAALMHIVS
LLFGVSAGVICGGSIPVASAMPYNFIGSFSMGQGVAGILSFSVNLVFSFLFDLGSEEGVS
SMLWLVFGISSVVSIVSAGLLFFAVRQPWAARQLTRYWEAKRSRRQGSRWAEIKRRWRCD
AAGRELPVIQNEVFVDESANRTAHAIELKKQNLQSSEKKSAVHYDVDAADQTARSRGNSS
VAVSLPVSKVEFAASEEQKQQSSEVAEEKSHGISVFGKRKQGDAVGLRLSGKVQAEQSGR
GAAAGQTFSSRPAGSELCVGSREGTECELNLDMVNDECHWPSRGWRYFLRDSGIFLFCVF
FNFFVTLNLFPRVGPIMWHYPGFAKNGPQYIILFGLFSIGDLCGKSLPDLATLSPRVGRW
LTIPEKLLLPIVLSRVVLAVFFLLGAYLVNAFFNSFALYVILILLLSVTSGWCATASMVY
ACSSVKRFEEKEIVGPMSVLMLLVGIMAGVYSALAY

>GT1
MAGLDTLALEDVDLDGPLSSSIGESCSPRSRGDSILASQQDAHPPVAYKAAFLHCLTPPS
MRSNSWTECLEGQDDTSLCSNVLRKTSSNVSVCRNSNGKENAPGSNPCPIPRASVASDRE
TPCHSEQKSCPFTERDRVAGFLTFLVCGVASLSCWQFLLSMTPYIEMTFFQSTPIGNSLL
GVYQVGCICVQLVLMFLVDSMQPWIVILATLVDAGLAVTFPLVVTLVPDVAKAALMHIVS
LLFGVSAGVICGGSIPVASAMPYNFIGSFSMGQGVAGILSFSVNLVFSFLFDLGSEEGVS
SMLWLVFGISSVVSIVSAGLLFFAVRQPWAARQLTRYWEAKRSRRQGSRWAEIKRRWRCD
AAGRELPVIQNEVFVDESANRTAHAIELKKQNLQSSEKKSAVHYDVDAADQTARSRGNSS
VAVSLPVSKVEFAASEEQKQQSSEVAEEKSHGISVFGKRKQGDAVGLRLSGKVQAEQSGR
GAAAGQTFSSRPAGSELCVGSREGTECELNLDMVNDECHWPSRGWRYFLRDSGIFLFCVF
FNFFVTLNLFPRVGPIMWHYPGFAKNGPQYIILFGLFSIGDLCGKSLPDLATLSPRVGRW
LTIPEKLLLPIVLSRVVLAVFFLLGAYLVNAFFNSFALYVILILLLSVTSGWCATASMVY

ACSSVKRFEEKEIVGPMSVLMLLVGIMAGVYSALAY

>MAS
MAGLDTLALEDVDLDGPLSSSIGESCSPRSRGDSILASQQDAHPPVAYKAAFLHCLTPPS
MRSNSWTECLEGQDDTSLCSNVLRKTSSNVSVCRNSNGKENAPGSNPCPIPRASVASDRE
TPCHSEQKSCPFTERDRVAGFLTFLVCGVASLSCWQFLLSMTPYIEMTFFQSTPIGNSLL
GVYQVGCICVQLVLMFLVDSMQPWIVILATLVDAGLAVTFPLVVTLVPDVAKAALMHIVS
LLFGVSAGVICGGSIPVASAMPYNFIGSFSMGQGVAGILSFSVNLVFSFLFDLGSEEGVS
SMLWLVFGISSVVSIVSAGLLFFAVRQPWAARQLTRYWEAKRSRRQGSRWAEIKRRWRCD
AAGRELPVIQNEVFVDESANRTAHAIELKKQNLQSSEKKSAVHYDVDAADQTARSRGNSS
VAVSLPVSKVEFAASEEQKQQSSEVAEEKSHGISVFGKRKQGDAVGLRLSGKVQAEQSER
GAAAGQTFSSRPAGSELCVGSREGTECELNLDMVNDECHWPSRGWRYFLRDSGIFLFCVF
FNFFVTLNLFPRVGPIMWHYPGFAKNGPQYIILFGLFSIGDLCGKSLPDLATLSPRVGRW
LTIPEKLLLPIVLSRVVLAVFFLLGAYLVNAFFNSFALYVILILLLSVTSGWCATASMVY
ACSSVKRFEEKEIVGPMSVLMLLVGIMAGVYSALAY

>ME49
MAGLDTLALEDVDLDGPLSSSIGESCSPRSRGDSILASQQDAHPPVAYKAAFLHCLTPPS
MRSNSWTECLEGQDDTSLCSNVLRKTSSNVSVCRNSNGKENAPGSNPCPIPRASVASDRE
TPCHSEQKSCPFTERDRVAGFLTFLVCGVASLSCWQFLLSMTPYIEMTFFQSTPIGNSLL
GVYQVGCICVQLVLMFLVDSMQPWIVILATLVDAGLAVTFPLVVTLVPDVAKAALMHIVS
LLFGVSAGVICGGSIPVASAMPYNFIGSFSMGQGVAGILSFSVNLVFSFSFDLGSEEGVS
SMLWLVFGISSVVSIVSAGLLFFAVRQPWAARQLTRYWEAKRSRRQGSRWAEIKRRWRCD
AAGRELPVIQNEVFVDESANRTAHAIELKKQNLQSSEKKSAVHYDVDAADQTARSRGNSS
VAVSLPVSKVEFAASEEQKQQSSEVAEEKSHGISVFGKRKQGDAVGLRLSGKVQAEQSGR
GAAAGQTFSSRPAGSELCVGSREGTECELNLDMVNDECHWPSRGWRYFLRDSGIFLFCVF
FNFFVTLNLFPRVGPIMWHYPGFAKNGPQYIILFGLFSIGDLCGKSLPDLATLSPRVGRW
LTIPEKLLLPIVLSRVVLAVFFLLGAYLVNAFFNSFALYVILILLLSVTSGWCATASMVY
ACSSVKRFEEKEIVGPMSVLMLLVGIMAGVYSALAY

>p89 (696 a.a.)
MAGLDTLALEDVDLDGPLSSSIGESCSPRSRGDSILASQQDAHPPVAYKAAFLHCLTPPS
MRSNSWTECLEGQDDTSLCSNVLRKTSSNVSVCRNSNGKENAPGSNPCPIPRASVASDRE
TPCHSEQKSCPFTERDRVAGFLTFLVCGVASLSCWQFLLSMTPYIEMTFFQSTPIGNSLL
GVYQVGCICVQLVLMFLVDSMQPWIVILATLVDAGLAVTFPLVVTLVPDVAKAALMHIVS
LLFGVSAGVICGGSIPVASAMPYNFIGSFSMGQGVAGILSFSVNLVFSFLFDLGSEEGVS
SMLWLVFGISSVVSIVSAGLLFFAVRQPWAARQLTRYWEAKRSRRQGSRWAEIKRRWRCD
AAGRELPVIQNEVFVDESANRTAHAIELKKQNLQSSEKKSAVHYDVDAADQTARSRGNSS
VAVSLPVSKVEFAASEEQKQQSSEVAEEKSHGISVFGKRKQGDAVGLRLSGKVQAEQSGR
GAAAGQTFSSRPAGSELCVGSREGTECELNLDMVNDECHWPSRGWRYFLRDSGIFLFCVF
FNFFVTLNLFPRVGPIMWHYPGFAKNGPQYIILFGLFSIGDLCGKSLPDLATLSPRVGRW
LTIPEKLLLPIVLSRVVLAVFFLLGAYLVNAFFNSFALYVILILLLSVTSGWCATASMVY
ACSSVKRFEEKEIVGPMSVLMLLVGIMAGVYSALAY

>RUB (424 aa)
QGVAGILSFSVNLVFSFLFDLGSEEGVSSMLWLVFGISSVVSIVSAGLLFFAVRQPWAARQLTRYWEAKRSRRQGSRWAE
IKRRWRCDAAGRELPVIQNEVFVDESANRTAHAIELKKQNLQSSEKKSAVHYDVDAADQTARSRGNSSVAVSLPVSKVEF
AASEEQKQQSSEVAEEKSHGISVFGKRKQGDAVGLRLSGKVQAEQSGRGAAAGQTFSSRPAGSELCVGSREGTECELNLD
MVNDECHWPSRGWRYFLRDSGIFLFCVFFNFFVTLNLFPRVGPIMWHYPGFAKNGPQYIILFGLFSIGDLCGKSLPDLAT
LSPRVGRWLTIPEKLLLPIVLSRVVLAVFFLLGAYLVNAFFNSFALYVILILLLSVTSGWCATASMVYACSSVKRFEEKE
IVGPMSVLMLLVGIMAGVYSALAY
