# Supplementary material for: A Toxoplasma gondii Oxopurine Transporter Binds Nucleobases and Nucleosides Using Different Binding Modes
Source: Int J Mol Sci. 2022 Jan 10;23(2):710. doi: 10.3390/ijms23020710 (PMC8776092; doi:10.3390/ijms23020710)
Supplement: Supplementary file 1 [file ijms-23-00710-s001.zip › Supplemental File S2.pdf]

Supplemental File 2. Synthesis of new nucleoside analogues for this study.

General experimental section

Reagents and solvents of analytical grade were purchased from commercial sources and used as received. Moisture-sensitive reactions were conducted under a protective argon or nitrogen atmosphere. Reactions were performed at ambient temperature, unless specifically mentioned otherwise in the experimental procedure. Analytical TLC was performed on precoated Machery-Nagel® F254 aluminum plates which were developed by UV visualization, followed by staining with basic aq. $KMnO_4$. Column chromatography was performed using MachereyNagel® 60M silica gel (40-63 µm). Alternatively, column chromatography was performed on an automated Reveleris X2 (Grace/Büchi) Flash unit system with pre-packed silica columns.

Exact mass measurements were recorded on a Waters LCT Premier XE™ Time of Flight (ToF) mass spectrometer equipped with a standard electrospray (ESI) and modular Lockspray™ interface. Samples were infused as a solution of MeCN/water (1:1) + 0.1 % formic acid mixture at a flow rate of 100 µL/min.

NMR spectra were recorded on a Bruker Avance® Neo 400 MHz spectrometer. Chemical shifts (δ) are reported in ppm with spectra referenced to the residual solvent peak. Coupling constants are given in Hz.

Reaction monitoring and purity assessment were performed on an analytical LC/MS system (Waters AutoPurification system, equipped with ACQUITY QDa (mass; 100 – 1000 amu) and 2998 Photodiode Array (220 – 400 nm)). A Waters Cortecs® C18 (2.7 µm 100x4.6 mm) column was employed with a gradient system of HCOOH in H_2O (0.2 %, v/v)/MeCN at a flow rate of 1.44 mL/min, 95:05 to 00:100 in 6.5 minutes or a gradient of H_2O/MeCN 100:0 to 00:100 in 6.5 minutes at a flow rate of 1.44 mL/min. All obtained final compounds had purity > 95 %, as assayed by analytical HPLC (UV); unless otherwise specifically mentioned.

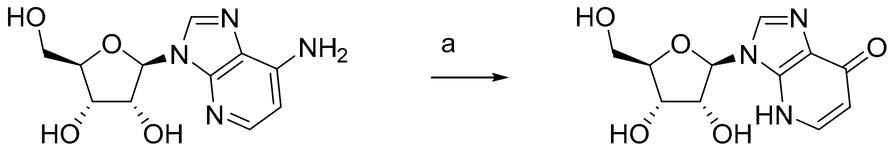

FH15946 FH15949

Scheme 1: reagents and conditions: a) $NaNO_2$, AcOH, H_2O

Starting material FH15946 was prepared according to Boehr et al., 2005. FH15946 (0.050 g, 0.19 mmol, 1 eq.) was suspended in water (1.0 mL, 5.0 mL/mmol), to which AcOH (2.8 mL, 15 mL/mmol) was added. $NaNO_2$ (0.13 g, 1.9 mmol, 10 eq.) was added and the resulting mixture stirred at ambient temperature for ~2 h. The mixture was then evaporated to dryness and purified by column

chromatography 10 → 40% MeOH/EA to give FH15949 (0.040 g, 0.15 mmol) as a slightly yellow powder in 78% yield.

^{1}H NMR (400 MHz, DMSO-d6): δ = 3.55 (dd, *J* = 12.0, 3.0 Hz, 1H, H-5'), 3.67 (dd, *J* = 12.2, 3.5 Hz, 1H, H-5''), 3.97 (q, *J* = 3.0 Hz, 1H, H-4'), 4.14 (br. s, 1H, H-3'), 4.68 (br. s, 1H, H-2'), 5.16 (d, *J* = 3.7 Hz, 1H, OH-3'), 5.40 (br. s, 1H, OH-2'), 5.71 (br. s, 1H, OH-5'), 5.93 (d, *J* = 6.4 Hz, 1H, H-1'), 6.61 (br. s, 1H), 7.97 (d, *J* = 5.2 Hz, 1H), 8.37 (s, 1H, H-8), 11.37 (br. s, 1H, NH). ^{13}C NMR (100 MHz, DMSO-d6): δ = 61.9 (C-5'), 70.9 (C-3'), 73.1 (C-2'), 86.0 (C-4'), 88.2 (C-1'), 106.0* (C1), 126.0** (C-5), 141.2 (C-8), 144.9 (C2), 148.3** (C-4), 158.0 (C=O (C-6)). HRMS (ESI): calculated for C11H14N3O5 ([M+H]$^{+}$): 268.0928, found: 268.0232. *data obtained from ^{1}H-^{13}C gHSQC. **data obtained from ^{1}H-^{13}C gHMBC.

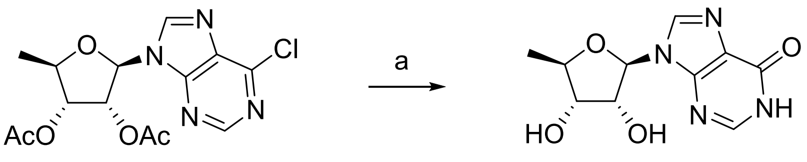

FH14908 **FH14911**

Scheme 2: reagents and conditions: a) 2-mercaptoethanol, NaOMe/MeOH, MeOH, reflux.

FH14908 was prepared according to Bookser et al., 2005. FH14908 (0.18 g, 0.51 mmol, 1 eq.) was suspended in MeOH (8.0 mL). Next, 2-mercaptoethanol (0.11 mL, 2.0 mmol, 4 eq.) was added, followed by NaOMe/MeOH solution (5.4 M, 0.38 mL, 2.0 mmol, 4 eq). The resulting mixture was heated to reflux overnight, and then cooled to ambient temperature. The solution was neutralized with 1 M aq. HCl and evaporated to dryness. The residue was purified by column chromatography 0 → 20% MeOH/DCM to give FH14911 (0.070 g, 0.28 mmol) as a white solid in 55% yield.

^{1}H NMR (400 MHz, MeOH-d4): δ = 1.42 (d, *J* = 6.2 Hz, 3H, CH3), 4.07 (t, *J* = 5.3 Hz, 1H, H-3'), 4.10 – 4.16 (m, 1H, H-4'), 4.67 (t, *J* = 5.09, 1H, H-2'), 5.97 (d, *J* = 4.6 Hz, 1H, H-1'), 8.07 (s, 1H, H-2), 8.21 (s, 1H, H-8). Spectral data are in accordance with literature values (Ciuffreda, P. et al. Eur. J. Org. Chem. 2003, 24, 4748-4751).

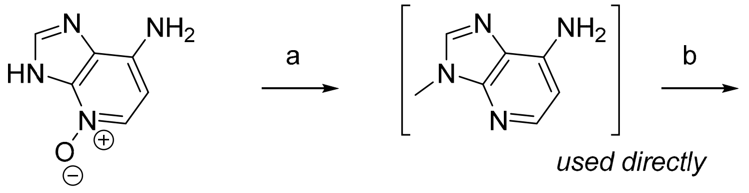

FH14860 **FH14866** **FH14872**

Scheme 3: a) i. K_2CO_3, MeI, DMF, 65 °C; ii. Fe/AcOH, 100 °C; b) $NaNO_2$, 5% aq. H_2SO_4 0 °C – 100 °C

Synthesis of FH14866-mixture:

FH14860 (0.15 g, 0.82 mmol, 1 eq.), prepared according to Hirao et al (2019), was dissolved in DMF (3.0 mL, 4 mL/mmol), and K_2CO_3 (0.23 g, 1.6 mmol, 2 eq.) was added. Then, MeI (0.065 mL, 0.98 mmol, 1.2 eq.) was added dropwise, and the resulting mixture heated to 65 °C overnight. After cooling to ambient temperature, water was added, followed by EA. The layers were separated, and the water layer extracted twice more with EA. The organic layers were combined, dried over Na_2SO_4, filtered and evaporated to dryness. The residue was taken up in AcOH (6.2 mL, 7.5 mL/mmol) and iron powder (Fe^0, 0.62 g, 0.75 g/mmol) was added. The resulting mixture was heated to 100 °C until LC/MS showed full conversion to the reduced product, after which the mixture was cooled in an ice bath, and aq. 2 M NaOH solution was added until pH>10. The solution was then filtered over Celite® and the filtrate was extracted four times with DCM. The organic layers were combined, dried over Na_2SO_4, filtered and evaporated. The residue was immediately used in the next step.

Synthesis of 3-Methyl-3,4-dihydro-7H-imidazo[4,5-b]pyridin-7-one (FH14872)

FH14866-mixture (0.030 g, 0.20 mmol, 1 eq.) was suspended in 5% aq. H_2SO_4 (1.0 mL, 6.0 mL/mmol) and the mixture was cooled to 0 °C and a solution of $NaNO_2$ (0.022 g, 0.31 mmol, 1.6 eq.) in water (0.19 mL, 0.60 mL/mmol) was added. The resulting solution was heated to 100 °C, after which a solution of 5% (V/V) aq. H_2SO_4 was added (1.0 mL, 6.0 mL/mmol). The mixture was stirred at 100 °C for 1 h and subsequently cooled to ambient temperature. After neutralization with saturated aq. $NaHCO_3$, the mixture was evaporated, and the residue purified by column chromatography (0 → 10% MeOH/EA) to give FH14872 (0.025 g, 0.17 mmol) as a slightly yellow solid in 83% yield.

^{1}H NMR (400 MHz, MeOH-d4): δ = 3.87 (s, 3H, CH3), 6.68 (d, J = 5.85 Hz, 1H, H-6), 8.07 (d, J = 8.81 Hz, 1H, H-5), 8.10 (s, 1H, H-2). ^{13}C NMR (100 MHz, MeOH-d4): δ = 30.4 (CH3), 106.8 (C-5), 126.3 (C-7a), 143.9 (C-2), 145.8 (C-6), 149.5 (C-3a), 159.4 (C-7). HRMS (ESI): calculated for C7H8N3O1 ($[M+H]^+$): 150.0662, found: 150.0065.

FH15983

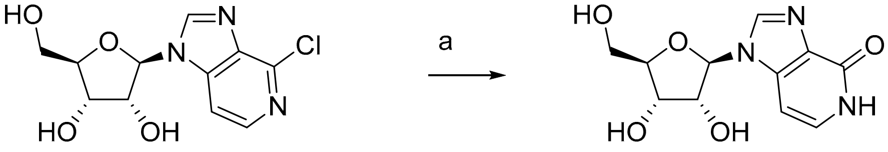

FH15977 **FH15983**

Scheme 4: reagents and conditions: 2-mercaptoethanol, NaOMe/MeOH, MeOH, reflux

FH15977 was prepared according to Zhang et al. (2018).

FH15977 (0.075 g, 0.26 mmol, 1 eq.) was dissolved in MeOH (5.0 mL), after which 2-mercaptoethanol (0.080 mL, 1.1 mmol, 3 eq.) and NaOMe/MeOH (5.4 M, 0.25 mL, 4 eq.) were added. The resulting mixture was heated at reflux temperature for 7 days. After cooling to ambient temperature, the mixture was neutralized with 1 M aq. HCl solution and evaporated. The residue was purified by column chromatography (20 → 40 % MeOH/EA) to give **FH15983** (0.050 g, 0.19 mmol) as a yellowish solid in 71 % yield.

^{1}H NMR (DMSO-d$_6$, 400MHz): δ = 3.56 – 3.67 (m, 2H, H-5', H-5''), 3.96 (q, *J* = 3.1 Hz, 1H, H-4'), 4.10 (br. s, 1H, H-3'), 4.22 – 4.26 (m, 1H, H-2'), 5.18 (t, *J* = 5.2 Hz, 1H, OH-5'), 5.31 (br. s, 1H, OH-3'), 5.62 (d, *J* = 5.2 Hz, 1H, OH-2'), 5.75 (d, *J* = 6.2 Hz, 1H, H-1'), 6.74 (d, *J* = 7.3 Hz, 1H, H-3), 7.17 (d, *J* = 5.7 Hz, 1H, H-2), 8.25 (s, 1H, H-2), 11.62 (br. s, 1H, NH). Spectral data are in accordance with literature values (Minakawa and Matsuda, 1993).

References:

Boehr DD, Farley AR, LaRonde FJ, Murdock TR, Wright GD, Cox JR (2005) Establishing the Principles of Recognition in the Adenine-Binding Region of an Aminoglycoside Antibiotic Kinase [APH(3')-IIIa]. Biochemistry 44, 12445–12453. doi:10.1021/bi051085p

Bookser BC, Matelich MC, Ollis K, Ugarkar BG (2005) Adenosine kinase inhibitors. 4. 6, 8-Disubstituted purine nucleoside derivatives. Synthesis, conformation, and enzyme inhibition. J Med Chem 48, 3389–3399. doi:10.1021/jm048968j

Ciuffreda P, Loseto A, Alessandrini L, Terraneo G, Santaniello E (2003) Adenylate Deaminase (5'-Adenylic Acid Deaminase, AMPDA)-Catalyzed Deamination of 5'-Deoxy-5'-Substituted and 5'-Protected Adenosines: A Comparison with the Catalytic Activity of Adenosine Deaminase (ADA). Eur. J. Org. Chem. 24, 4748–4751. doi: 10.1002/ejoc.200300435

Hirao, Y., Seo, S; Kubo, T. The Journal of Physical Chemistry C (2019) Self-assembly of 1-deazahypoxanthine: cooperativity of hydrogen-bonding and stacking interactions. *J. Phys. Chem. C* 123, 34, 20928–20935.

Minakawa N, Matsuda N (1993) Nucleosides and nucleotides. 116. Convenient syntheses of 3-deazaadenosine, 3-deazaguanosine, and 3-deazainosine via ring closure of 5-ethynyl-1-β-D-

ribofuranosylimidazole-4-carboxamide or -carbonitrile. Tetrahedron 49, 557–570. doi: 10.1016/S0040-4020(01)86259-1

Zhang B, De Graef S, Nautiyal M, Pang L, Gadakh B, Froeyen M, Van Mellaert L, Strelkov SV, Weeks SD, Van Aerschot A. (2018) Family-wide analysis of aminoacyl-sulfamoyl-3-deazaadenosine analogues as inhibitors of aminoacyl-tRNA synthetases. Eur J Med Chem. 148:384–396. doi: 10.1016/j.ejmech.2018.02.013.
