# Supplementary material for: A Toxoplasma gondii Oxopurine Transporter Binds Nucleobases and Nucleosides Using Different Binding Modes
Source: Int J Mol Sci. 2022 Jan 10;23(2):710. doi: 10.3390/ijms23020710 (PMC8776092; doi:10.3390/ijms23020710)
Supplement: Supplementary file 1 [file ijms-23-00710-s001.zip › Supplemental Table S1 source of nucleosides.pdf]

**Table S1. Source of nucleoside and nucleobase analogues used in this study.**

| | Supplier |
|---|---|
| *Purine nucleobases* | |
| Guanine | Sigma |
| Hypoxanthine | Sigma |
| Xanthine | Sigma |
| Adenine | Sigma |
| 6-Chloropurine | Aldrich |
| 6-Mercaptopurine | Aldrich |
| 6-Thioguanine | Sigma Life Science |
| 1-deazahypoxanthine (FH14875) | Van Calenbergh lab |
| 1-methylhypoxanthine | AKOS |
| 2,6-diaminopurine | Sigma |
| 3-deazahypoxanthine | Fluorochem UK (catalogue number 331062) |
| 3-methylxanthine | Aldrich |
| 7-deazahypoxanthine | Alfa Aesar |
| 7-deazaguanine | Sigma |
| Allopurinol | Sigma |
| 7-Br-allopurinol (JBMAM034) | Van Calenbergh lab |
| Aminopurinol | Sigma Aldrich |
| 8-azahypoxanthine | Sigma |
| 9-deazahypoxanthine | Biosynth Carbosynth |
| 9-deazaguanine (JBMAM002) | Van Calenbergh lab |
| 9-deazaxanthine | Gift from Dr Howard B. Cottam, University of California, San Diego |
| 9-Me,1-deazahypoxanthine (FH14872) | Van Calenbergh lab |
| *Purine nucleosides* | |

| | |
|---|---|
| Guanosine | Sigma |
| Inosine | Sigma |
| Adenosine | Sigma |
| 2’-deoxyinosine | Biosynth Carbosynth |
| 3’-deoxyinosine | Biosynth Carbosynth |
| 5’-deoxyinosine (FH14911) | Van Calenbergh lab |
| 2’-deoxyguanosine | Sigma |
| 3’-deoxyguanosine | Biosynth Carbosynth |
| 2’-deoxyadenosine | Sigma |
| 3’-deoxyadenosine (FH8465) | Van Calenbergh lab |
| 5’-deoxyadenosine | Sigma |
| 3’-deoxy,7-deazaadenosine (FH7429_D) | Van Calenbergh lab |
| Adenine arabinoside (Ara-A) | Biosynth Carbosynth |
| 2’,3’-dideoxyinosine | Biosynth Carbosynth |
| 1-deazainosine (FH15949) | Van Calenbergh lab |
| 1-deazaadenosine | Tocris |
| 3-deazaadenosine (FH15978) | FH15978 |
| Nebularine | Sigma Aldrich |
| 6-thioinosine | Biosynth Carbosynth |
| 6-O-methyl,7-deaza,7-Cl-inosine (FH8446) | Van Calenbergh lab |
| 6-O-ethyl,7-deaza,7-Cl-inosine (FH9529) | Van Calenbergh lab |
| 7-deazainosine (FH15951) | Van Calenbergh lab |
| 7-deaza-7-Chloroinosine (JB546) | Van Calenbergh lab |
| 7-deaza-7-Bromoinosine (JB464, FH14864) | Van Calenbergh lab |
| 7-deaza-2’-deoxyinosine | Biosynth Carbosynth |
| 7-deaza-3’-deoxyinosine (FH9560) | Van Calenbergh lab |
| NBMPR | Sigma Aldrich |
