# Supplementary material for: A Toxoplasma gondii Oxopurine Transporter Binds Nucleobases and Nucleosides Using Different Binding Modes
Source: Int J Mol Sci. 2022 Jan 10;23(2):710. doi: 10.3390/ijms23020710 (PMC8776092; doi:10.3390/ijms23020710)
Supplement: Supplementary file 1 [file ijms-23-00710-s001.zip › Supplemental Table S2.pdf]

Supplemental Table S2. Structures and references of compounds synthesized for this study.

Compound name	Compound structure	Status
**FH15978**	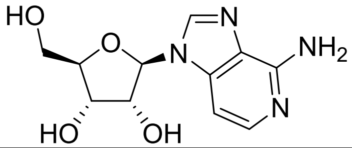	**Published**<br>Zhang, B. et al. 2018
**FH7429_D**	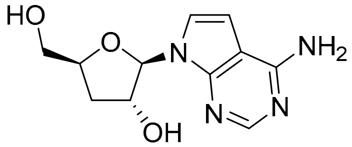	**Published**<br>Hulpia et al. 2019
**FH15949**	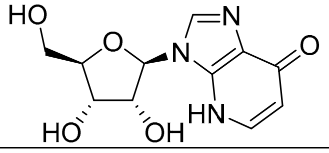	**NEW**<br>New synthesis, see Supplemental Information File 1
**FH15983**	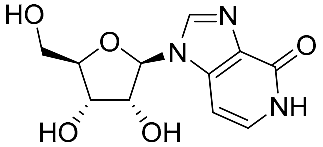	**Published**<br>Minakawa and Matsuda, 1993<br><br>New synthetic route (see Supplemental Information File 1)
**FH9560**	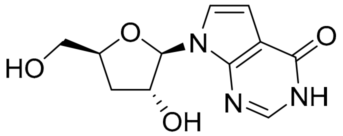	**Published**<br>Hulpia et al. ACS Infect Dis 2020
**FH15951**	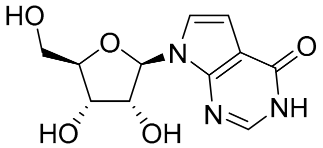	**Published**<br>Hulpia et al. Eur J Med Chem 2020
**JB464/FH14864**	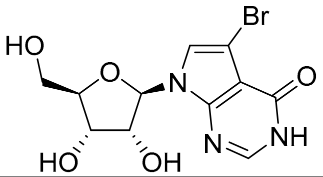	**Published**<br>Hulpia et al. Eur J Med Chem 2020
**JB546**	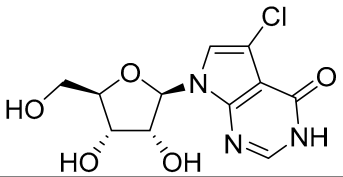	**Published**<br>Hulpia et al. Eur J Med Chem 2020
**FH14911**	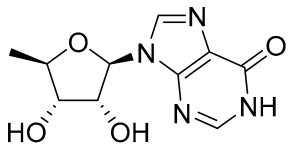	**Published**<br>Ciuffreda et al. Eur. J. Org. Chem. 2003<br><br>New synthetic route (see Supplemental Information File 1)

**FH8446**	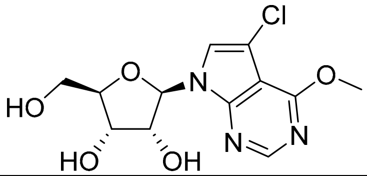	**Published**<br>Hulpia et al. Eur J Med Chem 2020
**FH9529**	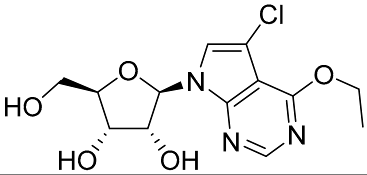	**Published**<br>Hulpia et al. Eur J Med Chem 2020
**JBMAM034**	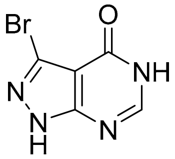	**Published**<br>Chu and Lynch J Med Chem 1975<br>Hirst, G. et al.<br>WO 2002080926 A1<br>(ref in file)
**FH14872**	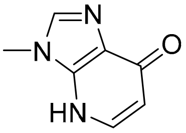	**New compound**<br>For synthesis, see Supplemental Information File 1.
**FH14875**	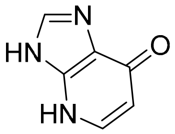	**Published**<br>Hirao. et al.<br>J. Phys. Chem. C. 2019

References

Chu I, Lynch BM (1975) Synthesis and biological evaluation of xanthine oxidase inhibitors. Pyrazolo(3,4-d)pyrimidines and pyrazolo(3,4-b)pyridines. J Med Chem. 18, 161-165. doi: 10.1021/jm00236a010.

Ciuffreda P, Loseto A, Alessandrini L, Terraneo G, Santaniello E (2003) Adenylate Deaminase (5′-Adenylic Acid Deaminase, AMPDA)-Catalyzed Deamination of 5′-Deoxy-5′-Substituted and 5′-Protected Adenosines: A Comparison with the Catalytic Activity of Adenosine Deaminase (ADA). Eur. J. Org. Chem. 24, 4748–4751. doi: 10.1002/ejoc.200300435

Hirao Y, Seo S, Kubo T (2019) Self-Assembly of 1-Deazahypoxanthine: Cooperativity of Hydrogen-Bonding and Stacking Interactions. J Phys Chem C 123, 20928–20935

Hirst GC, Rafferty P, Ritter K, Calderwood D, Wishart N, Arnold LD, Friedman MM. Pyrazolopyrimidines as therapeutic agents. patent WO2002080926A1

Hulpia, F, Bouton J, Campagnaro GD, Alfayez IA, Mabille D, Maes L, De Koning HP, Caljon G, Van Calenbergh S. (2020) C6-*O*-Alkylated 7-deaza inosine nucleoside analogues: Discovery of potent and selective anti-sleeping sickness agents. Eur J Med Chem 18:112018 doi: 10.1016/j.ejmech.2019.112018

Hulpia F, Campagnaro GD, Alzahrani KJ, Alfayez IA, Ungogo MA, Mabille D, Maes L, De Koning HP, Caljon G, Van Calenbergh (2020) Structure-activity relationship exploration of 3’-deoxy-7-deazapurine nucleoside analogues as anti-*Trypanosoma brucei* agents. ACS Infect Dis 6:2045-2056. doi: 10.1021/acsinfecdis.0c00105

Hulpia F, Mabille D, Campagnaro GD, Schumann G, Maes L, Roditi I, Hofer A, de Koning HP, Caljon G, Van Calenbergh S. (2019) Combining tubercidin and cordycepin scaffolds results in highly active candidates to treat late-stage sleeping sickness. Nat Commun. 10:5564. doi: 10.1038/s41467-019-13522-6.

Minakawa N, Matsuda N (1993) Nucleosides and nucleotides. 116. Convenient syntheses of 3-deazaadenosine, 3-deazaguanosine, and 3-deazainosine via ring closure of 5-ethynyl-1-β-D-ribofuranosylimidazole-4-carboxamide or -carbonitrile.Tetrahedron 49, 557–570. doi: 10.1016/S0040-4020(01)86259-1

Zhang B, De Graef S, Nautiyal M, Pang L, Gadakh B, Froeyen M, Van Mellaert L, Strelkov SV, Weeks SD, Van Aerschot A. (2018) Family-wide analysis of aminoacyl-sulfamoyl-3-deazaadenosine analogues as inhibitors of aminoacyl-tRNA synthetases. Eur J Med Chem. 148:384–396. doi: 10.1016/j.ejmech.2018.02.013.
