# Supplementary material for: A Toxoplasma gondii Oxopurine Transporter Binds Nucleobases and Nucleosides Using Different Binding Modes
Source: Int J Mol Sci. 2022 Jan 10;23(2):710. doi: 10.3390/ijms23020710 (PMC8776092; doi:10.3390/ijms23020710)
Supplement: Supplementary file 1 [file ijms-23-00710-s001.zip › Supplemental Table S3 - structures of purines and purine analogues.pdf]

Purine nucleobases	
Guanine	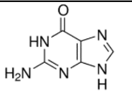
Hypoxanthine	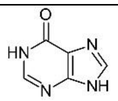
Xanthine	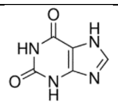
Adenine	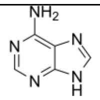
6-Chloropurine	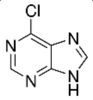
6-Mercaptopurine	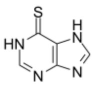
6-Thioguanine	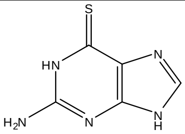
1-deazahypoxanthine	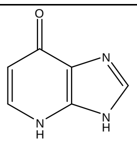
1-methylhypoxanthine	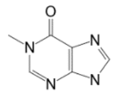
2,6-diaminopurine	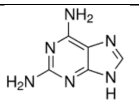
3-deazahypoxanthine	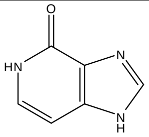

3-methylxanthine	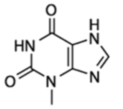
7-deazahypoxanthine	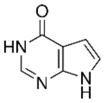
7-deazaguanine	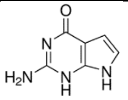
Allopurinol	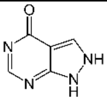
7-Br-allopurinol	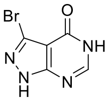
Aminopurinol	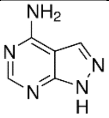
8-azahypoxanthine	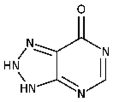
9-deazahypoxanthine	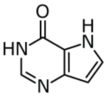
9-deazaguanine	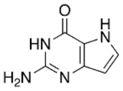
9-deazaxanthine	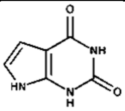
9-Me,1-deazahypoxanthine	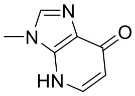
Purine nucleosides	

Guanosine	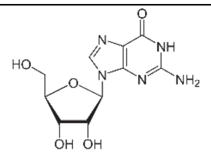
Inosine	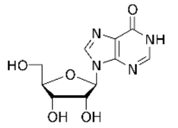
Adenosine	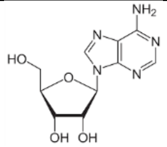
2′-deoxyinosine	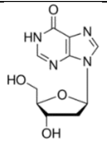
3′-deoxyinosine	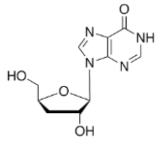
2′,3′-dideoxyinosine	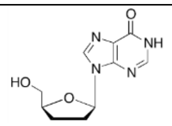
5′-deoxyinosine	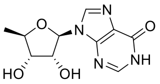
2′-deoxyguanosine	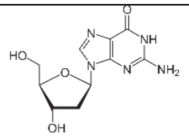
3′-deoxyguanosine	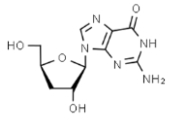
2′-deoxyadenosine	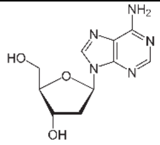

3′-deoxyadenosine	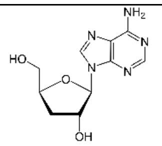
5′-deoxyadenosine	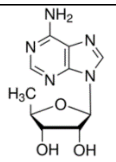
3′-deoxy,7-deazaadenosine	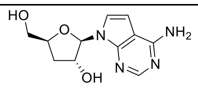
Adenine arabinoside (Ara-A)	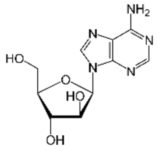
1-deazainosine	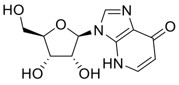
1-deazaadenosine	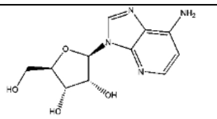
3-deazaadenosine	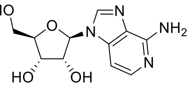
Nebularine	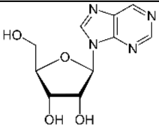
6-thioinosine	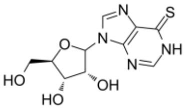
6-O-methyl,7-deaza,7-Cl-inosine	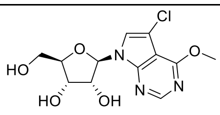
6-O-ethyl,7-deaza,7-Cl-inosine	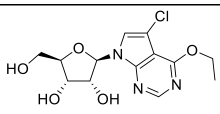

7-deazainosine	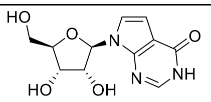
7-deaza-7-Chloroinosine	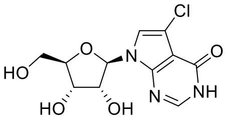
7-deaza-7-Bromoinosine	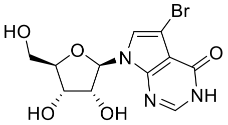
7-deaza-2′-deoxyinosine	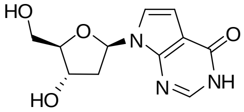
7-deaza-3′-deoxyinosine	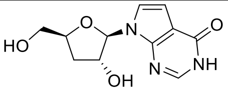
S-(4-nitrobenzyl)-6-thioinosine (NBMPR)	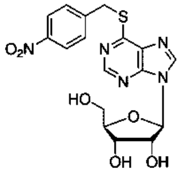
