# Supplementary material for: A Toxoplasma gondii Oxopurine Transporter Binds Nucleobases and Nucleosides Using Different Binding Modes
Source: Int J Mol Sci. 2022 Jan 10;23(2):710. doi: 10.3390/ijms23020710 (PMC8776092; doi:10.3390/ijms23020710)
Supplement: Supplementary file 1 [file ijms-23-00710-s001.zip › Supplemental Table S4 - Sequences used in the phylogenetic analyses.pdf]

Supplemental Table S4. Sequences used in the phylogenetic analysis.

| **Transporter family** | **Species** | **GeneID** | **Database** |
|---|---|---|---|
| ENT | *Trypanosoma brucei* | Tb927.2.6240 | TritrypDB |
| | *Trypanosoma brucei* | Tb927.2.6280 | TritrypDB |
| | *Trypanosoma brucei* | Tb927.2.6320 | TritrypDB |
| | *Trypanosoma brucei* | Tb927.2.6200 | TritrypDB |
| | *Trypanosoma brucei* | Tb927.2.6150 | TritrypDB |
| | *Trypanosoma brucei* | Tb927.2.6220 | TritrypDB |
| | *Trypanosoma brucei* | Tb927.6.220 | TritrypDB |
| | *Trypanosoma brucei* | Tb927.9.7470 | TritrypDB |
| | *Trypanosoma brucei* | Tb927.3.590 | TritrypDB |
| | *Trypanosoma brucei* | Tb927.5.286b | TritrypDB |
| | *Trypanosoma brucei* | Tb09.v4.0106 | TritrypDB |
| | *Trypanosoma brucei* | Tb927.9.15980 | TritrypDB |
| | *Trypanosoma brucei* | Tb927.11.6310 | TritrypDB |
| | *Trypanosoma brucei* | Tb927.11.6320 | TritrypDB |
| | *Trypanosoma brucei* | Tb927.11.6360 | TritrypDB |
| | *Leishmania major* | LmjF.36.1940 | TritrypDB |
| | *Leishmania major* | LmjF.11.0550 | TritrypDB |
| | *Leishmania major* | LmjF.13.1210 | TritrypDB |
| | *Leishmania major* | LmjF.15.1230 | TritrypDB |
| | *Leishmania major* | LmjF.15.1240 | TritrypDB |
| | *Leishmania mexicana* | LmxM.36.1940 | TritrypDB |
| | *Leishmania mexicana* | LmxM.11.0550 | TritrypDB |
| | *Leishmania mexicana* | LmxM.13.1210 | TritrypDB |

| | | | |
|---|---|---|---|
| | *Leishmania mexicana* | LmxM.15.1230 | TritrypDB |
| | *Leishmania mexicana* | LmxM.15.1240 | TritrypDB |
| | *Leishmania donovani* | LdBPK_362040.1 | TritrypDB |
| | *Leishmania donovani* | LdBPK_110520.1 | TritrypDB |
| | *Leishmania donovani* | LdBPK_131110.1 | TritrypDB |
| | *Leishmania donovani* | LdBPK_151230.1 | TritrypDB |
| | *Homo sapiens* | NP_001071645.1 | Genbank |
| | *Homo sapiens* | NP_001287797.1 | Genbank |
| | *Homo sapiens* | NP_060814.4 | Genbank |
| | *Homo sapiens* | NP_001035751.1 | Genbank |
| | *Plasmodium falciparum* | PF3D7_1469400 | PlasmoDB |
| | *Plasmodium falciparum* | PF3D7_0103200 | PlasmoDB |
| | *Plasmodium falciparum* | PF3D7_0824400 | PlasmoDB |
| | *Plasmodium falciparum* | PF3D7_1347200 | PlasmoDB |
| | *Hammondia hammondi* | HHA_288540 | ToxoDB |
| | *Hammondia hammondi* | HHA_233130 | ToxoDB |
| | *Hammondia hammondi* | HHA_450020 | ToxoDB |
| | *Hammondia hammondi* | HHA_244440 | ToxoDB |
| | *Toxoplasma gondii* | TGVEG_288540 | ToxoDB |
| | *Toxoplasma gondii* | TGVEG_233130 | ToxoDB |
| | *Toxoplasma gondii* | TGVEG_359630 | ToxoDB |
| | *Toxoplasma gondii* | TGVEG_244440 | ToxoDB |
| | *Cystoisospora suis* | CSUI_011115 | ToxoDB |
| | *Cystoisospora suis* | CSUI_005052 | ToxoDB |
| | *Cystoisospora suis* | CSUI_002055 | ToxoDB |
| | *Cystoisospora suis* | CSUI_008365 | ToxoDB |

| | | | |
|---|---|---|---|
| | *Sarcocystis neurona* | SN3_00200406 | ToxoDB |
| | *Sarcocystis neurona* | SN3_00701320 | ToxoDB |
| NAT/NCS2 | *Arabidopsis thaliana* | Q8VZQ5.1 | Genbank |
| | *Arabidopsis thaliana* | Q0WPE9.2 | Genbank |
| | *Arabidopsis thaliana* | Q27GI3.2 | Genbank |
| | *Arabidopsis thaliana* | Q8RWE9.1 | Genbank |
| | *Arabidopsis thaliana* | P93039.2 | Genbank |
| | *Arabidopsis thaliana* | Q3E956.1 | Genbank |
| | *Arabidopsis thaliana* | O04472.2 | Genbank |
| | *Arabidopsis thaliana* | Q8FZD4.2 | Genbank |
| | *Arabidopsis thaliana* | Q94C70.2 | Genbank |
| | *Arabidopsis thaliana* | Q9SHZ3.1 | Genbank |
| | *Arabidopsis thaliana* | Q6SZ87.1 | Genbank |
| | *Arabidopsis thaliana* | Q3E7D0.3 | Genbank |
| | *Homo sapiens* | NP005838.3 | Genbank |
| | *Homo sapiens* | NP_976072.1 | Genbank |
| CNT | *Homo sapiens* | AAL09447.1 | Genbank |
| | *Homo sapiens* | NP_004203.2 | Genbank |
| | *Homo sapiens* | AAG22551.1 | Genbank |
| | *Vibrio cholerae* | 3TIJ | Genbank |
| AzgA-like | *Aspergillus nidulans* | AN8534 | AspGD |
| | *Aspergillus fumigatus* | Afu5g09750 | AspGD |
| NCS1 | *Saccharomyces cerevisae* | KZV11811.1 | Genbank |
| | *Aspergillus nidulans* | C8V329.1 | Genbank |
| | *Aspergillus nidulans* | AN6783 | AspGD |
| | *Escherichia coli* | EFF14147.1 | Genbank |

| | | | |
|---|---|---|---|
| | *Arabidopsis thaliana* | Q9LZD0.1 | Genbank |
| | *Setaria viridis* | AHC53692.1 | Genbank |
| | *Aspergillus nidulans* | ABR22526.1 | Genbank |
| | *Saccharomyces cerevisae* | CAA84963.1 | Genbank |
| | *Saccharomyces cerevisae* | CAA84862.1 | Genbank |
